# A promiscuous cytochrome P450 aromatic *O*-demethylase for lignin bioconversion

**DOI:** 10.1038/s41467-018-04878-2

**Published:** 2018-06-27

**Authors:** Sam J. B. Mallinson, Melodie M. Machovina, Rodrigo L. Silveira, Marc Garcia-Borràs, Nathan Gallup, Christopher W. Johnson, Mark D. Allen, Munir S. Skaf, Michael F. Crowley, Ellen L. Neidle, Kendall N. Houk, Gregg T. Beckham, Jennifer L. DuBois, John E. McGeehan

**Affiliations:** 1 Molecular Biophysics, School of Biological Sciences, Institute of Biological and Biomedical Sciences, University of Portsmouth, Portsmouth, PO1 2DY UK; 2 National Bioenergy Center, National Renewable Energy Laboratory, Golden, CO 80401 USA; 3 Department of Chemistry and Biochemistry, Montana State University, Bozeman, MT 59717 USA; 4 Institute of Chemistry, University of Campinas, Campinas, Sao Paulo 13083-970 Brazil; 5 Department of Chemistry and Biochemistry, University of California at Los Angeles, Los Angeles, CA 90095 USA; 6 Biosciences Center, National Renewable Energy Laboratory, Golden, CO 80401 USA; 7 Department of Microbiology, University of Georgia, Athens, GA 30602 USA

## Abstract

Microbial aromatic catabolism offers a promising approach to convert lignin, a vast source of renewable carbon, into useful products. Aryl-*O*-demethylation is an essential biochemical reaction to ultimately catabolize coniferyl and sinapyl lignin-derived aromatic compounds, and is often a key bottleneck for both native and engineered bioconversion pathways. Here, we report the comprehensive characterization of a promiscuous P450 aryl-*O*-demethylase, consisting of a cytochrome P450 protein from the family CYP255A (GcoA) and a three-domain reductase (GcoB) that together represent a new two-component P450 class. Though originally described as converting guaiacol to catechol, we show that this system efficiently demethylates both guaiacol and an unexpectedly wide variety of lignin-relevant monomers. Structural, biochemical, and computational studies of this novel two-component system elucidate the mechanism of its broad substrate specificity, presenting it as a new tool for a critical step in biological lignin conversion.

## Abstract (web summary)

Catabolizing lignin-derived aromatic compounds requires an aryl-*O*-demethylation step. Here the authors present the structures of GcoA and GcoB, a cytochrome P450-reductase pair that catalyzes aryl-*O*-demethylations and show that GcoA displays broad substrate specificity, which is of interest for biotechnology applications.

## Introduction

Lignin is a heterogeneous, aromatic biopolymer found in abundance in plant cell walls where it is used for defense, structure, and nutrient and water transport^1^. Given its prevalence in plant tissues, lignin is the largest reservoir of renewable, aromatic carbon found in nature. The ubiquitous availability of lignin in the environment, coupled to its inherent structural heterogeneity and complexity, has led to the evolution of microbial strategies to break lignin polymers down to smaller fragments using powerful oxidative enzymes secreted by rot fungi and some bacteria^2–4^. These lignin oligomers can be further assimilated as carbon and energy sources, through at least four known catabolic paradigms^5^.

**Fig. 1:**
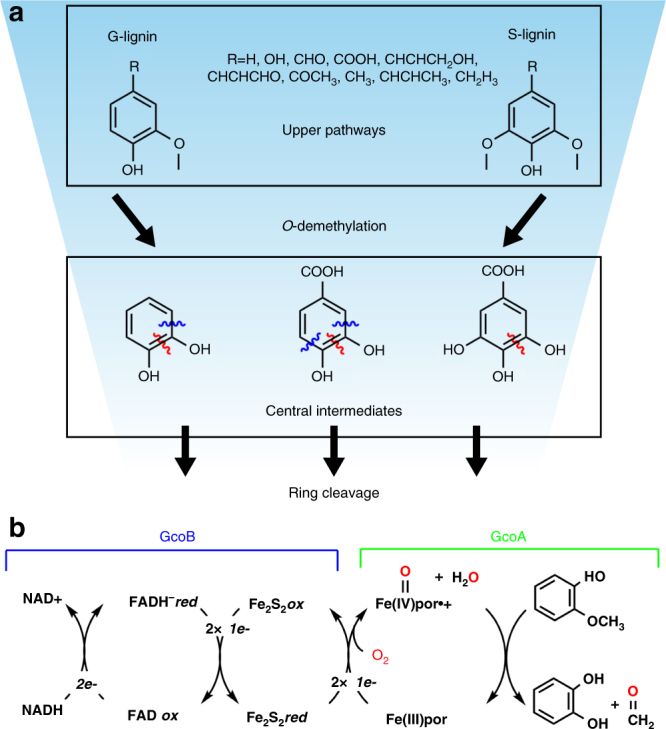
*O*-demethylation in aromatic catabolism. **a** *O*-demethylation provides a central role in the upper pathways of aromatic catabolism^5,6,76–80^. G- and S-lignin, the primary units in lignin, are *O*-demethylated to form central intermediates. These are then cleaved by intradiol (red lines) or extradiol (blue lines) dioxygenases. **b** Coupled reactions catalyzed by GcoA and GcoB. por, porphyrin

The most well understood aromatic catabolic mechanism, mainly studied in aerobic soil bacteria, relies on the use of non-heme iron-dependent dioxygenases to oxidatively ring-open structurally diverse, lignin-derived aromatic compounds^5,6^. These dioxygenases act on central intermediate substrates, such as catechol, protocatechuate, and gallate, either in an intra- or extra-diol manner. Lignin is primarily based on coniferyl (G) and sinapyl (S) alcohol subunits, which exhibit one or two methoxy groups on the aromatic ring, respectively. Nearly all lignin-derived compounds must therefore be *O*-demethylated to diols before they can be oxidatively cleaved to generate ring-opened compounds, which are ultimately routed to central carbon metabolism (Fig. 1)^7^. More recently, the same aromatic-catabolic pathways have been invoked as a potential means to convert lignin to useful products in biorefineries^4,7–11^. *O*-demethylation is therefore a critical reaction for assimilating lignin-derived carbon in both natural carbon cycling as well as in emerging biotechnology applications.

The importance of *O*-demethylation has motivated substantial efforts toward the discovery and characterization of enzymes capable of demethylating the methoxy substituents of diverse lignin-derived substrates^12–20^. For example, Ornston et al. described the VanAB *O*-demethylase in *Acinetobacter baylyi* ADP1, which converts vanillate to the central intermediate, protocatechuate, via a Rieske non-heme iron monooxygenase mechanism^14,15^. VanAB, which is common in many aromatic-catabolic soil bacteria, is active on vanillate analogs, but to our knowledge, has not been reported to be active on other lignin-derived compounds. Masai and colleagues first described LigX^18^ from *Sphingobium* sp. SYK-6, a model bacterium for aromatic catabolism^7^. LigX also employs a Rieske non-heme iron monooxygenase mechanism to demethylate a biphenyl compound representing a common lignin linkage. Masai et al. additionally reported, in SYK-6, two tetrahydrofolate-dependent *O*-demethylases, LigM and DesA. LigM primarily demethylates vanillate and 3-*O*-methylgallate, whereas DesA principally demethylates syringate with very weak activity on vanillate^16,17^.

Earlier reports from Eltis et al. and Bell et al. described cytochrome P450-based demethylation of aromatic compounds; though either the full gene sequences were not reported until recently^13,21^, or the *para*-substituted substrate (4-methoxybenzoate) was of limited interest for the lignin degradation problem^22,23^. Similarly, Dardas et al. found evidence of a P450 in *Moraxella* GU2 responsible for the *O*-demethylation of guaiacol and guaethol; however, neither the gene sequence nor identity of the P450 or its reductase partner was isolated^24^.

The relatively narrow substrate specificities elucidated to date for aryl-*O*-demethylation, coupled to the potentially broad distribution of structurally distinct, methoxylated lignin products found in nature, prompted us to search for alternative mechanisms for this key reaction. Because G-unit monomers constitute a majority of plant-derived lignin, we initially focused on *O*-demethylation of guaiacol (2-methoxyphenol), which in turn represents the simplest G-unit monomer derivable from lignin. As reported in a companion study^21^, we isolated a cytochrome P450-reductase gene pair, *gcoAB*, from *Amycolatopsis* sp. ATCC 39116 (encoding proteins with accession numbers WP_020419855.1 and WP_020419854.1). Introduction of this pair via plasmid-based expression into *Pseudomonas putida* KT2440, a robust aromatic-catabolic bacterium, was sufficient to confer growth on guaiacol^21^. Here, we report a comprehensive structural, biochemical, and computational description of this new cytochrome P450-based mechanism for aryl-*O*-demethylation. Unlike other known tetrahydrofolate- or non-heme iron-dependent demethylases, which are fairly substrate specific, the P450-reductase pair characterized here (GcoAB) demethylates diverse aromatic substrates, potentially providing an important advantage in both natural and biotechnological contexts. The results presented here suggest a remarkably flexible active site that may promote promiscuous substrate usage.

## Results

### GcoA crystal structures suggest broad substrate specificity

**Fig. 2:**
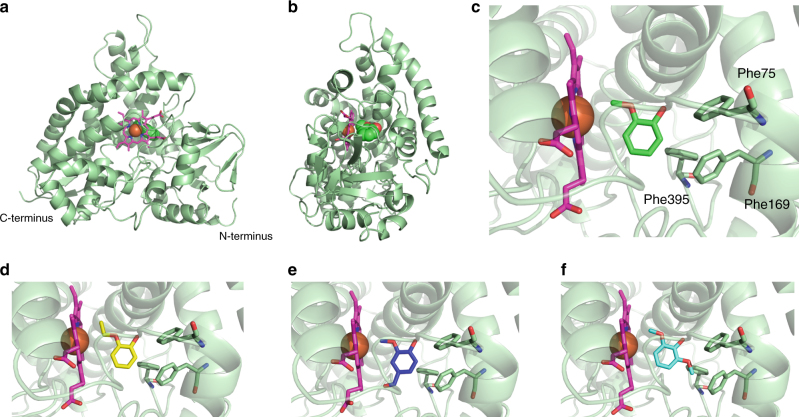
Crystal structures of GcoA, illustrating the substrate binding mode. **a**, **b** The general architecture of GcoA is shown in cartoon representation highlighting the relative positions of the buried heme (pink) and bound guaiacol (space-filling). **c**–**f** Comparisons of ligand-bound structures of guaiacol (green), guaethol (yellow), vanillin (blue), and syringol (cyan) showing key hydrophobic residues lining the active site pocket

**Fig. 3:**
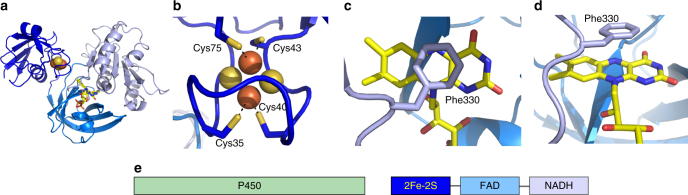
Crystal structure of GcoB. **a** The three-domain structure of GcoB is shown with electron transport cofactors. The N-terminal 2Fe-2S domain is shown in dark blue, followed by the FAD-binding domain in cyan, and the NADH-binding domain in light blue. **b** The 2Fe-2S cluster is held in an H-bonded basket coordinated by four Cys residues. **c**, **d** The FAD is accommodated by hydrophobic stacking interactions between Phe330 and the flavin isoalloxazine moiety. **e** A diagrammatic representation of the GcoAB domain organization

The X-ray crystal structures of GcoA (in complex with guaiacol) and GcoB were determined to resolutions of 1.4 Å and 1.7 Å, respectively (Figs. 2 and 3, Supplementary Figs. 1–7, and Supplementary Table 2). The GcoA structure reveals a typical P450 single-domain architecture with a central heme adjacent to a buried active site, captured with the substrate access loop in the closed position (Fig. 2a, b). GcoA possesses a broadly hydrophobic pocket with the two oxygen atoms of the substrate coordinated by backbone carbonyl and amide nitrogen groups from residues Val241 and Gly245, respectively. A series of hydrophobic amino acids is responsible for positioning the aromatic ring, including a triad of phenylalanine residues lining the active site cavity (Fig. 2c). The guaiacol methoxy carbon is positioned at a distance of 3.92 Å from the center of the heme iron, with the plane of the heme appearing to exhibit a slightly domed architecture.

Crystallization screening for a range of guaiacol-analog-bound complexes produced three additional well-diffracting co-crystals with guaethol, vanillin, and syringol, where each ligand was fully occupied in the active site (Fig. 2d–f). In each structure, a variety of alternative means to accommodate the additional aryl side chains were observed in the active site. Guaethol, the most similar chemical structure to guaiacol, is easily accommodated with a small translation of the ring and without any rearrangement of the active site around the additional methyl group. Vanillin, being bulkier with an aldehyde group at the 4-position, induces a significant reorientation of Thr296, forming a new H-bond between the residue and the substrate. This results in the twisting of the carboxylate group on the adjacent heme propionate, eliminating the H-bond with Thr296 observed in the guaiacol-bound structure, and instead forming an alternative H-bond with a water molecule in between the two heme propionates. In contrast, syringol is accommodated by a combination of a rotation in the plane of the aromatic ring about the hydroxyl group relative to guaiacol, and an expansion of the hydrophobic residues lining the active site cavity.

### GcoB has an unusual three-domain structure

Most bacterial cytochrome P450s require two partner proteins, usually a cytochrome P450 reductase or a ferredoxin and ferredoxin reductase, that transfer electrons from a carrier such as NAD(P)H to the cytochrome^25^. Analysis of the *GcoB* sequence, however, suggests it contains all of the necessary domains in a single polypeptide, with an N-terminal 2Fe-2S ferredoxin domain followed by an FAD and NAD(P) binding region with homology to ferredoxin-NADPH reductase (FNR) type oxido-reductases.

Structurally, the compact, N-terminal 2Fe-2S domain of GcoB (Fig. 3a, b) bears strong homology to putidaredoxin (Pdx), a ferredoxin that transfers electrons to P450cam in the three-component camphor hydroxylase system from *P. putida* (Supplementary Fig. 6–8). The C-terminal region consists of an FAD-binding domain, containing 6 beta-strands and a single alpha helix, followed by an NADH-binding domain. These C-terminal domains show structural homology to FAD-type cytochrome P450 reductase (CPR^26^) domains in which the C-terminal portion primarily stabilizes the isoalloxazine moiety of FAD while the N-terminal domain coordinates the diphosphate bridge between the flavin and adenosine groups (Fig. 3c, d). A structural comparison of the NADH-binding domain with the NADPH binding domain from a related CPR is strongly predictive of a binding preference for NADH over NADPH. While the individual domains are structurally similar to other CPR proteins, the overall domain architecture is not, and instead it is highly conserved with reductase proteins such as BenC^27^, the benzoate 1,2-dioxygenase reductase from *A. baylyi* ADP1, which supplies electrons to Rieske-type aromatic ring-hydroxylating dioxygenases. This suggests an unexpected convergence in the organization of the reductase partners for these evolutionarily distinct oxyenases.

### GcoA and GcoB form a dimer complex in solution

GcoA and GcoB operate as a multi-domain complex; guaiacol is processed exclusively in GcoA, with electrons supplied by the GcoB redox machinery. When expressed and purified individually, each protein was shown to be monomeric in solution using a combination of hydrodynamic methods (Supplementary Table 3). Size-exclusion chromatography revealed a strong interaction between GcoA and GcoB (Supplementary Fig. 9) which was confirmed by analytical ultracentrifugation, indicating that GcoAB is a heterodimer in solution. GcoA has a characteristic basic pocket on the proximal face, previously identified in other P450 systems^28–30^ as the docking surface for the associated reductase partner (Supplementary Fig. 10). Similarly, the surface of GcoB is predicted to have an acidic patch that interfaces with the corresponding basic region in GcoA^30^. From the GcoB crystal structure and surface charge representation, and comparison to ferredoxins homologous to GcoB^21^, it is likely that the interacting face of GcoB is buried at the interface between the ferredoxin domain and the FAD binding domain in the reported crystal structure. Drawing parallels from Huang et al.^31^, a conformational change of GcoB likely precedes binding with GcoA. A possible schematic for this process is shown in Supplementary Fig. 11. While the organization of P450 systems is diverse^32^, the architecture of this 2-protein system, combining a catalytic P450 with a 2Fe-2S ferredoxin domain in addition to FAD and NAD binding sites, represents a new class. The linear domain diagram shown in Fig. 3e allows comparison to standard class descriptions as reviewed by Guengerich and Munro, and illustrates the distinct nature of GcoAB^33^.

### GcoAB efficiently demethylates multiple aromatic substrates

The catalytic cofactors in GcoA and GcoB were spectroscopically characterized prior to describing the reactivity of the enzyme pair (Supplementary Fig. 12–15). The UV/visible spectrum of GcoA exhibits a sharp heme Soret peak at 417 nm and α- and β-bands (Q-bands) at 537 and 567 nm. GcoB has an absorbance maximum at 454 nm, indicative of oxidized FAD, and peaks at 423 nm and 480 nm most likely due to the 2Fe-2S cluster^34^. Total heme, FAD, and 2Fe-2S occupancies for the protein monomers were determined at 0.9 (active heme = 0.8 via a CO-binding assay^35^), 0.7, and 0.8 equivalents, respectively, using measured extinction coefficients: GcoA-heme *ε*_417 nm _= 114 ± 4 mM^−1^ cm^−1^; GcoB-FAD *ε*_454 nm _= 26.6 ± 0.2 mM^−1^ cm^−1^; GcoB-2Fe-2S *ε*_423 nm _= 25.2 ± 0.1 mM^−1^ cm^−^^1^. Reduced GcoB exhibits a rhombic EPR signature with temperature saturation behavior typical for a 2Fe-2S cluster^36^.

The reductase activity of GcoB was monitored using NADH or NADPH in a cytochrome c reduction assay (Supplementary Fig. 16–20)^35^. In agreement with our comparative structural analysis (Supplementary Fig. 7), the reaction with NADH was over 50-fold faster than with NADPH (*k*_cat_ = 44 ± 1 s^−1^, *K*_M_ *=* 16 ± 0.2 μM, 25 °C, pH 7.5; all activity measurements referenced per active cofactor). Demethylation of guaiacol, which binds tightly to GcoA (*K*_D_ = 6 nM; Table 1) was then monitored over time via NADH disappearance at 340 nm under steady-state conditions (Fig. 1b). The resulting *k*_cat_ (6.8 ± 0.5 s^−1^) was approximately sixfold less than the *k*_cat_ for the GcoB reduction reaction alone, suggesting that the overall rate of the two-enzyme catalytic cycle is limited by steps involving GcoA. The value for *k*_cat_ is similar to or greater in magnitude than *k*_cat_ for other known, non-P450-type *O*-aryl-demethylases acting upon their preferred substrates, including LigM (5.8 ± 0.25 s^−1^), LigX (6.1 ± 0.2 s^−1^), or PODA (0.034 s^−1^) (Supplementary Table 4).

**Table 1:** Efficiency of GcoAB toward binding and demethylation of *O*-methyl-aromatic compounds

Compound	Compound *K*_D_ (μM)^a^	*k*_cat_ (s^−1^)^b^	*K*_M_ [*O*-methyl-aryl substrate] (mM)	*k*_cat_*/K*_M_ [*O*-methyl-aryl substrate] (mM^−1^ s^−1^)	Aldehyde produced per NADH consumed^c^	Demethylated product: amount produced per NADH consumed
Guaiacol	0.0060 ± 0.002^d^	6.8 ± 0.5	0.060 ± 0.01	110 ± 20	1.2 ± 0.15	1.2 ± 0.3
3-methoxycatechol	3.7 ± 0.1	2.1 ± 0.05	0.030 ± 0.003	75 ± 7	0.90 ± 0.04	0.97 ± 0.002
Anisole	1.7 ± 0.2	3.5 ± 0.2	0.043 ± 0.004	82 ± 8	1.1 ± 0.01	0.62 ± 0.1
Guaethol^e^	0.070 ± 0.03	1.4 ± 0.09	0.015 ± 0.004	100 ± 20	1.5 ± 0.14	1.4 ± 0.3
2-methylanisole	1.0 ± 0.1	4.6 ± 0.1	0.027 ± 0.003	170 ± 20	0.90 ± 0.1	0.20 ± 0.04
Vanillin^f^	37 ± 3	n/a	n/a	n/a	0.40 ± 0.04	0.20 ± 0.01
Syringol^f^	2.8 ± 0.4	n/a	n/a	n/a	0.080 ± 0.008	0.21 ± 0.002

^a^Titration conditions: 1–6 μM GcoA, 25 °C, 25 mM HEPES, 50 mM NaCl, pH 7.5

^b^Reaction conditions: NADH consumption was determined via loss of absorbance at 340 nm (*ε*_NADH_ = 6.22 mM^−1^ cm^−1^) or loss of fluorescence at 458 nm (vanillin) in reactions containing 0.2 μM GcoAB, 100 μg/mL catalase, 300 μM NADH, and 5–300 μM methoxy-aryl substrate in 25 mM HEPES, 50 mM NaCl, pH 7.5, 25 °C, air

^c^Total [aldehyde] was assessed using the colorimetric tryptophan-functionalization assay for formaldehyde or dehydrogenase assay for acetaldehyde. The [aldehyde] was ratioed to the total [NADH] consumed, as monitored by UV/vis or fluorescence quenching (vanillin reaction)

^d^Standard deviations are representative of three or more independent measurements

^e^Guaethol is de-ethylated by GcoAB, forming catechol and acetaldehyde instead of formaldehyde

^f^Vanillin and syringol are the only partial substrates/partial uncouplers of those listed. As such, the Michaelis–Menten parameters were not measured using the described NADH oxidation assay

**Fig. 4:**
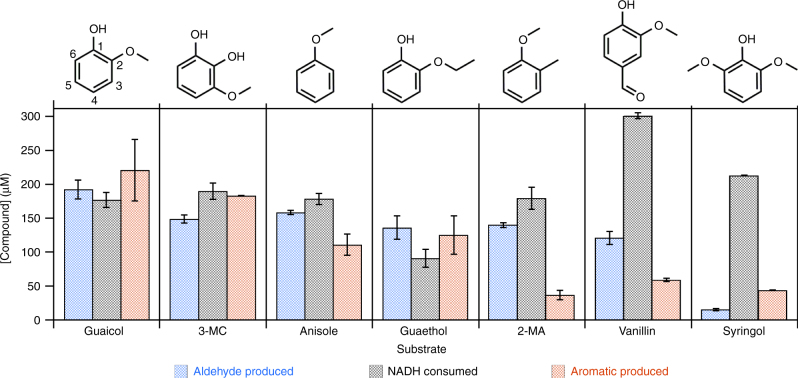
GcoAB *O*-demethylates a range of lignin-derived substrates. A total of 300 µM NADH and *O*-methyl-aryl compounds were incubated in air with 0.2 μM GcoA and B (each) for 6.75 min prior to quenching the reaction with saturated ammonium sulfate and 7% v/v concentrated H_2_SO_4_ (50 mM HEPES, pH 7.4, 25 °C). The products were then analyzed. The total NADH consumed is compared above to the amounts of aldehyde and de-alkylated aromatic compound produced. Error bars represent ±1 standard deviation from three or more independent measurements

Ten structurally diverse *O*-methylated aromatic compounds were screened for activity with GcoA. Seven of these induced measurable NADH consumption (Fig. 4) and *K*_D_ values spanning 70 nM to 37 μM (Table 1). All nonetheless yielded *K*_M_(*O*-methyl-aryl) values comparable to *K*_M_(guaiacol), indicating that all form catalytically productive complexes with GcoA. Only three (vanillate, ferulate, and veratrole) had no detectable binding or catalytic interaction with GcoA.

We next examined whether NADH consumption was coupled to substrate demethylation. Aldehyde and the demethylated aromatic products were quantified and compared to the total concentration of NADH consumed following quenching of a reaction containing saturating amounts of each substrate (Table 1, Supplementary Fig. 19). For all substrates tested except guaethol, in which acetaldehyde is produced, formaldehyde was the expected product. Five of the seven substrates that produced aldehyde (guaiacol, guaethol, 3-methoxy-catechol, anisole, and 2-methyl-anisole) did so in a ~1:1 ratio with NADH consumed (Fig. 4). Syringol and vanillin stimulated NADH turnover but produced less than stoichiometric amounts of formaldehyde. We hypothesized that these two substrates bind in the active site in the same location as guaiacol, displacing water and stimulating the spin-state change that permits reduction of the heme iron^22^. However, the reaction with O_2_ is uncoupled to substrate demethylation in some proportion of turnovers, likely leading to H_2_O_2_ release. For all 7 substrates, the expected hydroxylated-aryl product was detected and its identity confirmed by matching its HPLC retention time with known standards. However, we observed variable stability of these hydroxylated compounds in air; hence, the quantity of aldehyde product is likely a better indicator of the extent to which NADH consumption is coupled to aldehyde production.

Examining the structures of the 10 tested substrates in light of the crystallographic data suggests an emerging structure-activity relationship (Supplementary Fig. 5). First, the efficiency of guaethol as a substrate shows that the enzyme is capable not only of accommodating the larger ethoxy group (Fig. 2d) but also of catalyzing de-ethylation. Second, there appears to be a varying degree of flexibility in the permissible substituents around the aryl ring. C1 (guaiacol numbering, Fig. 4) can accommodate -OH (guaiacol, guaethol, 3-methoxy-catechol), -CH_3_ (2-methyl-anisole), or -H (anisole) with comparable efficiency constants [*k*_cat_/*K*_M_(*O*-methyl-aryl)]. However, veratrole, which has -OCH_3_ at this position, is a non-substrate/non-binder, suggesting that steric constraints supplied by the nearby α-helix (Fig. 2c) might limit the size of substituents here. Similarly, the carbon *ortho* to the -OH of guaiacol can have either -H or -OH (3-methoxy-catechol) substituents without substantial penalty to the efficiency constant, though -OCH_3_ (syringol) again leads to a partial substrate, partial uncoupler. Finally, substituting the -H at the C4 guaiacol position, which is closest to the side chain of Thr296, with a formyl group (vanillin) likewise yields a partial substrate, partial uncoupler; however, carboxylic acid (vanillate and ferulate) substituents preclude binding and/or demethylation. The partial demethylation observed for syringol and vanillin, despite the fact that both assume a guaiacol-like binding mode in the crystal structures (Fig. 2), suggests that dynamic factors may be important for understanding the efficiency of the GcoA-substrate interaction.

### Enzyme opening and closing are key steps in GcoA catalysis

**Fig. 5:**
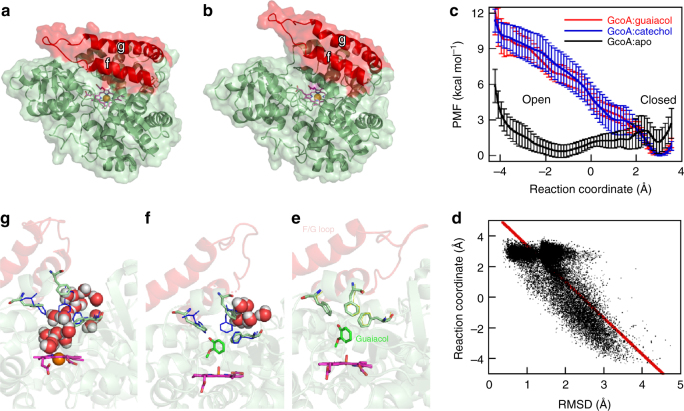
MD simulations of GcoA demonstrate opening and closing of the active site correlated with Phe side chain motions and substrate binding. **a** Closed and **b** open GcoA structures obtained from MD simulations highlighting in red the open-close motion of the helices F and G. **c** Free energy profiles (calculated as the potential of mean force—PMF) of the open-close motions of GcoA:apo, GcoA:guaiacol, and GcoA:catechol. PMFs were calculated using 10-ns blocks from the umbrella sampling simulations and the block averages were plotted with the corresponding standard deviations represented as error bars. **d** Scatter plot of the reaction coordinate (a metric of the open-close motions) and RMSD of Phe75, Phe169, and Phe395 relative to the crystal structure (a metric of the breathing motions of these residues) obtained from simulations of GcoA:apo, showing the correlation between the degree of opening of GcoA and the configuration of the binding pocket. Configuration of residues Phe75, Phe169, and Phe395 (in cyan) **e** in the most closed GcoA structure, **f** in a partially open GcoA structure, and **g** in a fully open structure, showing that the aromatics (from left to right; Phe75, Phe169, Phe395) prevent water (shown as space filling representation) from penetrating the binding pocket in **f** but not in **g**, where the expansion of the Phe residues allows the penetration of water into the binding pocket. For visual clarity, the water molecules represented in **g** and **f** correspond to those located within 7 Å of the Phe side chains and within 16 Å of the heme Fe atom. The configuration of the Phe residues in the crystal structure is shown as thin blue lines

Some P450 enzymes are known to undergo conformational opening and closing motions during their catalytic cycles^37^. While the structures of GcoA obtained in this study were crystallized in an apparent closed state, molecular dynamics (MD) simulations indicated that an in silico-generated apo form of GcoA can spontaneously open on the time scale of 1 μs, with the structural changes occurring mainly in the F and G helices (Fig. 5a, b, Supplementary Fig. 21 and Supplementary Movies 1-2). The overall conformation of the MD-generated GcoA open structure closely resembles that of the well-characterized open form of the P450 from *Bacillus megaterium* BM3, (PDB ID: 2HPD)^38^ (Supplementary Fig. 22).

We subsequently employed umbrella sampling (US) to obtain the free energy profile for the opening-closing motion of the enzyme in the apo, guaiacol (substrate), and catechol (product) forms. The free energy was computed along a reaction coordinate defined as the difference between the root mean square deviation (RMSD) from the open structure and the RMSD from the closed structure. The free energy profile of GcoA:apo is relatively flat and exhibits two minima, one associated with the open state and another with the closed state (Fig. 5c). The free energy of the closed state is, within error, equivalent to that of the open state, with a free energy barrier <4.5 kcal/mol. This indicates that GcoA can easily transition between the open and closed states when a substrate is absent, which may help to explain the difficulty in obtaining a crystal structure of the apo-form. In contrast, the free energy profile associated with the opening-closing motion of either GcoA:guaiacol or GcoA:catechol exhibits only one minimum, associated with the closed state, suggesting that the enzyme will close when the substrate or product is bound. We note, however, that the free energy cost for GcoA:guaiacol or GcoA:catechol to visit a partially open state transiently is about 5 ± 1 kcal/mol (at a reaction coordinate value of 0), indicating that this event is likely. Indeed, MD simulations show that GcoA opens when guaiacol or catechol is present, but only partially, and returns back to the closed state (Supplementary Fig. 21). Together, these results suggest that the substrate binds to the open state of GcoA and induces motions necessary to close the enzyme. Transient partially open states would enable the catechol product to leave the enzyme.

Local changes in the active site are closely related to the opening-closing motions of GcoA. Simulations show that residues Phe75, Phe169, and Phe395, which surround the aryl ring of the substrate in the active site as seen in Fig. 2c, undergo breathing motions as a function of the GcoA opening-closing movement. Larger deviations in the positions of the Phe residues are associated with open states of GcoA, as seen in Fig. 5d, which shows the correlation between the open-closed motions in GcoA:apo, as measured by the reaction coordinate computed along simulations, and breathing motions of residues Phe75, Phe169, and Phe395 (Fig. 5e-g), measured by their RMSD relative to the crystal structure (details of the Phe aromatic center distances are presented in Supplementary Fig. 23). The presence of guaiacol or catechol reduces the breathing motions of the Phe residues (Supplementary Fig. 21), as their RMSD never reaches values >3 Å, in contrast to the RMSD in GcoA:apo, which can reach values greater than 4 Å. In the configuration shown in Fig. 5e, which occurs in the crystal structures and corresponds to an RMSD <2 Å, the three Phe residues are close to each other and interact directly with guaiacol. This configuration tends to occur when GcoA is closed in the simulations (Fig. 5d, Supplementary Fig. 21). In the configuration in Fig. 5f, which corresponds to the 2-3 Å RMSD range and occurs when GcoA is partially open (with the reaction coordinate around 0), the side chain of Phe169 deviates from its crystallographic position, but the three Phe residues still interact with the substrate and exclude water from the active site. In the configuration in Fig. 5g, which shows the most open state of GcoA:apo (when the reaction coordinate assumes values less than 0 and at an RMSD value >3 Å), the Phe side chains move even further apart from each other, expanding the binding site and allowing water to enter the enzyme. Overall, the presence of guaiacol or catechol in the binding site keeps the three Phe residues arranged around the ligand and prevents the full closed-to-open transition of the active site.

Simulations of GcoA:guaethol, GcoA:syringol, and GcoA:vanillin indicate different effects of these substrates on the conformational flexibility of GcoA (Supplementary Fig. 21) and help explain the structure-activity relationships in this enzyme. GcoA:guaethol exhibits less flexibility than GcoA:guaiacol, as observed by the limited opening-closing motions and binding site expansion. Given an almost fivefold reduction in *k*_cat_ for guaethol compared to guaiacol, it appears that such flexibility is required for optimal substrate turnover. GcoA:syringol and GcoA:vanillin, on the other hand, are both more flexible than GcoA:guaiacol and more prone to opening-closing transitions and expansion of the active site. This indicates that syringol and vanillin, which bind to the active site, stimulate NADH turnover, but are not stoichiometrically demethylated (Table 1), are less effective in maintaining the enzyme in the closed state than guaiacol and guaethol. This suggests that successful protein engineering for alternative substrates will require careful consideration to balance conformational flexibility with productive binding and catalysis, and these data provide a route to help define the optimum window.

### Proposed reaction mechanism for guaiacol *O*-demethylation

**Fig. 6:**
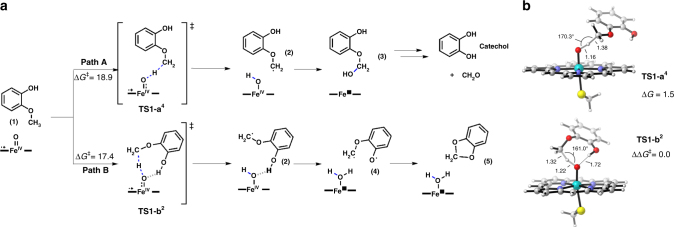
Proposed GcoA-catalyzed reaction mechanisms for the degradation of guaiacol. **a** Schematic representation of the two potential reaction paths catalyzed by GcoA P450. Path A generates the hemiacetal (3), which will hydrolyze into the observed catechol and formaldehyde. Path B could generate a stable and unproductive acetal (5). **b** Path A and B DFT optimized H-abstraction rate-limiting transition states. Gibbs energies are given in kcal mol^−1^, distances in Å, and angles in degrees

Having identified that GcoA contributes the rate limiting step in this 2-protein system, density functional theory (DFT) calculations were used to investigate the mechanism for guaiacol *O*-demethylation. The putative enzymatic reaction is shown (Fig. 6) with guaiacol as the modeled substrate. DFT calculations using a truncated model system identified two possible reaction pathways (path A and path B) that GcoA could catalyze, which rely to two different approaches of the guaiacol substrate to the Fe = O active species (Fig. 6) (see Supplementary Table 5 and Supplementary Data 1 for DFT energies and optimized geometries).

Path A leads to the formation of hemiacetal (3) through a hydrogen atom transfer (HAT) rate-limiting step, followed by a rapid OH rebound. Hemiacetal **3** can then degrade in solution to form the *O*-demethylated catechol product and formaldehyde. Conversely, path B would form a stable acetal **4** in two sequential HAT reactions: a rate-limiting C-H abstraction followed by a subsequent O-H abstraction to generate a biradical intermediate **4** that cyclizes in a barrierless process to form **4** (Fig. 6 and Supplementary Figs. 24–26).

The main difference between the two rate-limiting transition states (TSs) is the conformation that the guaiacol substrate adopts with respect to the Fe = O active species. **TS1-a**^**4**^ corresponds to the HAT transition state when the substrate/Fe = O orientation is similar to the one observed in the substrate-bound crystal structure (see Figs. 2c and 6b); **TS1-b**^**2**^ is the lower energy HAT TS (1.5 kcal/mol lower than **TS1-a**^**4**^). In this case, the substrate orientation allows the guaiacol hydroxyl group to interact by H-bond with the Fe = O, stabilizing the TS but also permitting the second OH H-abstraction (Fig. 6). The direct comparison of the two rate-limiting TSs proved the intrinsic preference of the substrate to react following path B over path A. Nevertheless, the strong preference of the substrate to bind in the specific orientation found in the crystal structure as observed during the course of the MD simulations (Fig. 5f and Supplementary Fig. 27), indicates that path A will be followed although it is energetically less favorable.

Alternatively, open-shell singlet biradical intermediate **4** in path B could form the less stable zwitterionic closed-shell electronic configuration (1.6 kcal/mol higher in energy than the biradical) and further react with a water molecule to generate the hemiacetal **3**. The absence of water molecules in the active site environment when guaiacol is bound as observed from our MD simulations (Supplementary Fig. 28) argues against this possibility.

## Discussion

Recent efforts from multiple groups have attempted to harness aromatic catabolism for productively utilizing lignin^8–10,18,39–44^. As a single microbe is unlikely to have the full complement of necessary catabolic enzymes for lignin bioconversion, a key component of such synthetic biology strategies is the introduction of foreign catabolic genes to expand substrate specificities of the host microbe. Bacterial enzymes that catalyze the demethylation of lignin-derived aryl-methoxy substrates are of particular interest, as the demethylation reaction presents a bottleneck for the conversion of lignin into desirable products. Currently, Rieske non-heme iron monooxygenases^14,15,18^ and tetrahydrofolate-dependent *O*-demethylases^16,17^, offer two well-known paradigms for aryl-*O*-demethylation. This study presents a detailed characterization of a third, cytochrome P450-based enzymatic strategy that could fill a critical gap for engineering applications.

From a metabolic engineering standpoint, the GcoAB system offers a number of potential advantages. First, the native substrate of GcoA, guaiacol, is a major breakdown product of plant lignin. Demethylation of guaiacol yields catechol, which can be ring-opened via either intra- or extra-diol cleavage catechol dioxygenases. Second, compared to other known *O-*aryl-demethylases, the substrate preferences of GcoA are intrinsically broad, admitting a variety of guaiacol analogs that are also known lignin breakdown products. Third, we anticipate a P450 system to be amenable to further tuning using directed evolution techniques^45^. A prior report of a closely related cytochrome P450 that can demethylate 4-methoxybenzoate^12^ suggests that the GcoA active site may be modified to admit larger, more hydrophilic, lignin-derived substrates such as ferulate or vanillate. Indeed, genes encoding putative homologs to the two-component GcoA and GcoB system described here are predicted in the genomes of several bacterial species belonging to the genera *Rhodococcus*, *Streptomyces*, and *Gordonia*, among others, and the substrate preferences for this diverse group remain unclear, but offer a promising platform for further exploration and engineering. Moreover, work from Bell et al. revealed an unrelated Rhodopseudomonad cytochrome P450 can also demethylate 4-methoxybenzoate and be productively engineered to accommodate 4-ethylbenzoate. While retaining a classical P450 fold, this CYP199A4 system exhibits an alternative binding mode in terms of both substrate positioning relative to the heme, and steric selectivity with an alternative set of aromatic residues lining the active site pocket, further demonstrating the diversity within this class of enzymes. Fourth, a heme-based P450 may offer a simpler alternative for aromatic demethylation compared to tetrahydrofolate-dependent *O*-demethylases, given the relative ubiquity of P450s and robust heme biosynthetic pathways in potential bacterial hosts. Finally, distinct from most P450 systems, the GcoB reductase is encoded as a single polypeptide rather than two.

Close examination of the GcoA-guaiacol active site shows that substrate binding involves interactions between the peptide backbone and the substrate hydroxyl (ring C1) and methoxy groups (C2). The ring C3 position has a relatively close (3.8 Å) interaction with the porphyrin-γ*-meso* carbon that bridges the propionate-substituted pyrrole rings. However, the remaining ring positions are not directly occluded by backbone/porphyrin atoms. Reactivity studies showed that guaiacol analogs with substitutions at C4, C5, and C6 remained substrates with comparable efficiencies (*k*_cat_/*K*_M_) to guaiacol itself. Even substitutions at C1 and C2 (ethoxy- for methoxy-) are permitted. Comparison of the structures of the guaiacol, guaethol, vanillin, and syringol ligand-bound GcoA structures shows that all assume a similar binding mode with only subtle reorganization of the surrounding active site residues.

MD simulations suggest that the active site opens and closes in response to substrate binding. This flexibility in the active site, in which several side chains (e.g., Phe75, Phe169, and Phe395) reorganize to accommodate the bound ligand, may be partly responsible for the observed substrate promiscuity of GcoA. Though the active site is flexible, potential substrates must be able to maintain the closed state of the active site in order to prevent the uncoupling of NADH oxidation from substrate hydroxylation. The same simulations also suggest that the active site constrains the binding mode of guaiacol, so that the methoxy group points toward and the hydroxyl group is oriented away from the heme iron. This may forestall the lower-energy cyclization reaction pathway predicted for guaiacol by DFT calculations.

Together, the structural, biochemical, and computational data presented here suggest a GcoA active site that is sufficiently accommodating to turnover a range of substrates that each react in the desired fashion to release an aldehyde product. We hypothesize that the substrate range and consequently the utility of GcoA may be extended even further, to accommodate important G- and S-type lignin subunits, by protein engineering or directed evolution. Tests of this hypothesis are the subject of ongoing work.

## Methods

### Materials

Standards of 2-methylanisole, 2-methylphenol, 3-methoxycatechol, anisole, caffeic acid, catechol, 3,4-dihydroxybenzaldehyde, ferulic acid, guaethol, guaiacol, phenol, protocatechuic acid, pyrogallol, syringol, vanillic acid, vanillin, and veratrole were purchased from Sigma Aldrich (Sigma Aldrich, St Louis, MO).

### Protein expression and purification

The genes for *gcoA* and *gcoB* were amplified from *Amycolatopsis* sp. ATCC 39116 genomic DNA and assembled separately using NEBuilder® HiFi DNA Assembly Master Mix (New England Biolabs) into the pGEX-6P-1 vector (GE Lifesciences), which codes for an N-terminal glutathione-S-transferase (GST) fusion tag. Oligonucleotide primer sequences are provided in Supplementary Table 1. Expression constructs were expressed in *E. coli* Rosetta™ 2 (DE3) cells (Novagen). Cells were transformed with plasmids containing either the GcoA or GcoB fusion construct and plated out on lysogeny broth (LB) agar containing chloramphenicol (34 mg/L) and carbenicillin (50 mg/L). A single colony was selected and used to inoculate a 20 mL starter culture of LB. After overnight growth at 37 °C, 250 rpm, the starter culture was inoculated into 2.5 L flasks containing 1 L of either terrific broth (TB) (GcoA) or LB (GcoB) with antibiotics. At an OD_600_ of 0.5 (GcoB) or 1.0 (GcoA), 0.2 mM IPTG was added to induce protein expression. Additionally, 100 mg/L 5-aminolevulinic acid (GcoA), or 200 mg/L ammonium iron(III) citrate (GcoB) was added to support productive cofactor incorporation. Induction of protein expression was performed for 16–18 h at 20 °C with shaking at 250 rpm. Affinity purification was carried out using glutathione-sepharose 4B media (GE Lifesciences) followed by GST-tag cleavage with PreScission protease (GE Lifesciences). Anion exchange chromatography was performed using a MonoQ 5/50 GL column (GE Lifesciences) for GcoB with a 0–50% gradient of 50 mM HEPES, pH 7.5, 1 M NaCl, 1 mM DTT, and with a Source 30Q column (GE Lifesciences) with a 10–40% gradient of the same buffer for GcoA. For each protein, a final gel filtration step was performed using a HiLoad S200 16/60 pg column (GE Lifesciences) in a buffer containing 25 mM HEPES, pH 7.5 and 50 mM NaCl. Preparation of a GcoA SeMet derivative was achieved by expression in Selenomethionine Medium Complete (Molecular Dimensions) according to the manufacturers protocol and purification was performed using the same method as used for the native GcoA protein.

### UV/vis spectroscopy of GcoA and GcoB

The spectra of GcoA and GcoB were measured in 25 mM HEPES, 50 mM NaCl at pH 7.5 using a Lambda 25 spectrophotometer (Perkin Elmer) over a wavelength range of 200–600 nm at 1 nm intervals in a quartz cuvette (Hellma Analytics).

### Heme quantification

Catalytically active heme bound to GcoA was determined via a spectrophotometric/CO-binding assay^35^. CO gas was bubbled into a cuvette containing 0.94–2.5 μM GcoA (Pierce BCA assay). Excess sodium dithionite (~1 mg) was added to reduce the heme iron. The spectrum was recorded over a period of several minutes as a peak centered at ~450 nm gradually appeared, attributed to the catalytically competent, ferrous CO-bound heme. A spectrum for a control containing only dithionite-reduced GcoA was measured, and a difference spectrum computed. Absorbances at 420, 450, and 490 nm were recorded to calculate the amount of active GcoA (P450) or inactive GcoA (P420 nm). The equations used to compute the concentrations of catalytically competent and inactive heme^35^ are shown below. Reported values are the average of three or more measurements.

1 $$\left( {\Delta { A}450 - \Delta { A}490} \right)/0.091 = {\rm nmol}\,{\rm of}\,{\rm P}450\,{\rm per}\,{\rm mL},$$

2 $$\begin{array}{l}\left[ {\left( {\Delta { A}420 - { A}490} \right)_{{\rm observed}} - \left( {{ A}450 - { A}490} \right)_{{\rm theoretical}}} \right]/0.110\\ = {\rm nmol}\,{\rm of}\,{\rm cytochrome}\,{\rm P}420\,{\rm per}\,{\rm mL}\end{array},$$

3 $${\rm nmol}\,{\rm of}\,{\rm P}450\,{\rm per}\,{\rm mL}\,x\left( { - 0.041} \right) = \left( {\Delta { A}420 - { A}490} \right)_{{\rm theoretical}}.$$

Here Δ*A*450 and Δ*A*420 are the differences between the reference and sample spectra at absorbances 450 and 420 nm, respectively.

The pyridine hemochrome assay was additionally used to assess the total heme content in GcoA^46^. Volume of 200 µL of a 6 µM GcoA solution was added to 797 µL 50 mM NaOH, 20% pyridine and 3 µL K_3_Fe(CN)_6_ and a UV/vis spectrum measured. Excess (2–5 mg) sodium dithionite was used to reduce the heme and the absorbance at 556 nm was compared to the oxidized spectrum (Δ*A*). The Beer-Lambert law was used to calculate the amount of heme present, using *ε*_556_ = 28.4 mM^−1^ cm^−1^. Reported values are averaged from three or more measurements. An extinction coefficient for GcoA-bound heme, using the [GcoA-heme] determined from the CO binding assay, was estimated via the slope of a line relating absorbance at 417 nm (Soret peak) to [GcoA-heme].

### Determination of [FAD] and non-heme [Fe] in GcoB

FAD was released from GcoB by denaturing 200 μL of a protein (0.024 µM) solution with 5 μL saturated ammonium sulfate, pH 1.4 (7% v/v H_2_SO_4_), similar to studies with related cytochrome P450s^47^. Precipitated protein was pelleted by centrifugation and the UV/vis spectrum of the FAD-containing supernatant was measured. The absorbance at 454 nm, *ε*_FAD _= 11.3 mM^−1^ cm^−1^, and total protein concentration determined by the Bradford assay were used to determine [FAD] bound to GcoB. An extinction coefficient for GcoB-bound FAD was estimated via the slope of a line relating absorbance at 454 nm to [GcoB-FAD].

The Fe–S content of GcoB was assessed both by quantifying non-heme Fe(II)^48^ and spectroscopically characterizing the cluster as a whole (below). GcoB was denatured as described above. Volume of 50 μL of supernatant was added to 25 μL of 5% w/v sodium ascorbate to reduce the iron. Volume of 100 μL of bathophenanthroline disulfonate (0.1% w/v in ddH_2_O) was added and the sample was incubated for 1 h. The resulting Fe(II) complex was quantified via its absorbance at 535 nm (*ε*_535_ = 22.14 mM^−1^ cm^−1^, determined using FeSO_4_ standards). An extinction coefficient for GcoB-bound 2Fe-2S cluster was estimated via the slope of a line relating absorbance at 423 nm to [GcoB-2Fe-2S].

### EPR and UV/vis spectroscopic characterization of FeS cluster

A 150 μM sample of GcoB was brought into an MBraun chamber and exchanged into anaerobic 50 mM Tris, 200 mM NaCl, 5% glycerol, pH 7.0. The sample was then reduced anaerobically with 10 mM sodium dithionite and loaded into an EPR tube. The tube was capped prior to removing from the chamber and frozen in liquid N_2_. X-band EPR spectra were recorded on a Bruker E-500 spectrometer equipped with a Super High Q (SHQ) resonator, in-cavity cryogen-free system (ColdEdge Technologies), and MercuryiTC temperature controller (Oxford). Spin quantifications were determined by comparison to copper standards at 75, 100, and 125 µM via double integration of the spectra after baseline subtraction using the OriginPro software package. Spectral simulation was carried out with the EasySpin software package for *g*-value determination^49^. Fe_*X*_S_*Y*_ clusters of different nuclearities have EPR spectra with distinct temperature dependencies. Based on the cluster type determined via EPR, the [Fe(II)] was found to be in close agreement to the results from the bathophenanthroline disulfonate assay described above. Thus, the colorimetric assay was used to determine all subsequent [2Fe-2S] and to find the *ε*_423 nm_.

### Reductase activity measurement of GcoB

GcoB activity was assessed using a continuous colorimetric assay involving cytochrome c as a colorimetric electron acceptor^35^. A total of 4.8 nM GcoB (referenced to [FAD]) and 42 µM cytochrome c (from equine heart) were dissolved in buffer (25 mM HEPES, 50 mM NaCl, pH 7.5), 25 °C. 100 µM NADH was then added to initiate the NADH- and GcoB-dependent reduction of cytochrome c. UV/vis spectra (Varian Cary 4000, Agilent) were recorded in the scanning kinetics mode. The increase in absorbance at 550 nm due to reduced cytochrome c was monitored over time, and the specific activity (nmol reduced cytochrome c min^−1^ nmol GcoB^−1^) calculated using:^35^

4 $$\Delta { A}_{550}{\rm min}^{ - 1}/0.021/{\rm mL}\,{\rm reaction} = {\rm specific}\,{\rm activity}.$$

For determining steady-state kinetic constants, the above protocol was used as a function of [NADH]. A total of 4.8 nM GcoB (referenced to [FAD]) and 43 µM cytochrome c were dissolved in buffer (25 mM HEPES, 50 mM NaCl, pH 7.5), 25 °C, and the reaction was initiated with the addition of 2.5–200 µM NADH. Initial velocities (*v*_*i*_) were determined from linear fits to the initial portion of the progress of reaction data, plotted as a function of [NADH], and fit to the Michaelis–Menten Eq. (5) using the KaleidaGraph software:

5 $$v_i = V_{{\rm max}}\left[ {\it{S}} \right]/\left( {K_{\mathrm M} + \left[ S \right]} \right).$$

### Steady-state kinetics analysis of GcoAB

The demethylation of guaiacol and substrate analogs was continuously monitored under steady-state conditions. A total of 0.2 µM each of GcoA and GcoB were dissolved in air-saturated buffer (25 mM HEPES, 50 mM NaCl) in a cuvette at pH 7.5, 25 °C. A total of 100 µg/mL catalase was added to each reaction to capture any H_2_O_2_ formed during the uncoupled reaction. A saturating amount of NADH (≥5*K*_M_, 300 µM) was added and a background rate of NADH oxidation in air (>200 µM O_2_) recorded via continuous scanning of the UV/vis spectrum. A total of 20–300 µM guaiacol (preferred substrate) or an alternate substrate from a 2.5 mM stock dissolved in DMSO was added and the reaction was monitored via measurement of UV/vis spectra for several minutes. The initial velocity was determined by disappearance of the characteristic NADH absorbance at 340 nm (*ε*_344_ = 6.22 mM^−1^ cm^−1^). A plot of *v*_*i*_ vs [guaiacol] was fit to Eq. (2) to obtain *k*_cat_*, K*_M_, and *k*_cat_*/K*_M_. For specific activity determination, the above method was used but with saturating (300 µM) guaiacol. The linear portion of [NADH] vs time was fit and referenced to the amount of GcoA used (0.2 µM). Reported values are the average of ≥3 measurements and reported errors are standard deviations.

For vanillin, whose UV/vis spectrum overlaps with that of NADH, fluorescence was used to monitor NADH disappearance using a FluoroMax3 instrument (Horiba Jobin Yvon). A standard curve for NADH (0–350 µM) was generated by exciting the sample (25 mM HEPES, 50 mM NaCl pH 7.5) at 340 nm and monitoring the emission at 458 nm (vanillin did not excite or emit at this wavelength). The intensity vs [NADH] was plotted and fit to a 4-parameter logistic equation:

6 $${\rm Intensity} = \frac{{a - d}}{{1 + \left( {\frac{x}{c}} \right)^b}} + d,$$

where *a* is the theoretical response at [NADH] = 0, *b* is the slope of the curve at the inflection point, *c* is [NADH] at the inflection point, and *d* is the theoretical response at infinite [NADH]. Reactions with vanillin were performed in the same manner described above.

### Determination of substrate dissociation constants with GcoA

A total of 0–60 µM of substrate analogs, in 0.25 or 0.5 µM aliquots, were titrated into a cuvette containing 1–6 µM GcoA in 25 mM HEPES, 50 mM NaCl, pH 7.5. The spectrum after each substrate addition was recorded, beginning with no substrate bound. The solution reached equilibrium before the next addition. A difference spectrum was made to illustrate the shift from a low-spin aquo-heme complex to the high-spin substrate-bound complex (spectral shift from 417 nm to 388 nm). The resulting difference spectra showed a peak at 388 nm, and a trough at 420 nm. The absorbance at 388 nm was plotted as a function of [substrate], yielding a quadratic curve that was fit to Eq. (7) to determine the *K*_D_.

7 $${\mathrm{\Delta }}{\rm Abs}_{{\rm obs}} = \frac{{{\mathrm{\Delta }}{\rm Abs}_{{\rm max}}}}{{2E_t}}\left( {L_0 + E_t + K_{\rm D} - \sqrt {\left( {L_0 + E_t + K_{\mathrm D}} \right)^2 - 4E_t \ast L_0} } \right).$$

Where *L*_0_, *E*_*t*_, *K*_D_, and ΔAbs_max_ are the ligand concentrations, total protein (subunit) concentration, the equilibrium dissociation constant, and the maximum Abs_388–417 nm_, respectively. Reported values are the average of 2 or more measurements.

Since the UV/vis spectra of vanillin and heme overlap, fluorescence quenching was monitored to determine the *K*_D_. 4 µM GcoA (25 mM HEPES, 50 mM NaCl, pH 7.5) was excited at 283 nm and the emission peak read at 340 nm; vanillin does not show the same excitation/emission pattern. Fluorescence emission intensity was followed upon titration of vanillin (0–350 µM) after equilibrium had been established. The % fluorescence quenched, Eq. (8), was plotted as a function of vanillin and fit to Eq. (7) above, replacing ΔAbs_max_ with ΔFluorescence_max_.

8 $${\mathrm{\% }}F\,{\rm quenched} = \frac{{F_{{\rm max}} - F_{{\rm intensity}}}}{{F_{{\rm max}}}}\times100{\mathrm{\% }}.$$

### Aldehyde product determination

For the quantification of formaldehyde (demethylation) or acetaldehyde (de-ethylation) production, the reaction was monitored via UV/vis or fluorescence (in the case of vanillin) using the same set of conditions as outlined above for specific activity determination. After eight minutes, aliquots of the sample were removed and carried onto the respective aldehyde detection assay. For reactions that produced formaldehyde (e.g., all substrates except guaethol), a colorimetric tryptophan assay was used, described previously^50^. Briefly, 200 µL of the reaction was quenched by adding 200 µL of 0.1% tryptophan solution in 50% ethanol, 200 µL 90% sulfuric acid and 40 µL of 1% FeCl_3_. The solution was then incubated in a heating block for 90 min at 70 °C. After cooling, the absorbance was read at 575 nm and the [formaldehyde] calculated by comparing to a standard curve made with 0–320 µM formaldehyde. A reaction with just the assay components was treated in the same manner as the reaction samples and used as a baseline. Control reactions included everything but GcoA/B, catalase, and substrate. Reported values are the average of ≥3 measurements.

[Acetaldehyde] produced during the dealkylation of guaethol was also determined by using a colorimetric assay and a generated acetaldehyde standard curve. A kit from BioAssays was used. Briefly, 20 µL of the reacted sample was transferred to a 96-well plate and 80 µL of the working reagent, consisting of NAD/MTT and aldehyde dehydrogenase, was added. The reaction was incubated for 30 min at room temperature and the absorbance read at 565 nm. Aldehyde dehydrogenase and NAD react with acetaldehyde to produce acetic acid and NADH. The NADH can then reduce MTT, resulting in the absorbance at 565 nm. Control reactions included samples without substrate and/or aldehyde dehydrogenase. Reported values are the average of ≥3 measurements.

### HPLC product identification

Analyte analysis of samples was performed on an Agilent 1200 LC system (Agilent Technologies, Santa Clara, CA) equipped with a G1315A diode array detector (DAD). Each sample and standard was injected at a volume of 10 μL onto a Phenomenex Luna C18(2) column 5 μm, 4.6 × 150 mm column (Phenomenex, Torrance, CA). The column temperature was maintained at 30 °C and the buffers used to separate the analytes of interest was 0.05% acetic acid in water (A)/ acetonitrile (B). The separation was carried out using a gradient program of: (A) = 99% and (B) = 1% at time *t* = 0; (A) = 99% and (B) = 1% at time *t* = 5, (A) = 50% and (B) = 50% at *t* = 35 min; (A) = 1% and (B) = 99% at *t* = 35.01 min; (A) = 99% and (B) = 1% at *t* = 37.01 min; (A) = 99% and (B) = 1% at *t* = 47.00 min. The flow rate was held constant at 0.6 mL min^−1^ resulting in a run time of 47 min. Calibration curve concentration for each analyte varied between the ranges of 2.5–200 µg L^−1^. A DAD wavelength of 225 nm was used for analysis of the analytes of interest. In addition, 210 nm was used for protocatechuic, 4-hydroxybenzoic acid, guaiacol, syringol, veratrole, guaethol, vanillin, vanillic acid, catechol, 3-methoxycatechol, anisole, 2-methylanisole, dihydroxybenzaldehyde, phenol, and 2-methylphenol and 325 nm was used for p-coumaric acid, ferulic acid, caffeic acid, and pyrogallol. A minimum of 3–5 calibration levels was used with an *r*^2^ coefficient of 0.995 or better for each analyte and a check calibration standard (CCS) was analyzed every 10 samples to insure the integrity of the initial calibration.

### Crystallization and structure determination

Purified protein was buffer exchanged into 10 mM HEPES pH 7.5 and concentrated to *A*_280_ values of 12 and 5 for GcoA and GcoB, respectively, as measured on a NanoDrop 2000 spectrophotometer (Thermo Fisher). Crystals of GcoA were grown with 1.8 M sodium malonate and 20 mM substrate. GcoB crystals were grown in the Morpheus Screen 0.09 M halogens mix (0.3 M sodium fluoride; 0.3 M sodium bromide; 0.3 M sodium iodide), 0.1 M buffer system 3 (1 M Tris (base); BICINE pH 8.5) and 60% v/v precipitant mix 1 (40% v/v PEG 500 MME; 20% w/v PEG 20000) (Molecular dimensions). Crystals in both crystallization conditions were successfully cryocooled directly in liquid N_2_ without further addition of cryoprotectants. All data were collected at Diamond Light Source (Harwell, UK). The complex of GcoA with guaiacol was collected on I04 and the phases were solved with a single SAD dataset at a wavelength corresponding to the Se edge. Data from crystals of GcoA with guaethol, syringol, and vanillin were collected on I03 and the data from GcoB crystals were collected on I04. Data were processed with using the CCP4^51^ and Phenix^52^ suites and details are provided in Supplementary Methods. Data collection and refinement statistics can be found in Supplementary Table 2. Structural images were generated using PyMOL ((http://www.pymol.org) and surface charge calculated using DelPhi^53^.

### Dynamic light scattering

Dynamic light scattering experiments were performed using a Protein Solutions DynaPro MSTC800 instrument operated through the Dynamics version 5.26.60 software package (Protein Solutions). Samples were passed through a 0.1 μm filter prior to measurement at 20 °C. Results were taken from at least 20 measurements and the data were analyzed using the Dynamics software. The molecular weights of the proteins were estimated using the empirical equation for a globular protein:

9 $$M_r = \left( {1.68 \times R_h} \right)^{2.34},$$

where *M*_*r*_ = the molar mass of the protein in kilodaltons and *R*_*h*_ = the hydrodynamic radius of the protein in nm.

### Analytical ultracentrifugation

Velocity analytical ultracentrifugation was performed using a Beckman XL-A analytical ultracentrifuge with an An50-Ti rotor. Double-chamber Epon cells were used with 1.2 cm path lengths and quartz window assemblies. Protein concentration was measured at 37.5 μM with a 1:0.9 ratio of GcoA:GcoB for the GcoAB run in buffer containing 25 mM HEPES pH 7.5 and 50 mM NaCl. Samples were equilibrated at 20 °C at 3000 rpm before accelerating to 40,000 rpm and taking 72 radial scans at 20 min intervals at a wavelength of 280 nm. Sednterp^54^ was used to calculate buffer viscosity and density, $\bar v$ and of the protein sample and Sedfit^55^ was used to analyze the scans, solve the Lamm equation, perform *c*(*s*) size-distribution analysis and determine *f*/*f*_0_.

### MD simulation and DFT systems setup

Molecular dynamics simulations were performed with GcoA in the following conditions: complexed to guaiacol (GcoA:guaiacol), guaethol (GcoA:guaethol), syringol (Gcoa:syringol), vanillin (GcoA:vanillin), catechol (GcoA:catechol), and in absence of ligand (GcoA:apo). For GcoA:guaiacol, GcoA:guaethol, GcoA:syringol and GcoA:vanillin, we used the crystal structures reported in this work as starting point. GcoA:catechol and GcoA:apo systems were built from the GcoA:guaiacol structure by modifying and removing the guaiacol substrate, respectively. The heme group was considered in the pentacoordinate state in the GcoA:apo and GcoA:catechol systems, and in the hexacoordinate (compound I) state in the GcoA:guaiacol, GcoA:guaethol, GcoA:syringol and GcoA:vanillin systems. As the GcoA:vanillin crystal structure lacks residues 388 and 389, these were taken from the GcoA:syringol structure after structural alignment. Hydrogen atoms were added and the protonation states of titratable residues were estimated using H++ at pH 7.5^56,57^. At this condition, all Asp and Glu residues were considered deprotonated (bearing a -1 charge); His131, His221, His224, His255, and His343 were considered doubly protonated (+1 charge); and His80, His91, His213, His329, His349, His357, His367, and His405 were considered protonated only at the *ε* position (0 charge). The systems were then immersed in a rectangular water box extended at least 15 Å from the protein, and Na^+^ cations were added to neutralize the system. The final systems comprised ~74,000 atoms.

Force field parameters for guaiacol, guaethol, syringol and vanillin were obtained from Generalized Amber Force Field (GAFF)^58^, with Restrained Electrostatic Potential (RESP) partial charges^59^ derived at the HF/6-31 G(*) level with Gaussian 09^60^. Force field parameters for the heme group, both in the pentacoordinate state and compound I were taken from from Shahrokh et al.^61^. For the protein, the ff14SB Amber force field^62^ was employed along with the TIP3P water model^63^. The simulations were performed using periodic boundary conditions, with short-range interactions truncated at a cutoff radius of 8.0 Å and particle mesh Ewald (PME) for long-range interactions^64^. The equations of motion were integrated with a time-step of 2.0 fs, with bonds involving hydrogen atoms constrained at their equilibrium values using SHAKE. The temperature was kept constant at 300 K using the Langevin thermostat with a collision frequency of 1.0 ps^−1^. The pressure was controlled at 1.0 bar only during the initial equilibration steps (described below) with the Berendsen barostat using a relaxation time of 2.0 ps.

To prepare the systems for productive MD simulations, the following steps were carried out: (1) 2000 steps of energy minimization, with all the protein atoms restrained; (2) 2000 steps of energy minimization, with all the protein Cα atoms restrained; (3) 2500 steps of energy minimization without any restraints; (4) 100 ps of constant volume simulation, with no restraints, and with the temperature increasing at constant rate of 3 K/ps from 0 to 300 K; (5) 500 ps of constant pressure simulation with the temperature kept at 300 K; (6) 400 ps of constant volume and temperature simulation. After these steps, 1.0 μs of MD simulation was carried out with constant temperature and volume. For each system, such procedure was repeated three times. PMEMD, from the Amber16 package^65^, was used to perform all the equilibrium MD simulations.

### Umbrella sampling

US simulations were employed to obtain the potential of mean force (PMF) associated to the open-close motions of GcoA:apo, GcoA:guaiacol and GcoA:catechol. The PMFs were computed along the reaction coordinate defined as ξ = RMSD(open)—RMSD(closed), where RMSD(open) is the RMSD measured from the most open structure obtained from the unbiased simulation of GcoA:apo, and RMSD(closed) is the RMSD measured from the crystal structure. The US simulations were performed using the colvar module of NAMD 2.12^66^ (with the same Amber force field employed for the unbiased simulations). In the RMSD calculations, only the Cα atoms of residues 1–31 and residues 150–206 were considered. These residues correspond to the region involved in the open-close motions of GcoA. We split the conformational space into 28 equally spaced windows, centered at ξ values between -4.5 Å and 3.6 Å, therefore with increments of δξ = 0.3 Å. Simulations within each window were restrained with a harmonic potential of the form (½)*k*(*ξ*-*ξ*0)2, with *k* = 10 kcal mol^−1^ Å^−2^. Simulation in an additional window centered at 2.2 Å and with *k* = 10 kcal mol^−1^ Å^−2^ was conducted to assure enough overlap between neighboring windows. Therefore, a total of 29 windows were used for the PMF calculation. Within each window, 260 ns of restrained MD simulation was carried out after a 10-ns equilibration (not considered in the PMF calculation), totalizing 270 ns/window. Initial configurations for the different windows of the GcoA:apo US simulations were taken from the unbiased simulation where we observed the closed-to-open transition. For the GcoA:guaiacol and GcoA:catechol systems, we started from the closed structure and followed a scheme where we used the equilibrated configuration of the previous window (window *i*) to start the simulation of the next window (window *i* + 1). The PMFs were obtained as the average of PMFs calculated for blocks of 10 ns. The Weighted Histogram Analysis Method^67^ was employed to reweight the biased histograms obtained with US MD.

### Density functional theory calculations

DFT calculations were performed using Gaussian 09^60^. A truncated model containing the porphyrin pyrrole core, Fe center and a methanethiol to mimic cysteine as Fe-axial ligand was used. Geometry optimizations and frequency calculations were performed using unrestricted B3LYP (UB3LYP)^68,69^ with the LANL2DZ basis set for iron and 6–31G(d) on all other atoms. Transition states had one negative force constant corresponding to the desired transformation. Enthalpies and entropies were calculated for 1 atm and 298.15 K. A correction to the harmonic oscillator approximation, as discussed by Truhlar and co-workers, was also applied to the entropy calculations by raising all frequencies below 100 cm^–1^–100 cm^–1^^70^. Single point energy calculations were performed using the dispersion-corrected functional (U)B3LYP-D3(BJ)^71,72^ with the LANL2DZ basis set on iron and 6-311+G(d,p) on all other atoms, within the CPCM polarizable conductor model (diethyl ether, *ε* = 4)^73,74^ to have an estimation of the dielectric permittivity in the enzyme active site. The use of a dielectric constant *ε* = 4 has been shown to be a good model to account for electronic polarization and small backbone fluctuations in enzyme active sites^75^. All stationary points were verified as minima or first-order saddle points by a vibrational frequency analysis. Computed structures are illustrated with CYLView.

### Data availability

Coordinates and associated structure factors have been deposited with the PDB (www.rcsb.org/) under accession codes 5NCB, 5OMR, 5OMS, 5OMU, 5OGX. Data supporting the findings of this study are available within the article (and its Supplementary Methods files) and from the corresponding authors upon reasonable request.

## Electronic supplementary material

Supplementary Information

Description of Additional Supplementary Files

Supplementary Data 1

Supplementary Movie 1

Supplementary Movie 2
