# Supplementary material for: A promiscuous cytochrome P450 aromatic O-demethylase for lignin bioconversion
Source: Nat Commun. 2018 Jun 27;9:2487. doi: 10.1038/s41467-018-04878-2 (PMC6021390; doi:10.1038/s41467-018-04878-2)
Supplement: Supplementary file 1 — Supplementary Information [file 41467_2018_4878_MOESM1_ESM.pdf]

Supplementary Information

A promiscuous cytochrome P450 aromatic *O*-demethylase for lignin bioconversion

Sam J. B. Mallinson *et al*.

Supplementary Methods

Structure solution and refinement of GcoA and GcoB
The data collected for GcoA with guaiacol were indexed, integrated, merged and scaled using Xia2[1-6] before solving and building using Crank2[7-14] and refinement with Refmac[15] and Coot.[16] Structures of GcoA with guaethol, syringol and vanillin were solved by molecular replacement using the original GcoA structure with guaiacol (PDB accession code 5NCB) with the heme and guaiacol ligands removed. The data were processed initially with Xia2 before using Phaser[17] from the Phenix suite[18] for molecular replacement and phenix.autobuild[19] for building. Iterative refinement was performed using phenix.refine[20] and Coot. The structure of the GcoB C-terminus was initially solved from a crystal grown in 0.2 M sodium phosphate monobasic monohydrate, 20% w/v polyethylene glycol 3,350 by molecular replacement using the C terminus of benzoate dioxygenase reductase (1KRH). This partial crystal structure was subsequently used for the structure solution of the full-length protein. Data were indexed, integrated, merged and scaled using Xia2[1-5, 21]. Molecular replacement was performed using Phaser[16] before building with Phenix.autobuild[19] and iterative refinement using Phenix.refine[20] and Coot[16]. Validation of these structures was performed using MolProbity[22] and the PDB validation server.

Supplementary Figures

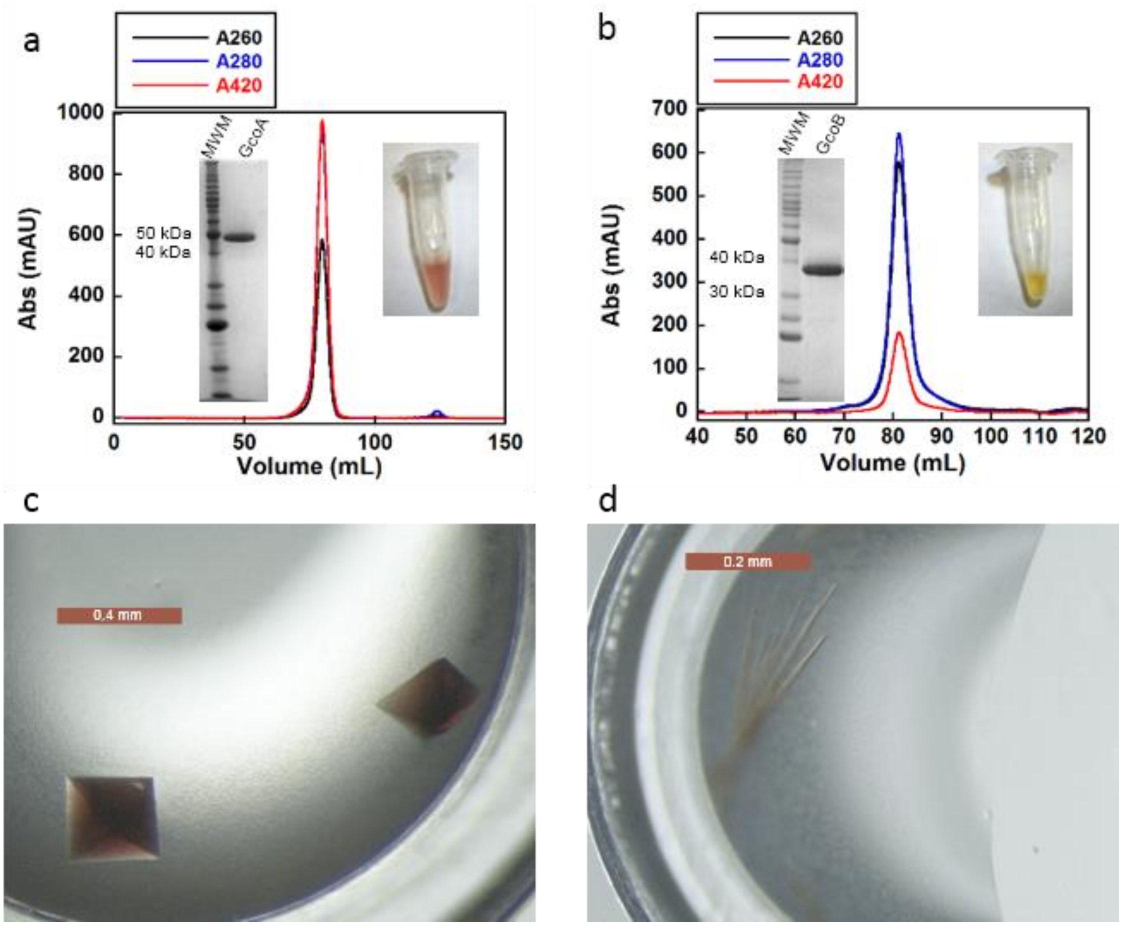

Supplementary Figure 1. Purification and crystallization of GcoA and GcoB. (**a**) A composite figure showing the final purification steps for GcoA. The size exclusion trace shows the protein elution from the column monitored at 3 wavelengths simultaneously. A single main peak was observed, with an equivalent absorbance at 280 nm (blue) and 420 nm (red), corresponding to a single band of 45 kDa by SDS-PAGE (inset). The purified and concentrated protein was seen to have a red-brown color due to the incorporation of heme (inset is a photograph of a protein solution of GcoA in an 1.5 ml Eppendorf tube). (**b**) The equivalent final purification step for GcoB is shown. A single peak is observed corresponding to a single band of 36 kDa by SDS-PAGE. The incorporation of FAD resulted in a bright yellow color (inset is a photograph of protein a solution of GcoB in a 1.5 ml Eppendorf tube). (**c**) Optimized pyramidal crystals of GcoA grown by vapor diffusion display a deep red-brown color and could be consistently grown to a maximum dimension of 400 µm. (**d**) Crystals of GcoB we colored yellow and generally appeared in a needle conformation with maximum dimensions up to 500 µm. Single crystals were obtained for diffraction experiments by careful manipulation of the needle clusters shown here.

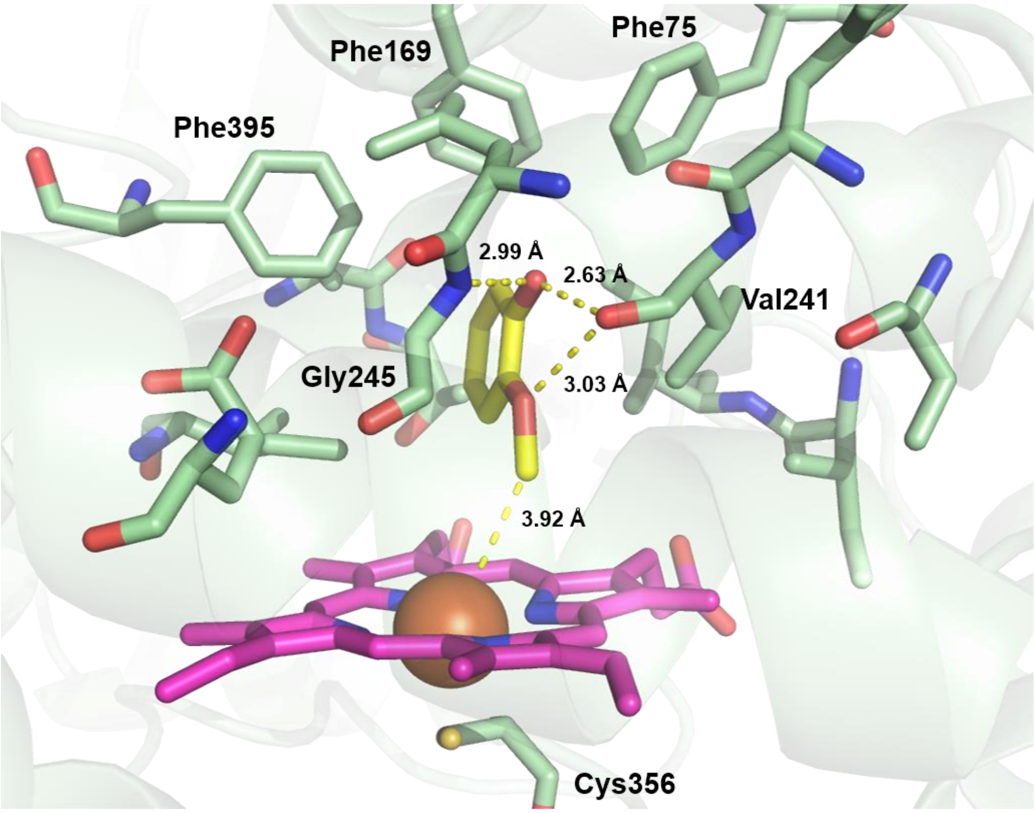

Supplementary Figure 2. Coordination of guaiacol in the active site of GcoA. A cartoon representation of the active site of GcoA is shown, with key residues rendered as sticks (green) and the heme (pink) shown with the central iron (orange sphere**).** Guaiacol (yellow) is coordinated by a combination of hydrophobic and hydrophilic residues. The aromatic ring is primarily coordinated by three hydrophobic residues, Phe75, Phe169, and Phe395. The oxygen atoms comprising the hydroxyl and methoxy groups of guaiacol are coordinated by the backbone hydroxyl and peptide groups from Val241 and Gly245, respectively. The distances of these interactions are indicated with yellow dashed lines. The distance from the methoxy carbon to the center of the heme iron is 3.92 Å. The position of Cys356 is shown relative to the heme.

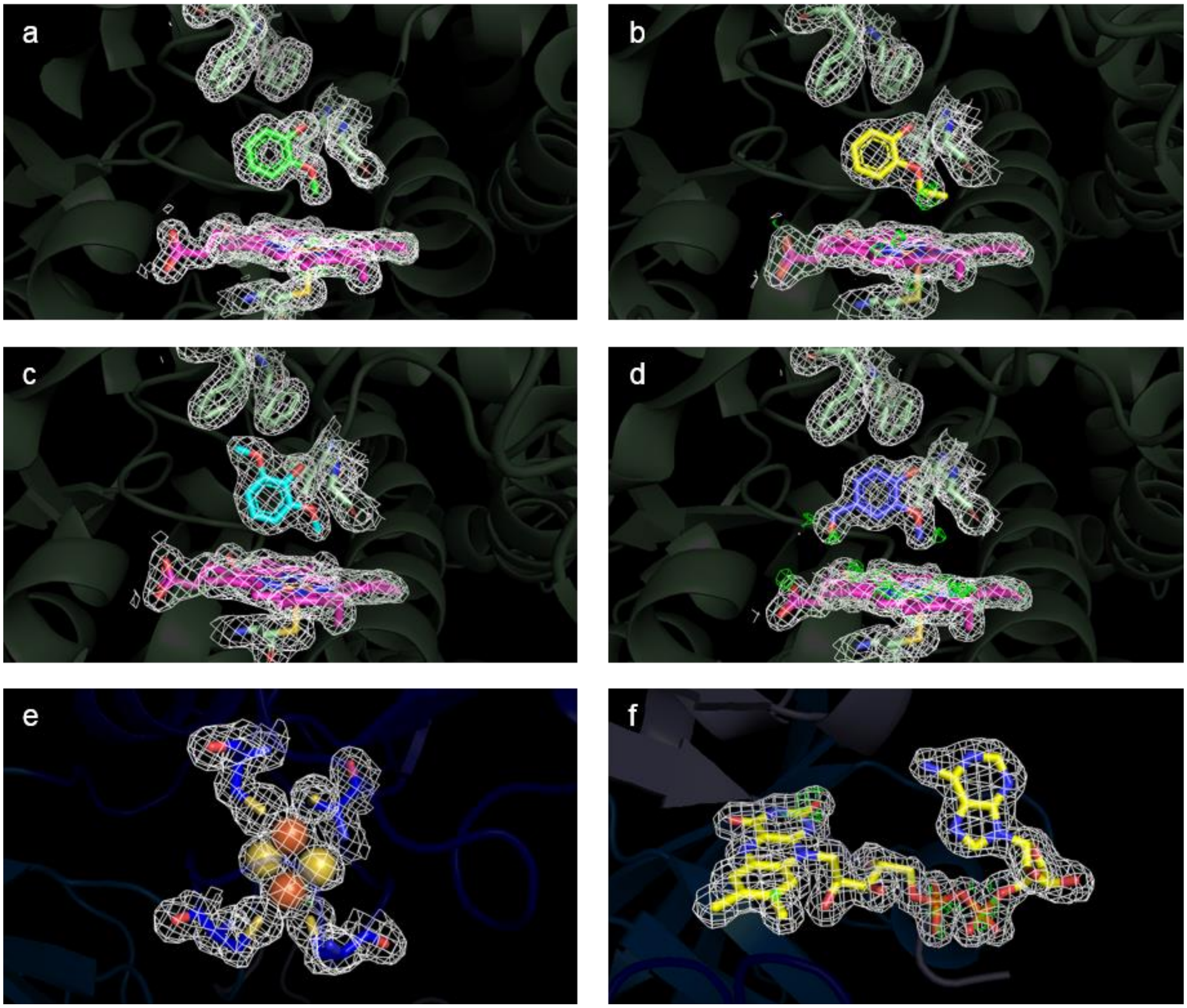

Supplementary Figure 3. Electron density of GcoA and GcoB structures. Representative electron density maps were rendered as white mesh for $2F_o$-F_c maps contoured at 1σ, and green (positive) and red (negative) mesh for F_o-F_c maps contoured at 3σ. (**a**) Electron density is shown for GcoA-bound guaiacol (5NCB), (**b**) guaethol (5OMS), (**c**) syringol (5OMU) and, (**d**) vanillin (5OMR). In each case, electron density was rendered around the substrate and the heme group along with the main coordinating residues, Phe75, Phe169, Val241, Gly245 and Cys356. (**e**) Electron density for GcoB highlighting the FeS cluster with coordinating residues Cys37, Cys42, Cys45 and Cys77 and (**f**) highlighting the bound FAD cofactor of GcoB (5OGX).

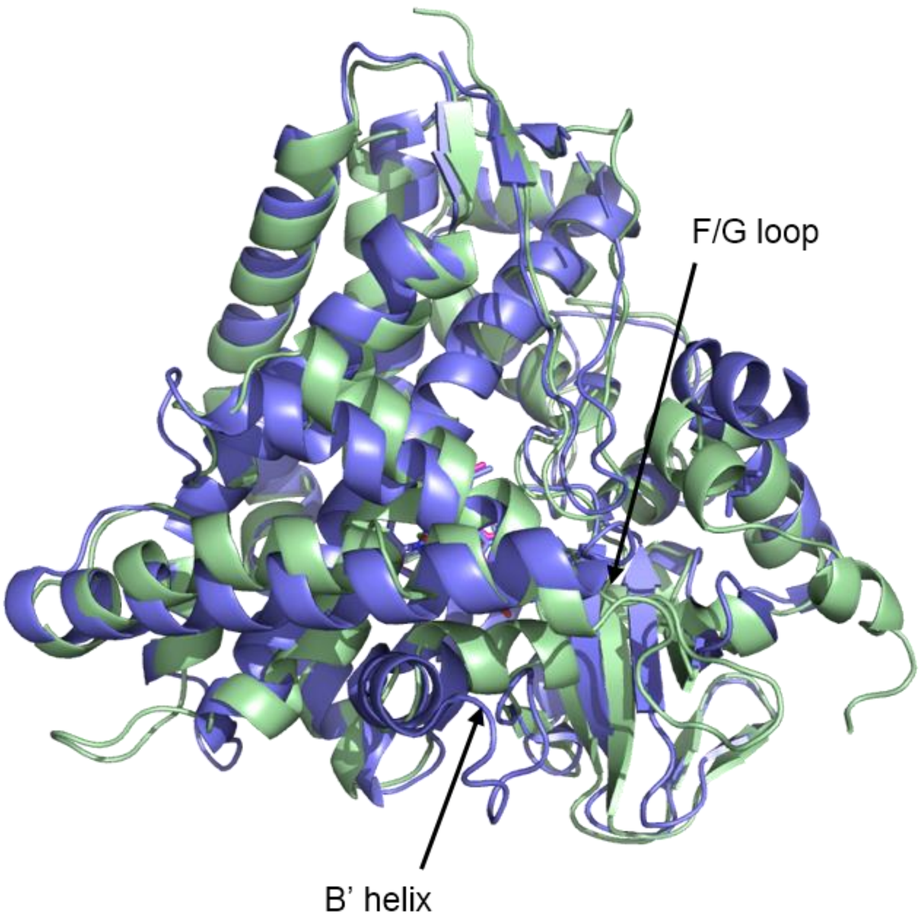

Supplementary Figure 4. Structural alignment of GcoA with *B. subtilis* P450BioI. P450BioI (blue, PDB accession code 3EJB) is the highest identity homolog of GcoA (green) in the PDB, with 26% identity and an RMSD of 1.87 Å. The similarities can be seen by the close overlap of many of the α-helices and β-sheets throughout the structures. Key differences in the two proteins can be found around the substrate access channel: The F/G loop is a feature of P450s that undergoes a breathing motion to allow substrate access/product release. In P450BioI this is significantly shorter than that of GcoA and the B' helix is positioned further from the substrate access channel with a relative rotation of about 90°. This may reflect the difference in the substrates of the two proteins since GcoA catalyzes the demethylation of small aromatic molecules, compared to P450BioI which catalyzes the oxidative cleavage of long chain fatty acids.[23]

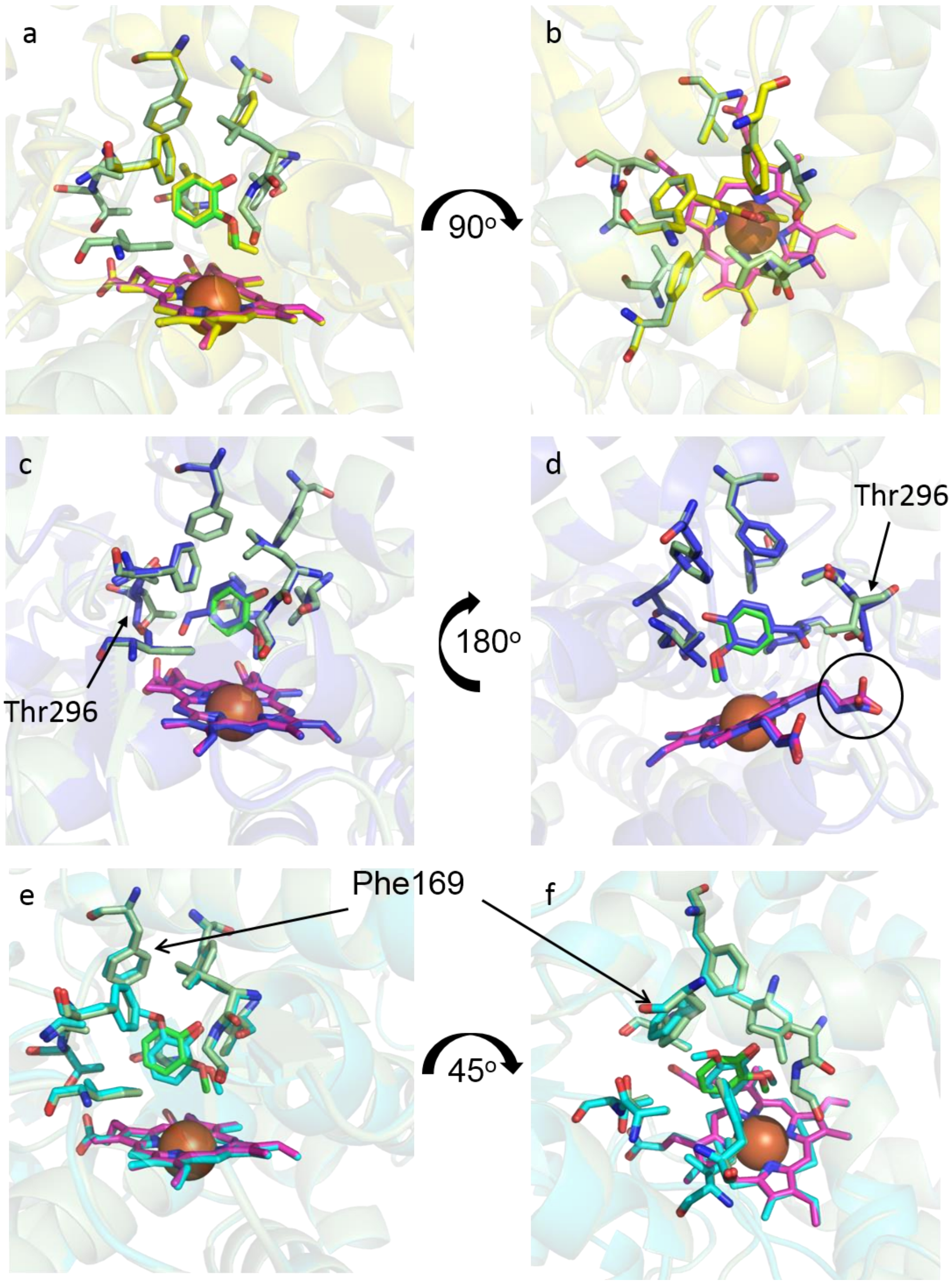

Supplementary Figure 5. Comparison of alternative ligand coordination in GcoA. Each panel shows the active site of the guaiacol bound structure aligned with the guaethol, vanillin or syringol bound structure. The protein chain of the guaiacol bound structure is shown in light green with the guaiacol molecule in green and the heme group in magenta. (**a**) The guaethol bound structure of GcoA is shown in yellow; there is a slight rotation of the aromatic ring to accommodate guaethol compared to guaiacol, but no discernible rearrangement of the active site. (**b**) Shows the active site alignment from **(a)** rotated through 90° to show the plane of the ligand aromatic ring. (**c**) The vanillin bound structure is shown in blue; the aromatic ring of vanillin is productively accommodated into the hydrophobic active site cavity, however, the extra aldehyde group at the 4 position requires a significant rearrangement. Shown here is the shift in the sidechain of Thr296 relative to its position in the guaiacol structure caused by the aldehyde group of vanillin. The positioning of Thr296 is the same for the guaiacol, guaethol and syringol structures. This sidechain movement causes the shift of the heme propionate group. In addition, the backbone of the protein is displaced by about 1Å over quite a large surrounding area. (**d**) shows the active site alignment from **(c)** rotated through 180° to show the movement of the Thr296 residue and the heme propionate group (circled). (**e**) The syringol bound structure is shown in cyan; the hydroxyl group of syringol and guaiacol are held in roughly the same position, though a rotation about the hydroxyl oxygen of syringol compared to guaiacol shifts the aromatic ring closer to the heme moiety. Despite this, the active site still exhibits an expansion to accommodate the extra methoxy group of this substrate. The three residues Phe75, Phe169 and Phe395 exhibit varying degrees of translation (Phe169 being the greatest) with subsequent shifting of the protein backbone. (**f**) shows the active site alignment from (**e**) rotated through 45°.

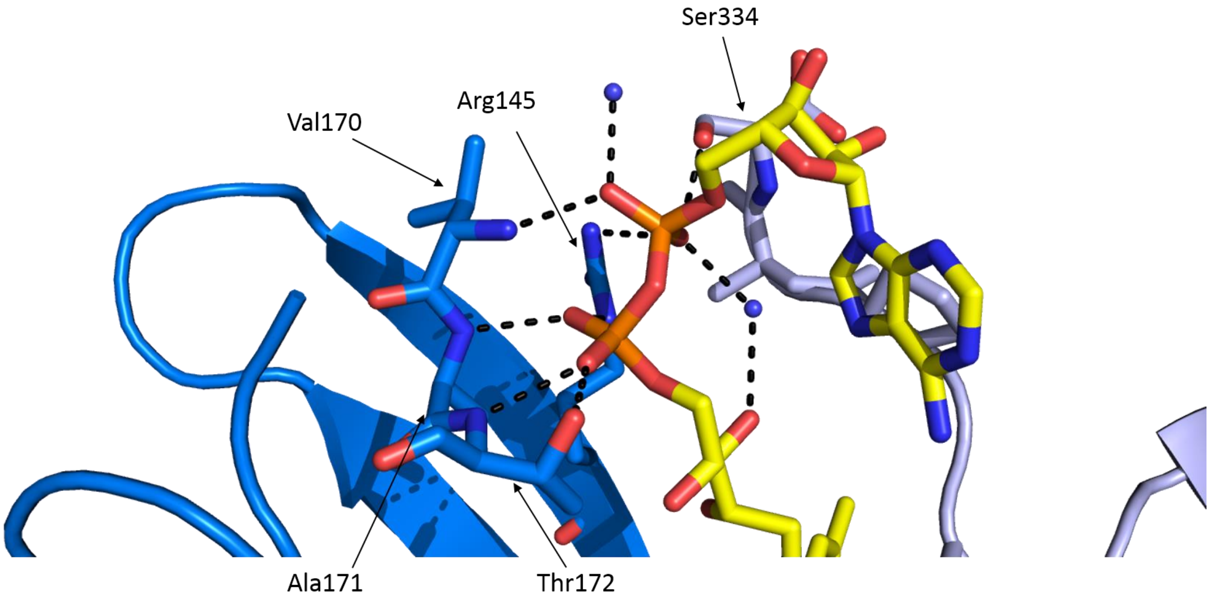

Supplementary Figure 6. Coordination of FAD in GcoB. FAD is shown in yellow with the FAD binding domain of GcoB in light blue and the C-terminal NADH binding domain of GcoB in grey. Protein and FAD nitrogen, oxygen and phosphorus atoms are coloured blue, red and orange respectively. Blue spheres detail the position of ordered water molecules visible in the structure and black dashed lines represent hydrogen bonds. Three consecutive residues, Val170, Ala171 and Thr172 all contribute backbone hydroxyls or peptide nitrogens to coordinate the diphosphate. In addition, Thr172, Arg145 and Ser334 (the last residue in the protein), all contribute interactions through their side chains.

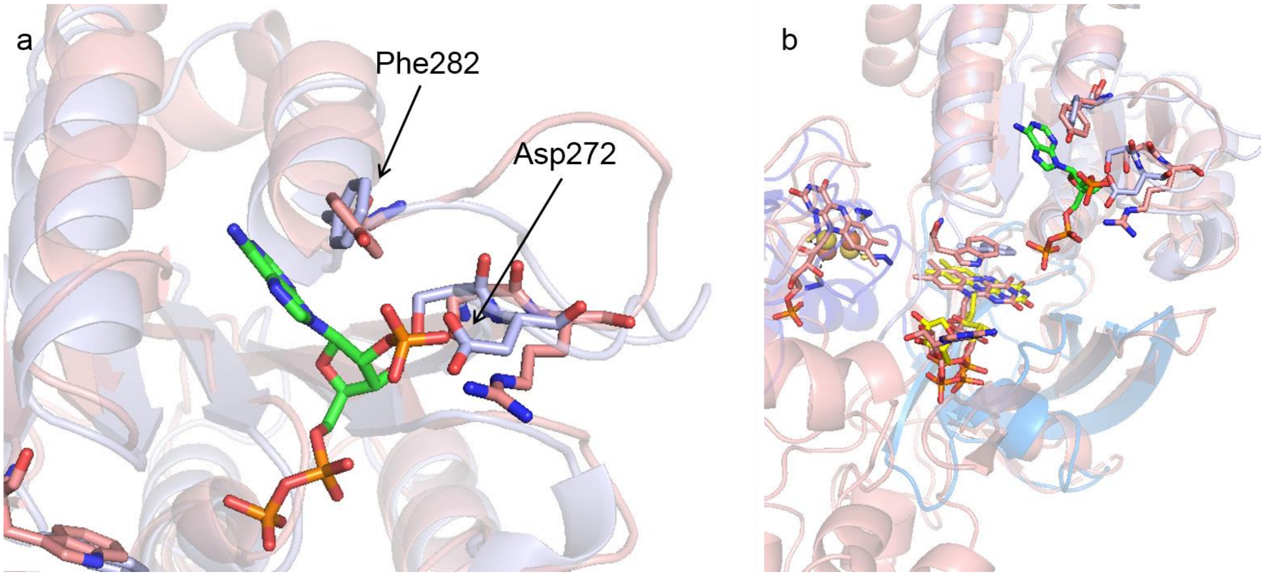

Supplementary Figure 7. Structural basis for NAD vs NADP discrimination in GcoB. Here we compared the NAD binding pocket of GcoB with that of the fully occupied NADPH binding pocket in an X-ray structure of the CPR from *Rattus norvegicus* (1AMO). (**a**) In the CPR structure from *R. norvegicus* (bronze), the adenine group of NADPH (green) is coordinated by the aromatic ring of a tyrosine side chain. A phenylalanine at an equivalent position in GcoB (light blue) may perform a similar stabilizing function with NADH. The 2' phosphate on the ribose ring is in close proximity to an arginine in CPR which is likely responsible for stabilizing the interaction of CPR and NADPH. In GcoB, this arginine is replaced by Asp272, which would appear to sterically and/or electrostatically preclude the phosphate group of NADPH thus resulting in the preferential binding and turnover of NADH compared to NADPH. This provides insight into the discrimination between these two ligands observed in the comparative activity measurements (**Supplementary Fig. S16**.) (**b**) An overview of the same superposition shows the proximity of the putative NADH binding site in GcoB to the FAD binding site.

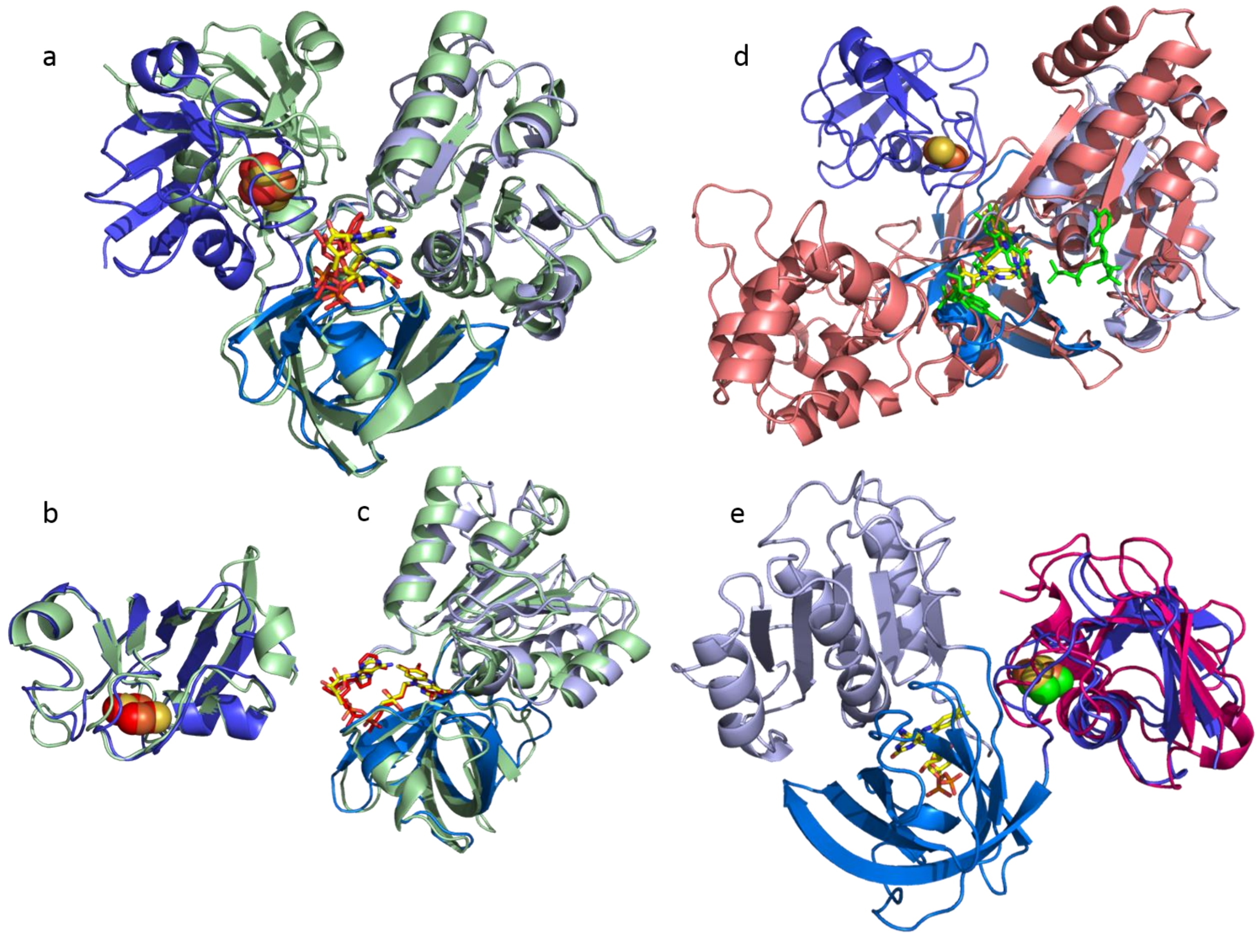

Supplementary Figure 8. GcoB structural alignments. (**a**) GcoB (dark blue, blue and light blue) aligned with BenC (1KRH[24], dark green). (**b**) 2Fe2S domains of GcoB (dark blue) and BenC (green) and (**c**) FAD/NADH domains of GcoB (blue and light blue) and BenC (green). (**d**) Alignment of the FAD/NADH domain of GcoB with a canonical FAD/NAD(P)H domain (1AMO, FAD/FMN type P450 reductase from *R. norvegicus*[25], light red). (**e**) Alignment of putidaredoxin (1XLP from *P. putida*, pink) with the 2Fe2S domain of GcoB[26]. GcoB bears structural homology to different classes of oxidoreductase protein. The closest structural homolog, BenC from *Acinetobacter baylyi* ADP1, was used as a productive search model in molecular replacement for GcoB. BenC is a reductase for benzoate 1,2-dioxygenase, a class 1B Rieske dioxygenase[27]. As seen in (**a**), the 2Fe2S domains and FAD/NADH domains of GcoB and BenC are rotated differently relative to each other. When taken separately the two domains align very closely (**b, c**). While the terminal protein of the electron transport chains for Benzoate dioxygenase and GcoAB are altogether different classes of proteins, they share a common function in metabolizing small aromatic molecules, a potential explanation for the evolutionary origin of GcoB as a P450 reductase. While the overall fold of GcoB is quite different to any cytochrome P450 reductase found in the PDB, when divided into its modular domains, the structural similarities with its functional homologues can be seen. (**d**) shows GcoB aligned with the FAD and NAD(P)H binding domains of a P450 reductase from a 2-component type system, containing an FAD-FMN type reductase. The FMN domain has been removed here. The FAD and NADH domains of GcoB show strong homology with corresponding sections of 1AMO[28], in particular in the coordination of the cofactors, though 1AMO contains an extra chain of amino acids in the FAD binding domain not found in GcoB. The ferredoxin domain of GcoB is a homolog of ferredoxin domains from three component systems (**e**). These alignments suggest that GcoAB represents a new structurally uncharacterized cytochrome P450 system, a 2-component system utilizing an FAD-ferredoxin type reductase rather than an FAD-FMN type reductase. While the structure of GcoB is highly conserved with Rieske-type reductase proteins, this protein is functionally homologous to a reductase from a 2-component cytochrome P450 system. In addition, GcoB maintains some structural similarity with the P450 reductase proteins with which it shares a function. This protein can be seen as a new 2-component system reductase in which the FMN-domain of an FAD-FMN type reductase has been replaced by the FeS domain of a 3-componenet P450 system.

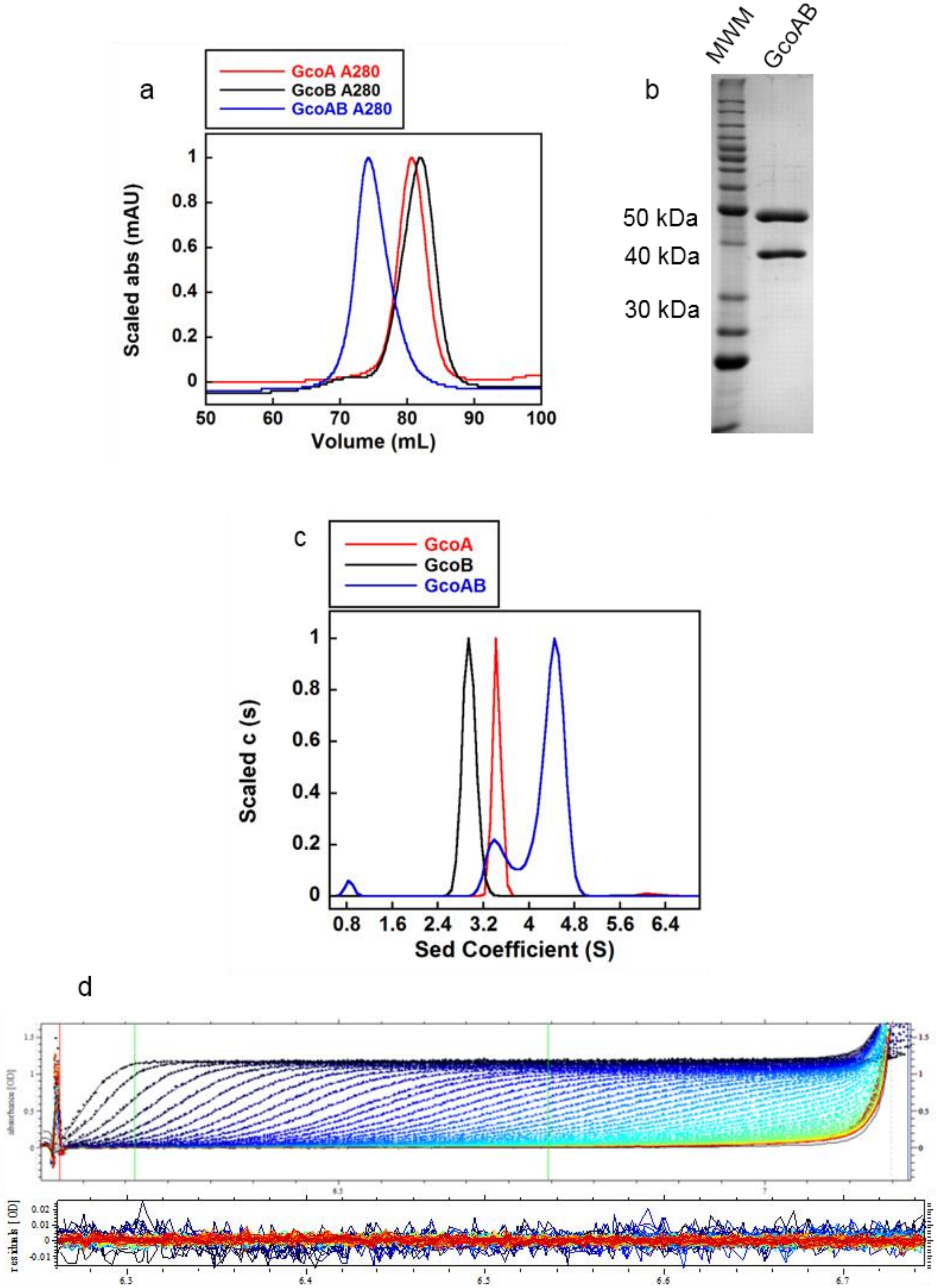

Supplementary Figure 9. Hydrodynamic analysis of GcoA, GcoB, and GcoAB. (**a**) An overlay of 3 separate size exclusion chromatography experiments with GcoA (red), GcoB (black), and GcoAB (blue). The single species detected in the GcoAB trace demonstrates that the two proteins remain as a dimer in solution. (**b**) SDS-PAGE of the sample that eluted from GcoAB size exclusion chromatography. Lane 1 (MWM) is Benchmark molecular weight marker and lane 2 is GcoAB showing the two species at molecular weights of 45 and 35 kDa, corresponding to GcoA and GcoB, respectively. (**c**) Sedimentation velocity AUC values of 3.53S, 2.92S and 4.37S for GcoA, GcoB and, GcoAB, respectively. In the GcoAB run, an excess of GcoA was loaded and this is seen as a separate species at the corresponding value of 3.5S. (**d**) Data plot of AUC velocity experiment of GcoAB. Raw data points and fitted curves are plotted ranging in color from dark blue at the beginning of the run, through to green and red at the end of the run. A corresponding residuals plot is provided below the main trace and demonstrates evenly distributed values.

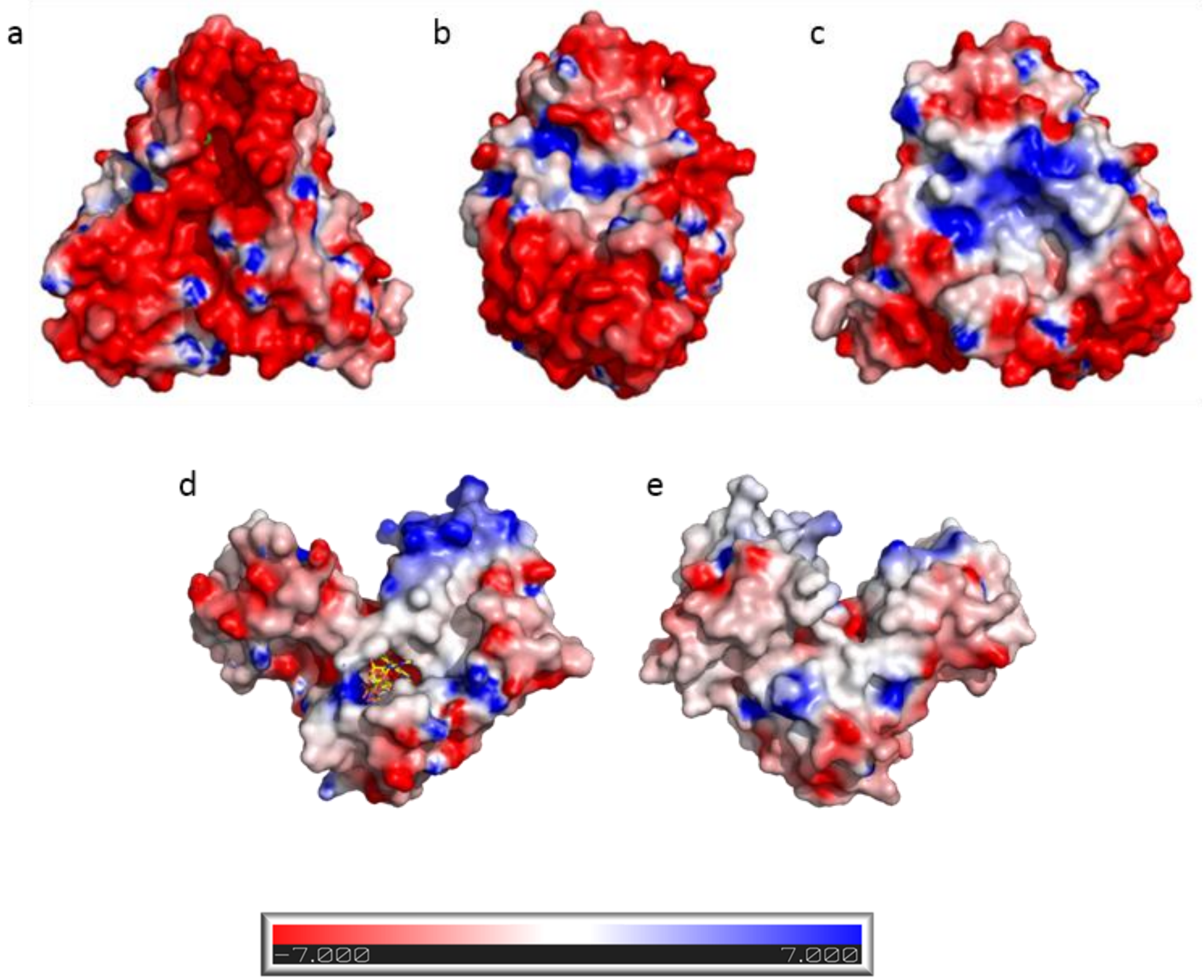

Supplementary Figure 10. Electrostatic potential of GcoA and GcoB. (**a-c**) Show three different orientations of GcoA rendered as surface models and colored according to electrostatic potential between -7 *kT/e* and +7 *kT/e*. The surface of GcoA is highly charged and mainly acidic with the exception of a basic patch on the proximal face (**c**). The dipole created by this difference in charge across the protein is thought to aid in electron transfer.[24] (**d, e**) Two orientations of a correspondingly colored GcoB model show that it is generally less charged than GcoA. P450 reductase proteins typically have an acidic patch that forms the interface with the basic patch of the P450 reductase partner. A candidate for this acidic binding patch, which is often found close to the Iron-Sulfur cluster in other systems, is not obvious in GcoB.

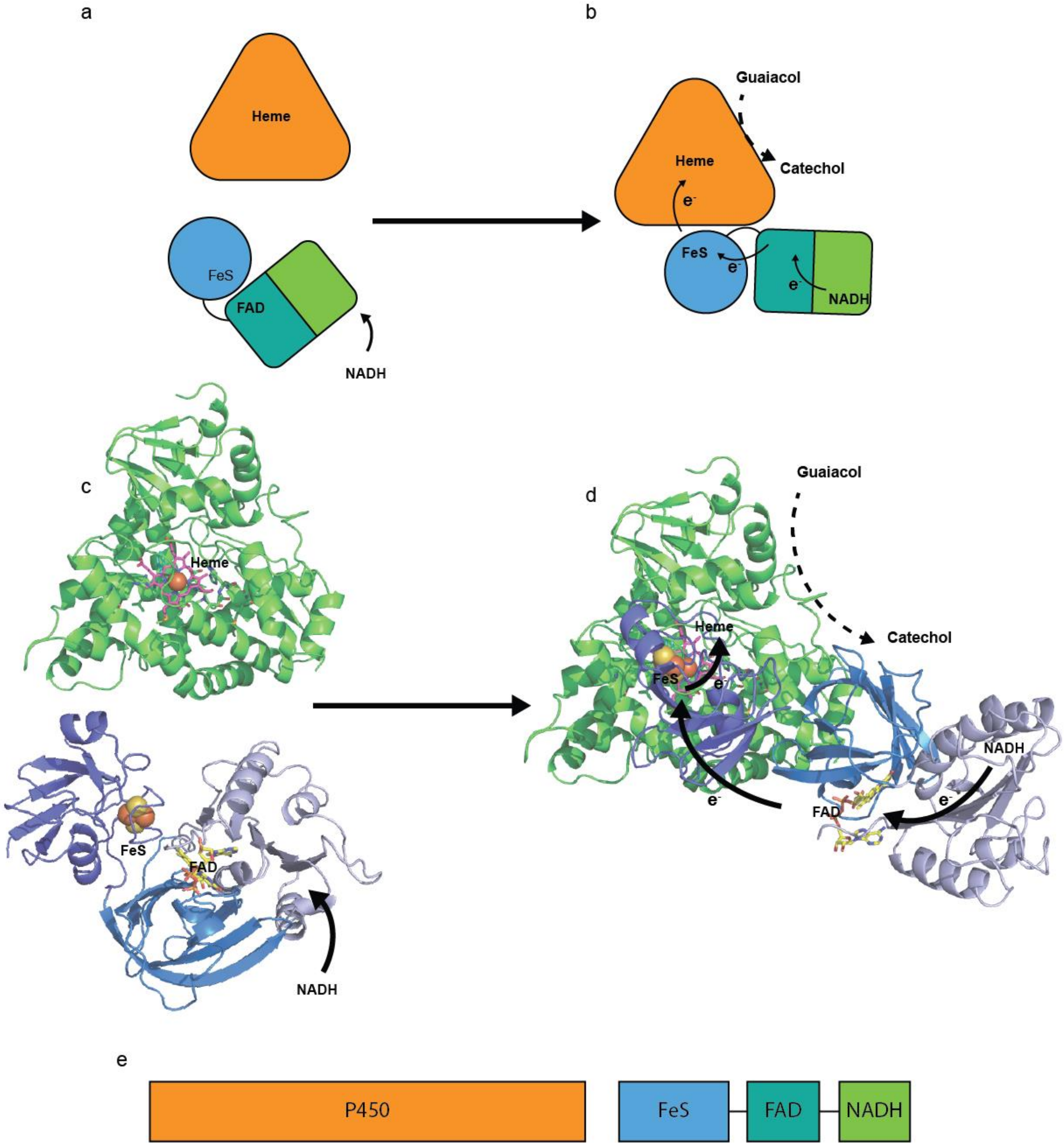

Supplementary Figure 11. Predicted GcoAB domain arrangement and potential electron flow. This schematic was based on information from SAXS studies by Huang et al.[29], which demonstrated the flexible motion of FMN-FAD type cytochrome P450 reductase proteins required for interaction with their partner P450; and Tripathi et al.[26], where the structure of cytochrome P450cam cross-linked to Pdx revealed that without a domain movement of some kind, the ferredoxin domain of GcoB would be occluded from interacting with the proximal face of GcoA. This concurs with the structural alignments of GcoA and GcoB with P450cam:Pdx (4XJ1[30]). (**a**) and (**b**) show a schematic of the proposed interaction. Orange represents the P450 domain, GcoA, and blue, dark green and green represent the FeS, FAD and NADH binding domains of GcoB, respectively. (**c**) and (**d**) show a structure based representation of the proposed interaction. This was generated in PyMol by manipulating the individual domains of GcoB manually after aligning GcoA and the ferredoxin domain of GcoB with the cross-linked crystal structure of P450cam and Pdx. The green structure is GcoA while the dark blue, blue and light blue structures are the FeS, FAD and NADH domains of GcoB, respectively. The black solid lines represent the movement of electrons through the system and the black dashed line represents the demethylation reaction converting guaiacol to catechol. (**e**) Linear diagram of the domain arrangement in this system.

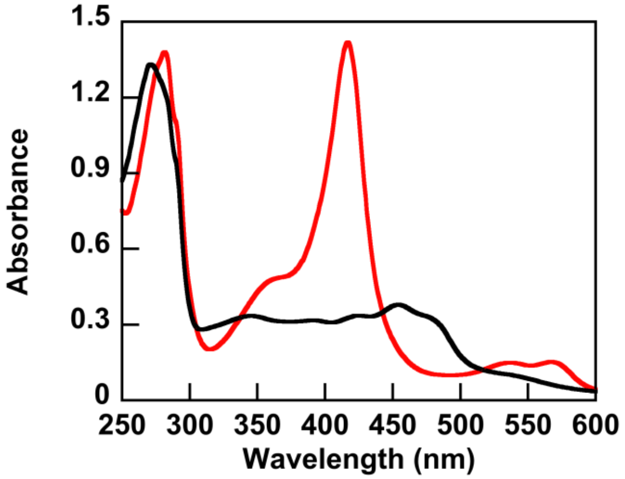

Supplementary Figure 12. UV/vis spectra of GcoA and GcoB in their oxidized forms. Spectra for GcoA (12 µM, red trace) and GcoB (17 µM, black trace) were measured in 25 mM HEPES, 50 mM NaCl, pH 7.5. In addition to the protein-associated absorbance centered at 280 nm, GcoA exhibits a sharp Soret peak at 420 nm and heme α- and β-bands (Q-bands) at 537 and 567 nm. GcoB has an absorbance maximum at 454 nm, a region typically associated with oxidized FAD. The small peak at 423 nm and the shoulder at 480 nm are most likely due to the presence of the 2Fe-2S cluster.[31] Heme, FAD, and 2Fe-2S occupancies for these proteins were measured at 0.9, 0.6 and 0.8 equivalents per protein, respectively, via methods described below and in the text.

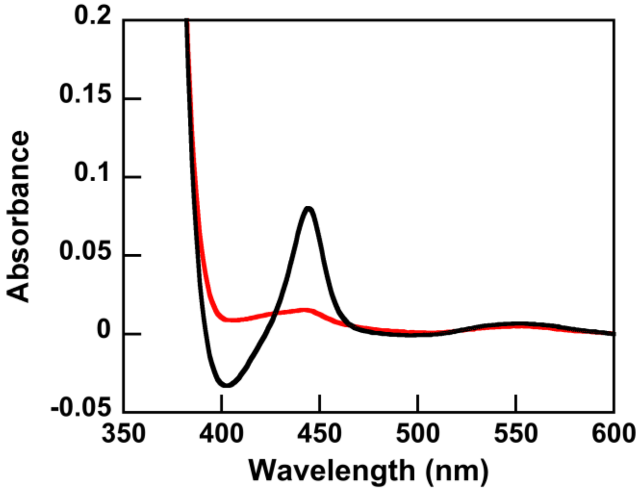

Supplementary Figure 13. CO binds to ferrous GcoA, indicating the presence of catalytically active heme.[32] A solution containing 0.94 µM GcoA in 25 mM HEPES, 50 mM NaCl, 1.0 mM EDTA, 20% glycerol, 0.5% sodium cholate, 0.4% non-ionic detergent, pH 7.5, 25 ºC was put into a cuvette and the baseline taken. CO gas was bubbled into one of the cuvettes (sample); the second cuvette was the reference sample. A spectrum was measured following addition of excess dithionite to both the sample and reference cuvettes. The reduced heme bound to CO, leading to the spectral feature at 447 nm (black trace), characteristic of cytochrome P450s. This spectrum was compared to the reference sample (no CO), shown in the red trace. The amount of active heme, represented by the absorbance at 447 nm, is 0.72 µM (0.78 eq/GcoA monomer). The inactive heme, represented by the trough at 420 nm, is 0.07 µM (see Experimental section). Amounts of active and inactive heme were found using the following relationships between the absorbances at 420, 450, and 490 nm[32]:

$(\Delta A450 - \Delta A490)/0.091$ = nmol of P450 per mL (1)

$[(\Delta A420 - A490)_{observed} - (A450 - A490)_{theoretical}]/0.110$ = nmol of cytochrome P420 per mL (2)

nmol of P450 per mL x $(-0.041) = (\Delta A420 - A490)_{theoretical}$ (3)

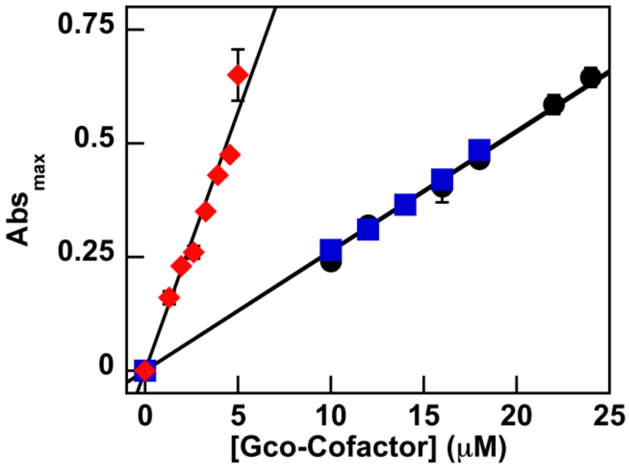

Supplementary Figure 14. Extinction coefficients for GcoA/B bound cofactors. a. Absorbance spectra were measured for both GcoA and GcoB and the change in absorbance at 417 nm (the heme Soret maximum), 423 nm (FeS cluster absorbance maximum), and 454 nm (FAD absorbance maxium) were noted. GcoA-bound heme was quantified using the CO-binding assay (see text and Figures S1-S2). $Abs_{417\ nm}$ was plotted against [GcoA-heme] (red diamonds) and the slope of the fitted line (Kaleidagraph) was used to determine an extinction coefficient of 114 ± 4 mM^{-1} cm^{-1} for GcoA-heme (ϵ = concentration x pathlength x $\Delta absorbance^{-1}$). FAD was extracted using saturated ammonium sulfate, 7% v/v 96% H_2SO_4 (final pH = 2.0) from a series of concentrations of GcoB and quantified using the extinction coefficient for FAD (11.3 mM^{-1} cm^{-1}). The resulting linear graph (blue squares) yields a slope corresponding to an extinction coefficient of 26.6 ± 0.2 mM^{-1} cm^{-1} for GcoB-FAD. The 2Fe-2S cluster content in GcoB was quantified via the bathophenoanthraline disulfonate colorimetric assay (see text). The resulting linear graph (black circles) yields a slope corresponding to an extinction coefficient of 25.2 ± 0.1 mM^{-1} cm^{-1} for GcoB-2Fe-2S. Error bars represent ± 1 standard deviation of triplicate runs.

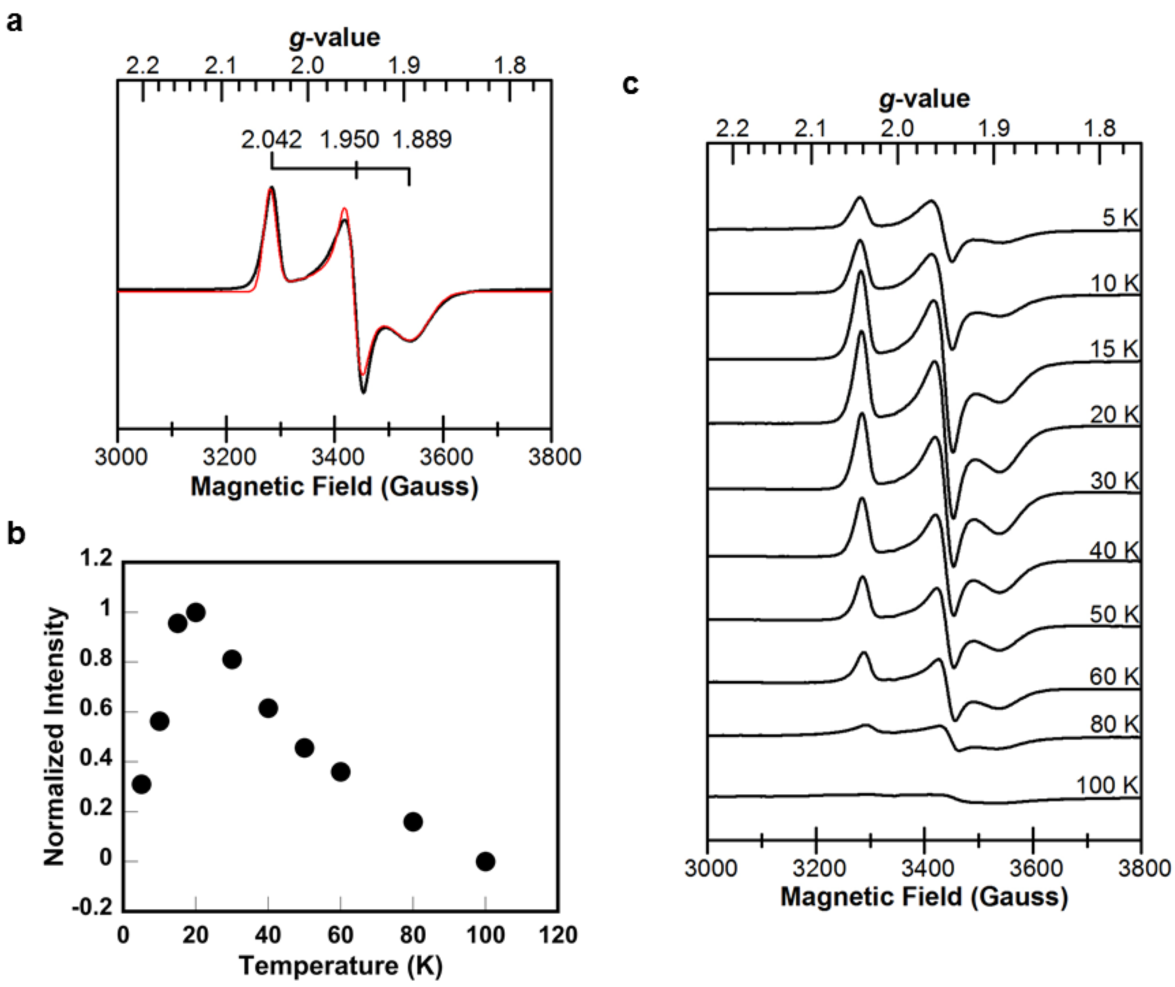

Supplementary Figure 15. CW X-band EPR spectrum of GcoB indicates presence of 2Fe-2S cluster. (**a**) 150 µM GcoB (50 mM Tris, 200 mM NaCl, 5% glycerol, pH 8.0) was reduced with 10 mM sodium dithionite and its EPR spectrum measured (EPR parameters: microwave frequency, 9.380 GHz; microwave power, 1.0 mW; modulation frequency, 100 kHz; modulation amplitude, 10.0 G; sample temperature, 20 K). The enzyme displays a rhombic signal indicative of a [2Fe-2S] cluster. The experimental spectrum is shown as a red trace, and the simulated spectrum (EasySpin software package) in black, yielding g-values of 2.041, 1.950, and 1.889. (**b, c**) The temperature dependence of the rhombic EPR signal reflected typical relaxation properties for [2Fe-2S] clusters.[30] The signal maximized at 20 K, becoming broadened beyond detection at 100 K.

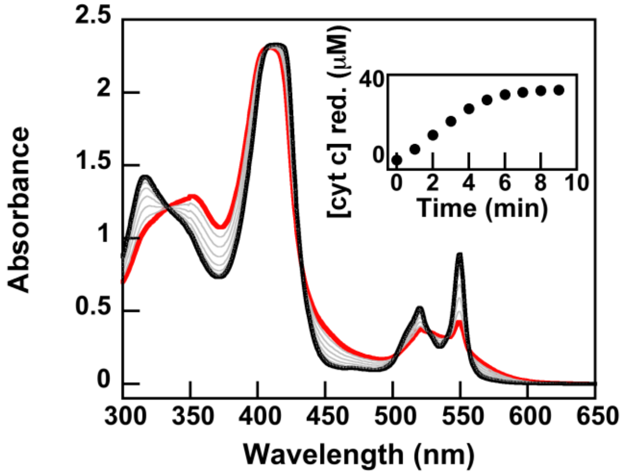

Supplementary Figure 16. GcoB catalyzes NADH-mediated reduction of cytochrome c (cyt c). GcoB and (4 nM) and cyt c (42 μM) were combined (in 25 mM HEPES, 50 mM NaCl, pH 7.5, 25 °C) and the spectrum recorded (red trace). Following addition of an approximate 2-fold molar excess of NADH relative to cyt c (100 μM), cyt c was reduced over several minutes (gray spectra measured 1 per min), as evidenced by the increase in the absorbance at 550 nm (final spectrum: black trace). The concentration of reduced cytochrome c was determined via its extinction coefficient at 550 nm (ε_{550nm} = 21,000 M^{-1} cm^{-1})[32], with the total amount of cytochrome c reduced being the difference between the black and red trace. Inset: Change of [cytochrome c] reduced over time yielded a progress of reaction curve, where the specific activity was calculated from the initial, linear portion. The specific activity, referenced to [GcoB] is 1700 ± 200 nmol cyt. c min^{-1} nmol $GcoB^{-1}$ (average of 3 measurements).

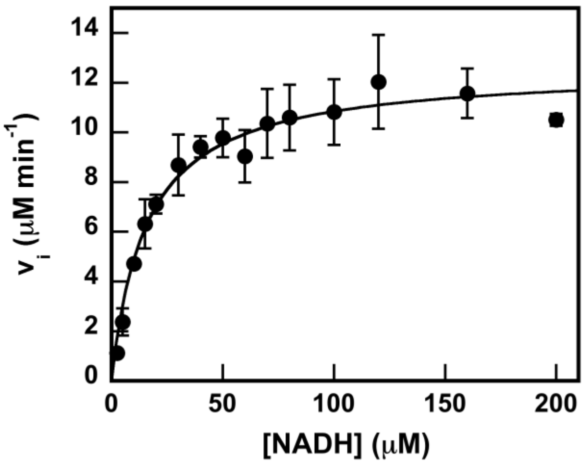

Supplementary Figure 17. Steady state kinetics of the reductase reaction catalyzed by GcoB. The initial velocity of NADH-mediated cyt c reduction was monitored at 550 nm as shown in Figure S5 via the initial linear portion of the progress of reaction curve. Initial rates were plotted as a function of [NADH] and the following kinetic parameters determined by fitting the data to the Michaelis Menton equation: k_{cat} = 44 ± 1 sec^{-1}, K_M = 0.016 ± 0.002 mM, k_{cat}/K_M = 2750 ± 300 mM^{-1} sec^{-1} (in 25 mM HEPES, 50 mM NaCl, pH 7.5, 25°C) The k_{cat} and k_{cat}/K_M(NADH) for GcoB are ~6.5 and ~25-fold greater than the overall demethylase reaction with both GcoA/B and NADH, indicating that the reduction of GcoA is not rate limiting. Error bars are given as ± 1 standard deviation of three or more measurements.

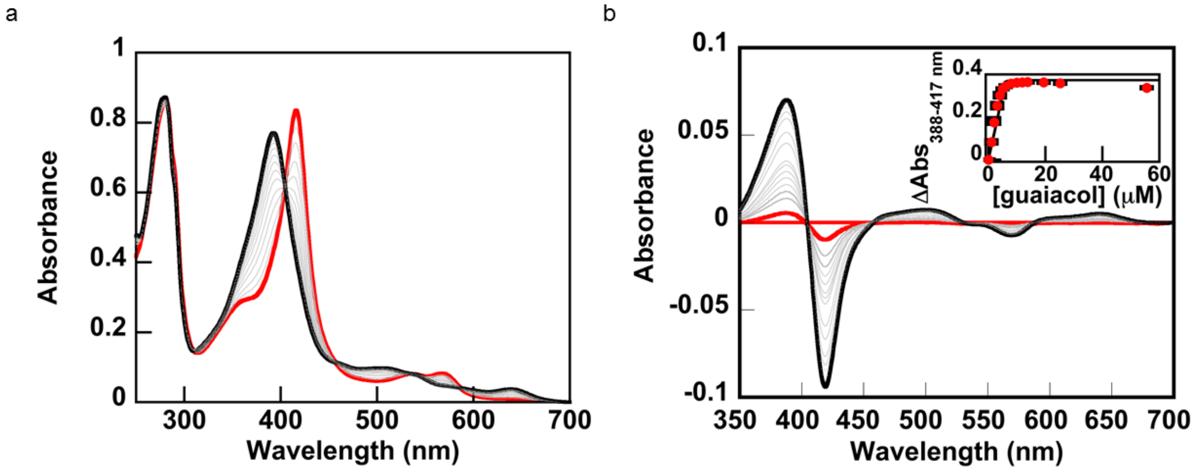

Supplementary Figure 18. Spectral shift upon addition of guaiacol to GcoA illustrates that the substrate binds in the active site. The spectrum of ferric 6 μM GcoA (red line; 25 mM HEPES, 50 mM NaCl, pH 7.5) was measured. (Left) Addition of 0-60 μM guaiacol produced a spectral shift (black trace) from 420 nm to 388 nm, indicating the binding of guaiacol in the distal pocket above the heme and the conversion of the six-coordinate, low-spin aquo complex to the five-coordinate, high-spin ferric heme. (Right) The spectrum of GcoA without guaiacol (red trace) was subtracted from the substrate-bound spectra (black trace) and plotted to generate a difference spectrum. Inset: Difference spectra were generated as shown in the left-hand panel for guaiacol concentrations at 1 μM increments from 0 mM (red trace) to 30 μM (gray traces). A final measurement was made at 60 μM guaiacol (black trace). The absorbance intensity at 388 nm was plotted as a function of [guaiacol] and fit to a quadratic equation (Experimental methods, eqn 6) to determine a K_D of 0.006 ± 0.002.

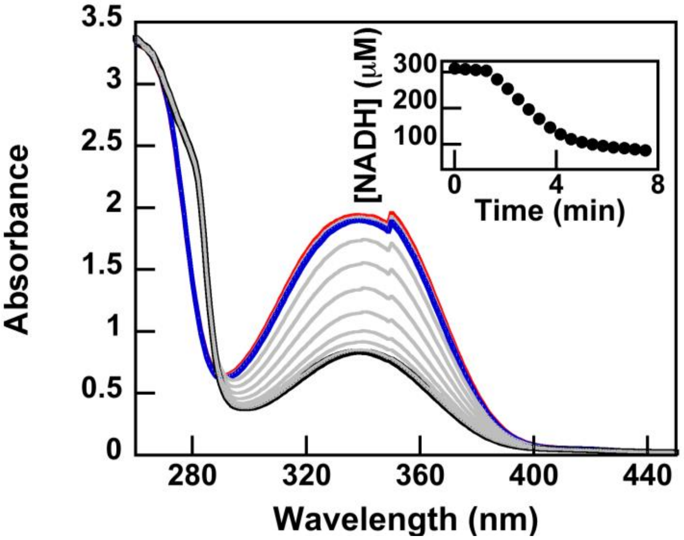

Supplementary Figure 19. NADH disappearance in the GcoA/B-catalyzed reaction was monitored via UV/vis. NADH oxidation by GcoB is coupled with the O_2-dependent oxidative demethylation of guaiacol by GcoA, resulting in to the production of formaldehyde and catechol. The overall reaction using a large stoichiometric excess of guaiacol relative to the enzymes (300 μM), GcoA/B (0.2 μM each), and saturating NADH (300 μM) in air was monitored by the change in absorbance due to NADH oxidation (NADH λ = 340 nm, ε_{340nm} = 6.22 mM^{-1} cm^{-1}) in 25 mM HEPES, 50 mM NaCl, pH 7.5, 25 °C. NADH and GcoA/B (red trace) did not react until after the addition of guaiacol (blue trace, t = 0 min). The reaction was monitored over time (gray spectra measured each minute) until the spectra stopped changing (black trace). Inset: Progress of reaction curve for NADH oxidation over time. The data approach 0 μM NADH within about 8 min (1.25 min background oxidation, 6.75 min substrate-initiated reaction). Fitting the initial linear portion of the curve to a line gives a specific activity of 4.6 mM NADH sec^{-1} [mM $GcoA]^{-1}$ discernable from the slope.

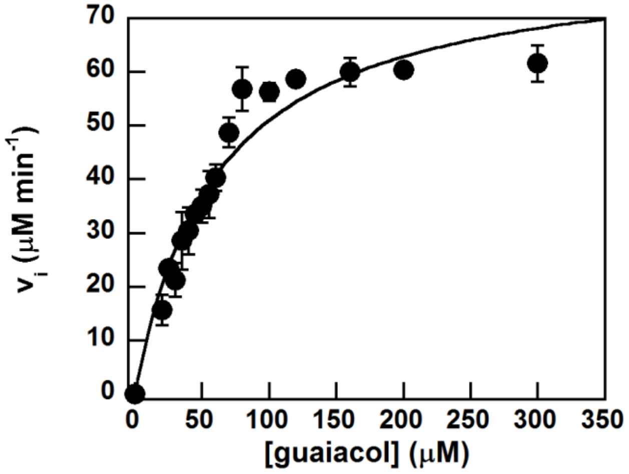

Supplementary Figure 20. Steady state reaction catalyzed by GcoA/B exhibits Michaelis-Menten kinetics. The initial velocity (v_i) of NADH oxidation was monitored in the presence of wt GcoA/B (0.2 μM each), NADH (300 μM), and ambient O_2 as a function of variable [guaiacol] (25 mM HEPES, 50 mM NaCl, pH 7.5, 25 °C). Data were fit to the Michaelis Menton curve to give the following parameters: k_{cat} = 6.8 ± 0.5 sec^{-1}, K_M(guaiacol) = 0.06 ± 0.01 mM, k_{cat}/K_M(guaiacol) = 110 ± 20 mM^{-1} sec^{-1}. Error bars represent ± 1 standard deviation from three or more measurements.

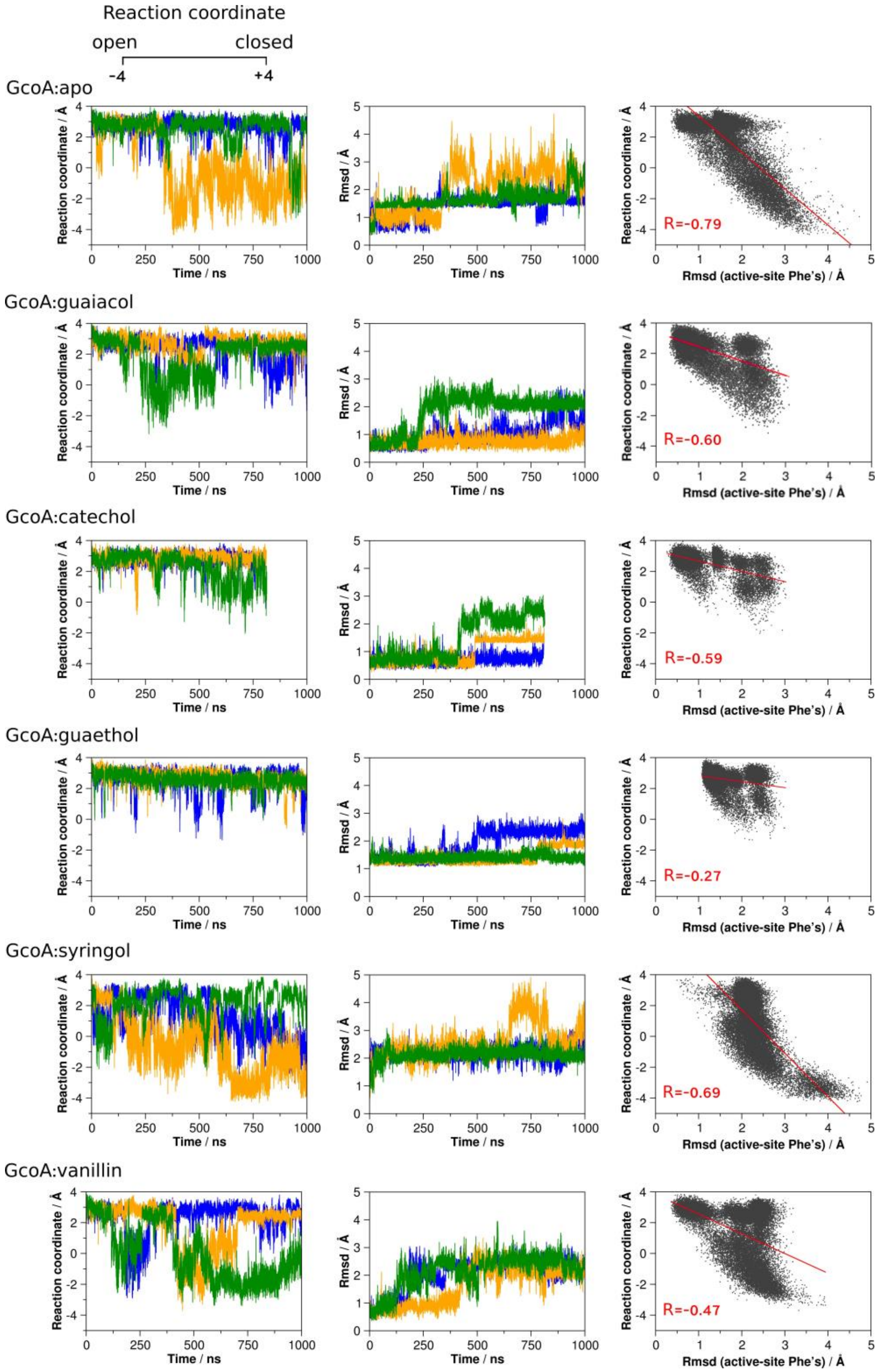

Supplementary Figure 21. Molecular dynamics of GcoA in absence of ligand and in presence of different substrates and product. The blue, orange and green colors represent different independent runs. The graphics on the left column displays the reaction coordinate employed to obtain the PMF associated to the open-close motions computed along unbiased trajectories. In several runs, we observe the transition of the reaction coordinate from positive to negative values, indicating the closed-to-open transition. The middle column displays the rmsd of residues Phe75, Phe169 and Phe395 relative to the crystal structure (computed after structural fit of the alpha-carbons of the whole enzyme), indicating the breathing motions of these residues as the rmsd increases along the unbiased MD runs. In the right column, we present scatter plots of the reaction coordinates shown on the left column and the rmsd shown in the middle column, which illustrates the correlation between the open-close motions of GcoA and the breathing motions of the Phe residues of the binding site. The R values indicate the correlation coefficient associated to a linear fit of the data (red line).

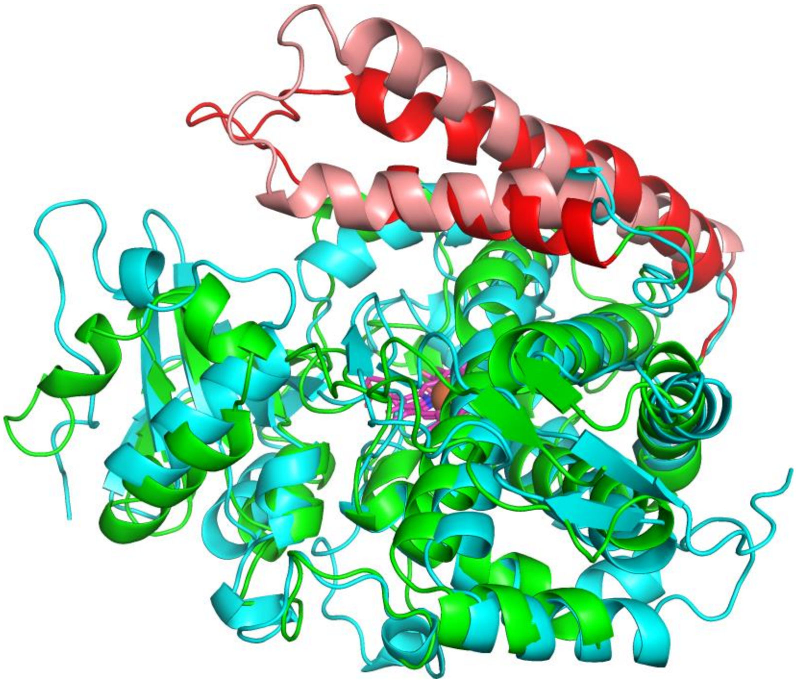

Supplementary Figure 22. **Structural comparison of the open-form of GcoA and P450-BM3.** The open structure of GcoA, obtained by MD simulation (green with F/G helices highlighted in dark red) was aligned against the crystal structure of open P450-BM3 from *Bacillus megaterium* (2HPD)[33] (cyan with F/G helices highlighted in light red). There is a good correlation of the relative positions of the F/G helices in both models of the open-form.

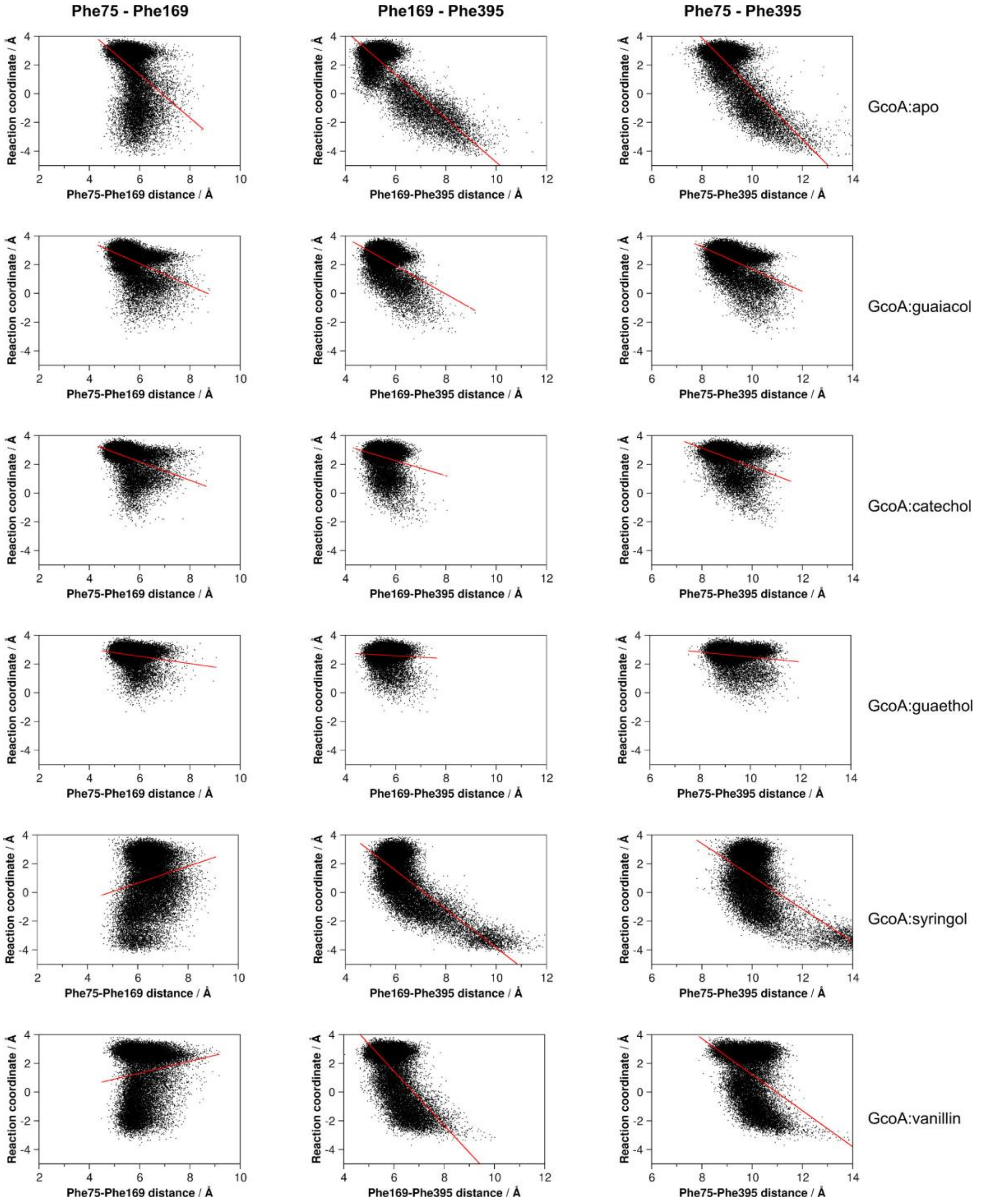

Supplementary Figure 23. Reaction coordinate as a function of distance between active-site Phe aromatic rings. Scatter plots of the reaction coordinate used to compute the PMFs as a function of the distances between the center of mass of the aromatic rings of Phe75 and Phe169, Phe169 and Phe395, and Phe75 and Phe395. These plots provide details of the RMSD metric employed to monitor the breathing motions of the active-site Phe residues along the unbiased MD runs (**Fig. S21**). The breathing motions that are coupled to the open-close motions consist mostly of relative motions of the pairs Phe75-Phe395 and Phe169-Phe395. This makes sense because Phe75 and Phe169 are located in or close to the F/G helices that undergo open-close transition. Coupling due to relative motions of Phe75-Phe169 are less evident. The RMSD metric shown in **Fig. S21** comprises all these motions in a single variable and properly represents the breathing motions in a simple format. The red lines are best-fit linear regression models and represents the correlation between the variables being analyzed.

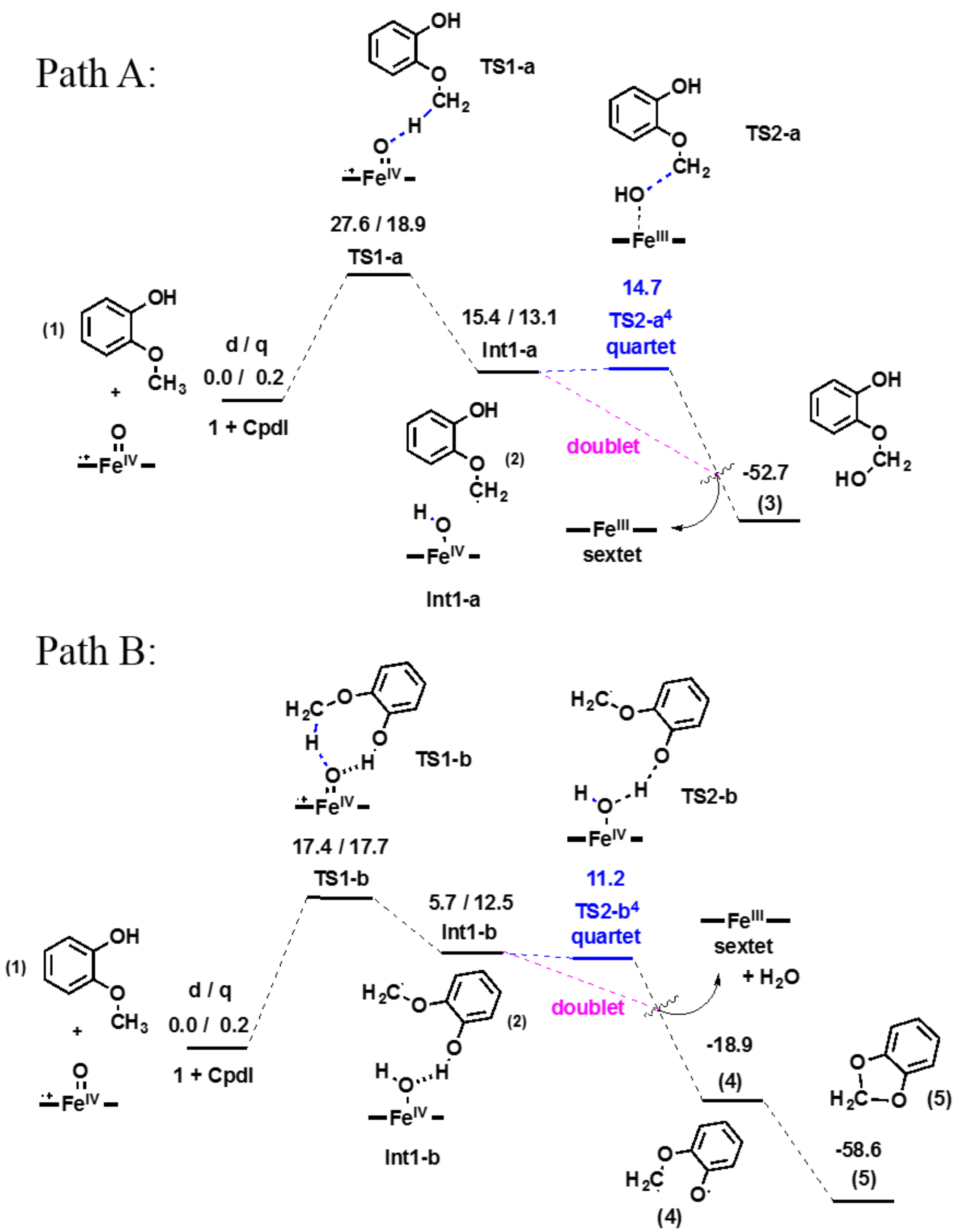

Supplementary Figure 24. **DFT computed paths for the oxidation of guaiacol.** Path **A** and path **B** for the oxidation of guaiacol at uB3LYP-D3BJ/6-311+G(d,p)+Fe(LanL2DZ)(PCM=Diethylether)//B3LYP/6-31G(d)+Fe(LanL2DZ) level, considering the two electronic states (d / q: d=doublet; q=quartet) of the Fe=O active species. Gibbs energies are given in kcal·mol^{-1}.

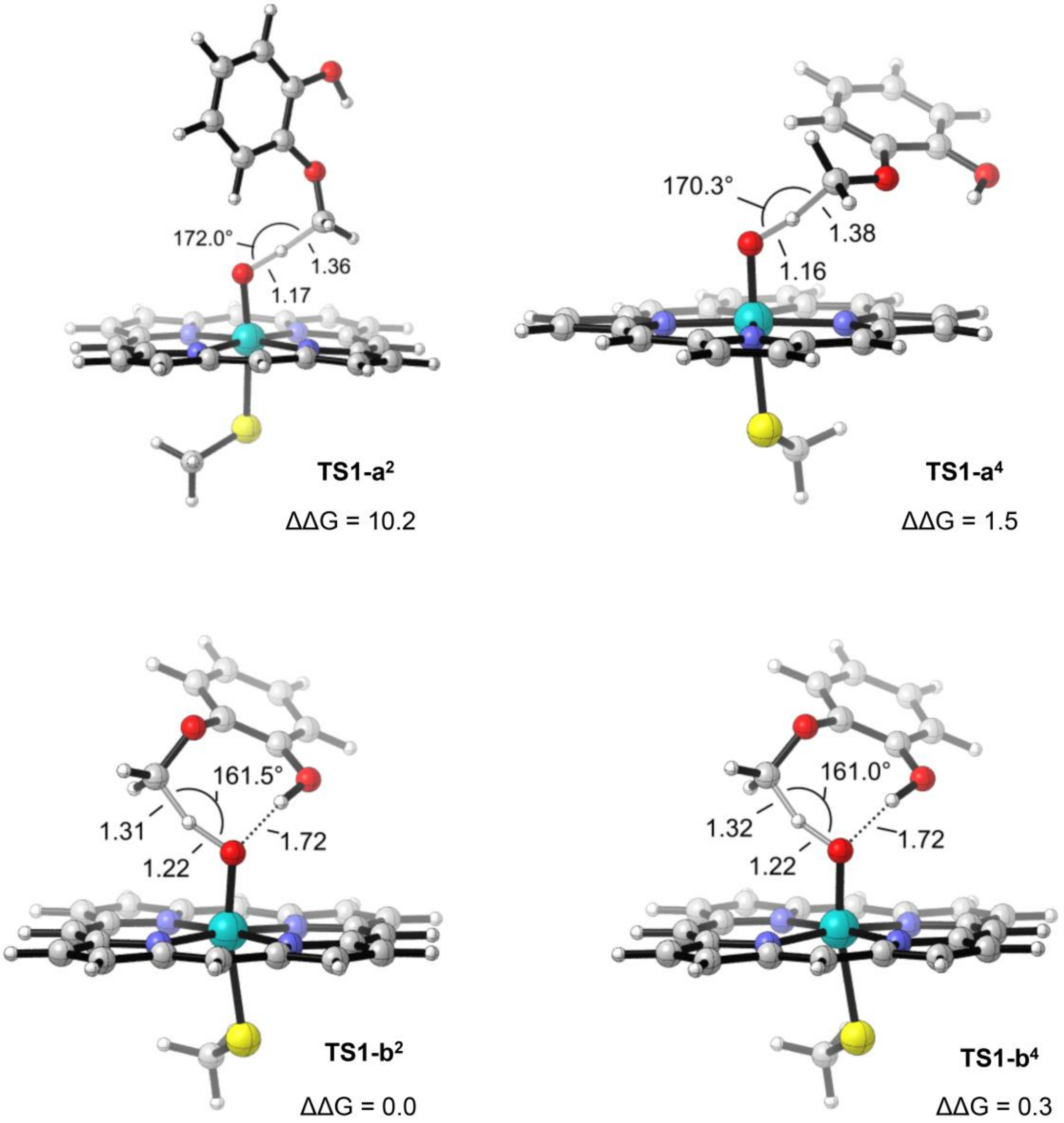

Supplementary Figure 25. DFT optimized structures of the rate limiting transition states TS1-a^2 / TS1-a^4 and TS1-b^2 / TS1-b^4. Gibbs energies are given in kcal·mol^{-1}, distances in Å, and angles in degrees.

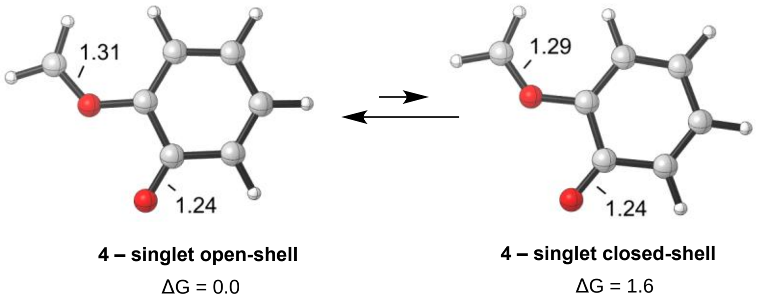

Supplementary Figure 26. Relative stabilities of biradical (singlet open-shell) / zwitterion (singlet closed-shell) intermediate 4. Computed at uB3LYP-D3BJ/6-311+G(d,p)+Fe(LanL2DZ)(PCM=Diethylether)//B3LYP/6-31G(d)+Fe(LanL2DZ) level. Gibbs energies are given in kcal·mol^{-1} and distances in Å.

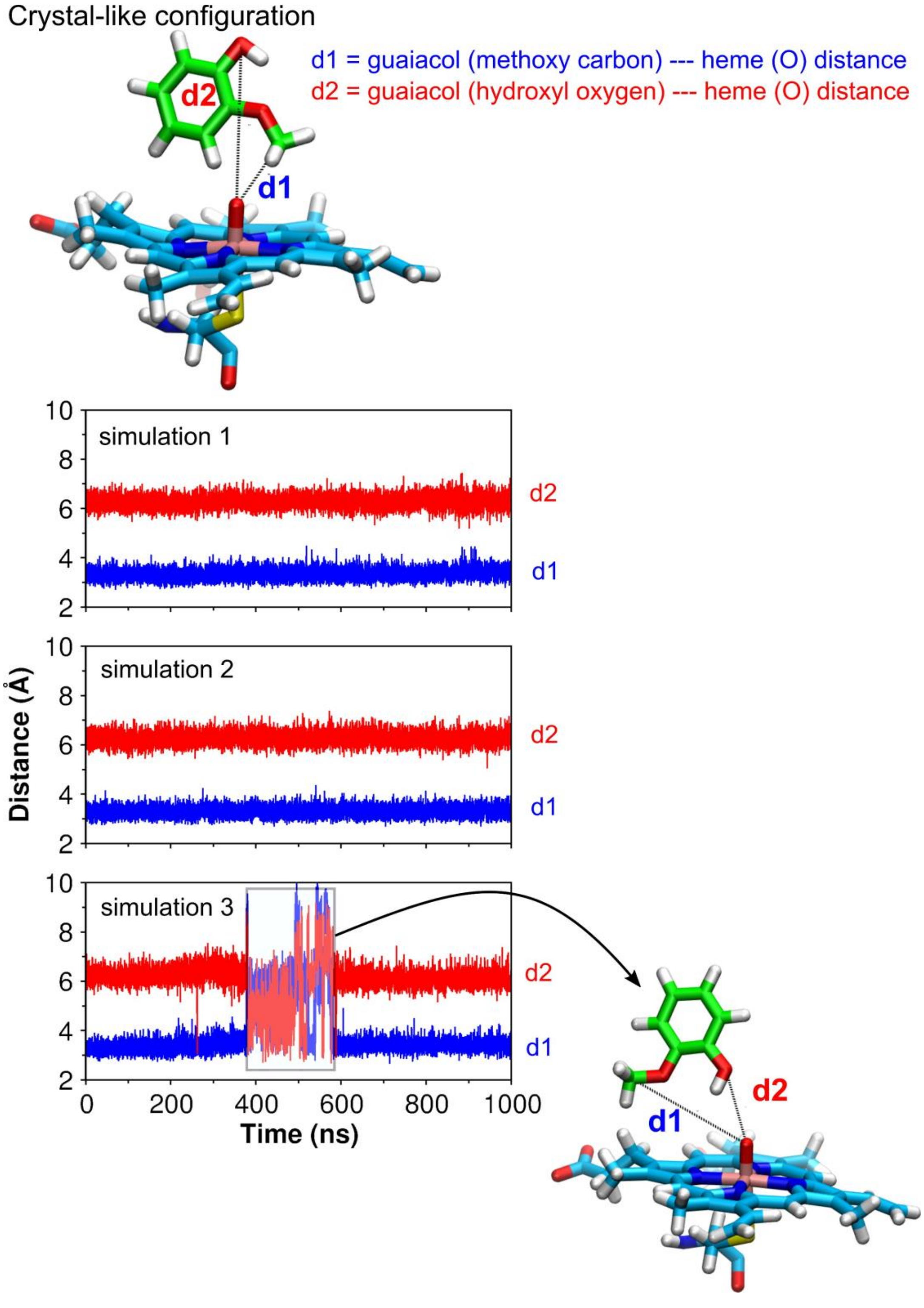

Supplementary Figure 27. Orientation of guaiacol relative to the Fe=O group. Distances d1 and d2 are computed along the three independent simulations of the GcoA:guaiacol system. During most of the simulation time, we have d1 and d2 fluctuating around ~3.5 Å and ~6.5 Å, respectively, indicating that the crystal-like conformation is maintained, therefore, favoring the path A predicted by the DFT calculations. In simulation 3, we observe a transient deviation of the guaiacol configuration around 500 ns, in which neither path A nor path B is favored. This transient configuration returns to the crystal-like configuration at 600 ns and is likely just a thermal fluctuation with no significant impact on the GcoA mechanism.

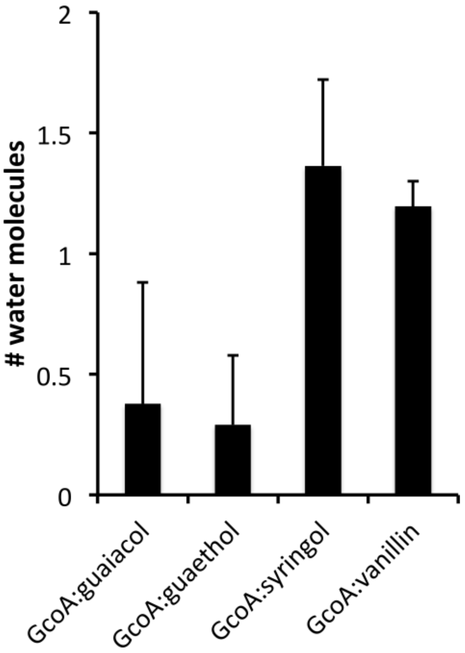

Supplementary Figure 28. Average number of water molecules around the substrates. The average number of water molecules within 3.4 Å from any atom of the substrate was computed. It can be seen that syringol and vanillin exhibit a hydration shell with, in average, higher numbers of water molecules than guaiacol and guaethol, which is associated to the higher flexibility of GcoA bound to the former substrates. The average number of water molecules lesser than 0.5 in GcoA:guaiacol and GcoA:guaethol indicates that most of the time there is no water molecule around the substrate.

Supplementary Table 1. Oligonucleotide sequences for cloning

**Gene**	**Primer**	**Sequence**
gcoa - forward	oCJ322	5’-TCGGATCTGGAAGTTCTGTTCCAGGGGCCCATGACGACGACCGAACGGC-3’
gcoa - reverse	oCJ209	5’-TCGTCAGTCAGTCACGATGCGGCCGCTCGATCACACCTCCCAGGTGACGTG-3’
gcob - forward	oCJ323	5’-TCGGATCTGGAAGTTCTGTTCCAGGGGCCCATGACGTTCGCGGTCAGCG-3’
gcob - reverse	oCJ324	5’-TCGTCAGTCAGTCACGATGCGGCCGCTCGATCACGAGGCCGGCGTG-3’

Supplementary Table 2. Crystallographic data collection and refinement statistics

	GcoA with guaiacol	GcoA with vanillin	GcoA with guaethol	GcoA with syringol	GcoB
	5NCB	5OMR	5OMS	5OMU	5OGX
**Data collection**					
Space group	$P\ 4_3\ 2_1\ 2$	$P\ 4_3\ 2_1\ 2$	$P\ 4_3\ 2_1\ 2$	$P\ 4_3\ 2_1\ 2$	$P\ 2_12_12_1$
Cell dimensions					
a, *b*, *c* (Å)	104.08, 104.08, 115.79	104.11, 104.11, 115.66	104.28, 104.28, 114.62	105.36, 105.36, 113.23	60.85, 65.90, 97.85
α, β, γ (°)	90, 90, 90	90, 90, 90	90, 90, 90	90, 90, 90	90, 90, 90
Resolution (Å)	115.75 - 1.44 (1.46 - 1.44)	47.46 - 1.68 (1.72 - 1.68)	47.46 - 1.95 (2.00 - 1.95)	47.11 - 1.95 (2.00 - 1.95)	97.85 - 1.72 (1.75 - 1.72)
R_{merge}	0.073 (0.931)	0.064 (0.651)	0.045 (0.773)	0.049 (0.854)	0.056 (0.536)
$I / \sigma I$	21.0 (2.1)	30.4 (5.2)	31.6 (3.2)	25.5 (3.3)	14.3 (2.7)
Completeness (%)	99.7 (99.6)	99.9 (99.2)	99.9 (100.0)	100 (99.9)	100.0 (100.0)
Redundancy	10.2 (8.6)	20.5 (19.5)	13.1 (12.6)	13.0 (13.4)	6.3 (6.2)
**Refinement**					
Resolution (Å)	77.39 - 1.44 (1.46 - 1.44)	47.46 - 1.68 (1.72 - 1.68)	47.46 - 1.95 (2.00 - 1.95)	47.11 - 1.95 (2.00 - 1.95)	48.92 - 1.72 (1.75 - 1.72)
No. reflections	114601 (5611)	72761 (2643)	46606 (2551)	46982 (2575)	42513 (2852)
R_{work}	0.128 (0.228)	0.147 (0.219)	0.152 (0.190)	0.148 (0.223)	0.165 (0.204)
R_{free}	0.151 (0.238)	0.169 (0.239)	0.175 (1.238)	0.178 (1.283)	0.193 (0.270)
No. atoms	3548	3806	3548	3682	2960
Protein	3193	3199	3166	3159	2553
Ligand/ion	52	54	53	54	59
Water	303	553	329	469	348
B-factors	23.6	26.8	39.5	49.7	30.1
Protein	23.8	24.6	38.9	48.4	28.4
Ligand/ion	16.7	18.4	29.4	35.6	26.0
Water	32.1	40.0	47.1	59.8	43.2
R.m.s. deviations					
Bond lengths (Å)	0.015	0.01	0.01	0.009	0.006
Bond angles (°)	1.71	2.24	2.277	2.23	1.079

*Each structure was determined from a single crystal. *Values in parentheses are for highest-resolution shell.

Supplementary Table 3. Hydrodynamics of GcoA, GcoB and GcoAB. Dynamic light scattering (DLS) and sedimentation velocity analytical ultracentrifugation (AUC SV) parameters are given for each species.

	DLS				AUC SV		
	Sequence MW (kDa)	R_h[a] (nm)	Calculated MW (kDa)	% Polydispersity[b]	$S_{20,w}$[c]	f/f_0[d]	RMSD[e]
GcoA	45.5	2.92	41.6	18.4	3.53	1.33	0.0036
GcoB	35.7	2.83	38.6	20.1	2.92	1.36	0.0026
GcoAB	81.3	3.88	80.3	20	4.37	1.38	0.0032

[a] The hydrodynamic radius (R_h) of a protein is related to the measured diffusion constant (D_t) through the Stokes-Einstein equation: $D_t = k_B T/6\pi\eta R_h$ where k_B is the Boltzmann constant, T is the temperature in Kelvin, and η is the absolute viscosity of the solvent. [b] % Polydispersity is given by $\%Pd = 100*(\mu_2/\mu_1)$ where μ_1 and μ_2 are the first and second moments of the intensity distribution. [c] $S_{20,w}^{o}$ is the Sedimentation coefficient at 20 °C in water. $S = v/\omega^2 r$ where v is the velocity of the particle and $\omega^2 r$ is the centrifugal field. [d] f/f_0 is the frictional ratio, comparing the calculated frictional coefficient ($f = RT/D.N_A$ where R is the gas constant, T is the temperature in kelvin, D is the measure diffusion coefficient and N_A is Avogadro's number) to the frictional coefficient of a maximally compact sphere. [e] The RMSD of the curve fit to the observed data generated by Sedfit.

Supplementary Table 4. Relative efficiencies of aromatic demethylases

Protein	k_{cat} (s^{-1})	K_M (mM)	k_{cat}/K_M (mM^{-1} sec^{-1})
GcoA (guaiacol)[a]	6.8 ± 0.5	0.06 ± 0.01	110 ± 20
LigM (vanillate)[b,35]	5.8 ± 0.25	0.63 ± 0.08	9.14
PODA (cryptopine)[c,36]	0.034	0.027	1.3
LigX (5,5'-dehydrodivanillate)[d,37]	6.1 ± 0.2	0.064 ± 0.005	95

[a] Reaction conditions: 0.2 µM GcoA/B, 100 µg/mL catalase and 300 µM NADH were used to *O*-demethylate 5-300 µM guaiacol in 25 mM HEPES, 50 mM NaCl, pH 7.5, 25 ºC, air (260 µM O_2).
[b] Reaction conditions: 0.076 µM LigM, was used to *O*-demethylate vanillate (0.1-5 mM) in the presence of 5 mM H_4-folate in 100 mM Tris, pH 8.0, 30 ºC, air (~260 µM O_2).
[c] Reaction conditions: 100 µg PODA, 500 µM α-KG, 500 µM iron sulfate, and 10 mM sodium ascorbate were used to *O*-demethylate cryptopine (0-500 µM) in 100 mM Tris, pH 7.4, 30 ºC, air (~260 µM O_2).
[d] Reaction conditions: 0.2 µM LigXa, 6 µM LigXc, 0.4 µM LigXd, and 500 µM NADH were used to *O*-demethylate 5,5'-dehydrodivanillate (10-500 µM) in FE22 buffer, pH 6.0, 30 ºC, air (~260 µM O_2).

Supplementary Table 5. DFT optimized geometries
Electronic energies (E), zero point energy (ZPE), free energy (G(T)), quasiharmonic corrected free energy (qh-G(T)), and electronic energy from high level single point calculation (E Single point) of all stationary points (in a.u.). Cartesian coordinates are reported in xyz format.

Structure	E (au)	ZPE (au)	G(T) (au)	qh-G(T) (au)	E Single Point (au)
1 - guaiacol	-421.981463	0.138080	-421.875943	-421.875640	-264797.165866
2 - radical intermediate	-421.316271	0.123880	-421.225317	-421.225238	-264379.751899
4 - zwitterion (singlet closed-shell)	-420.684227	0.112437	-420.603823	-420.603823	-263983.138601
4 - biradical (singlet open-shell)	-420.687517	0.110919	-420.608994	-420.608848	-263985.203105
4 - biradical (triplet)	-420.675655	0.110664	-420.598263	-420.598263	-263977.759593
3 - hemiacetal	-497.191001	0.142669	-497.082210	-497.082090	-311991.827847
H_2O	-76.407024	0.021138	-76.404206	-76.404206	-47946.095223
5 - acetal	-420.769745	0.116560	-420.684420	-420.684155	-264036.801915
Fe-Porph - doblet	-1549.967813	0.315301	-1549.702562	-1549.699826	-972618.752368
Fe-Porph - quartet	-1549.964433	0.314501	-1549.701985	-1549.698522	-972616.631387
Fe-Porph - sextet	-1549.964112	0.312990	-1549.704649	-1549.700465	-972616.429957
Fe=O-Porph - doblet	-1625.106262	0.317660	-1624.839694	-1624.837490	-1019768.805361
Fe=O-Porph - quartet	-1625.106093	0.317696	-1624.840115	-1624.837883	-1019768.699312
FeOH-Porph - singlet	-1625.728311	0.329451	-1625.448013	-1625.446500	-1020159.146707
FeOH-Porph - triplet	-1625.746377	0.327846	-1625.470763	-1625.468377	-1020170.483285
FeH_2O-Porph - doblet	-1626.387773	0.338497	-1626.101953	-1626.099515	-1020572.965047
FeH_2O-Porph - quartet	-1626.373840	0.337004	-1626.091453	-1626.088705	-1020564.221965
FeH_2O-Porph - sextet	-1626.382753	0.336628	-1626.104186	-1626.099006	-1020569.814952
TS1-a^2 - doblet	-2047.058534	0.449620	-2046.675095	-2046.665665	-1284547.653612
TS1-a^4 - quartet	-2047.059426	0.449840	-2046.674340	-2046.666688	-1284548.213350
TS1-b^2 - doblet	-2047.063575	0.449624	-2046.678611	-2046.670329	-1284550.816885
TS1-b^4 - quartet	-2047.062167	0.449525	-2046.677374	-2046.669715	-1284549.933352
Int1-a^2 - doblet	-2047.070397	0.453227	-2046.685162	-2046.674772	-1284555.097751
Int1-a^4 - quartet	-2047.070879	0.452582	-2046.687477	-2046.676979	-1284555.400210
Int1-b^2 - doblet	-2047.081556	0.452620	-2046.694959	-2046.686402	-1284562.100124
Int1-b^4 - quartet	-2047.079517	0.452580	-2046.694092	-2046.685110	-1284560.820633
TS2-a^4 - quartet	-2047.065659	0.452228	-2046.680618	-2046.671105	-1284552.124613
TS2-b^4 - quartet	-2047.074035	0.448035	-2046.690119	-2046.683622	-1284557.380629

References

1. Evans, P. Scaling and assessment of data quality. *Acta Crystallogr. D Biol. Crystallogr.* **62**, 72-82 (2006).
2. Winter, G. xia2: an expert system for macromolecular crystallography data reduction. *J. Appl. Crystallogr.* **43**, 186-190 (2010).
3. Evans, P.R. An introduction to data reduction: space-group determination, scaling and intensity statistics. *Acta Crystallogr. D Biol. Crystallogr.* **67**, 282-292 (2011).
4. Padilla, J.E. & Yeates, T.O. A statistic for local intensity differences: robustness to anisotropy and pseudo-centering and utility for detecting twinning. *Acta Crystallogr. Sect. D-Biol. Crystallogr.* **59**, 1124-1130 (2003).
5. Kabsch, W. XDS. *Acta Crystallogr. D Biol. Crystallogr.* **66**, 125-132 (2010).
6. Winn, M.D. et al. Overview of the CCP4 suite and current developments. *Acta Crystallogr. D Biol. Crystallogr.* **67**, 235-242 (2011).
7. Skubak, P. & Pannu, N.S. Automatic protein structure solution from weak X-ray data. *Nat. Commun.* **4**, 2777 (2013).
8. Sheldrick, G.M. A short history of SHELX. *Acta Crystallogr. A* **64**, 112-122 (2008).
9. Schneider, T.P.a.S., G.M. Substructure solution with SHELXD. *Acta Cryst.* **D58**, 1772-1779 (2002).
10. Abrahams, J.P. Bias reduction in phase refinement by modified interference functions introducing the γ correction. *Acta Cryst.* **D53**, 371-376 (1997).
11. Skubak, P., Waterreus, W.J. & Pannu, N.S. Multivariate phase combination improves automated crystallographic model building. *Acta Crystallogr. D. Biol. Crystallogr.* **66**, 783-788 (2010).
12. Cowtan, K. Recent developments in classical density modification. *Acta Crystallogr. D Biol. Crystallogr.* **66**, 470-478 (2010).
13. Cowtan, K. The Buccaneer software for automated model building. 1. Tracing protein chains. *Acta Crystallogr D Biol Crystallogr* **62**, 1002-1011 (2006).
14. Sheldrick, G.M. Experimental phasing with SHELXC/D/E: combining chain tracing with density modification. *Acta Crystallogr. D Biol. Crystallogr.* **66**, 479-485 (2010).
15. Murshudov, G.N. et al. REFMAC5 for the refinement of macromolecular crystal structures. *Acta Crystallogr. D Biol. Crystallogr.* **67**, 355-367 (2011).
16. Emsley, P. & Cowtan, K. Coot: model-building tools for molecular graphics. *Acta Crystallogr. D Biol. Crystallogr.* **60**, 2126-2132 (2004).
17. McCoy, A.J. et al. Phaser crystallographic software. *J. Appl. Crystallogr.* **40**, 658-674 (2007).
18. Adams, P.D. et al. PHENIX: a comprehensive Python-based system for macromolecular structure solution. *Acta Crystallogr. D Biol. Crystallogr.* **66**, 213-221 (2010).
19. Terwilliger, T.C. et al. Iterative model building, structure refinement and density modification with the PHENIX AutoBuild wizard. *Acta Crystallogr. D Biol. Crystallogr.* **64**, 61-69 (2008).
20. Afonine, P.V. et al. Towards automated crystallographic structure refinement with phenix.refine. *Acta Crystallogr. D Biol. Crystallogr.* **68**, 352-367 (2012).
21. Rocchia, W., Alexov, E. & Honig, B. Extending the applicability of the nonlinear Poisson-Boltzmann equation: Multiple dielectric constants and multivalent ions. *J. Phys. Chem. B* **105**, 6507-6514 (2001).
22. Chen, V.B. et al. MolProbity: all-atom structure validation for macromolecular crystallography. *Acta Crystallogr. D Biol. Crystallogr.* **66**, 12-21 (2010).
23. Cryle, M.J. & Schlichting, I. Structural insights from a P450 Carrier Protein complex reveal how specificity is achieved in the P450BioI ACP complex. *Proc. Natl. Acad. Sci.* **105**, 15696-15701 (2008).
24. Poulos, T.L., Finzel, B.C., Gunsalus, I.C., Wagner, G.C. & Kraut, J. The 2.6-A crystal structure of *Pseudomonas putida* cytochrome P-450. *J. Biol. Chem.* **260**, 16122-16130 (1985).
25. Huang, W.C., Ellis, J., Moody, P.C., Raven, E.L. & Roberts, G.C. Redox-linked domain movements in the catalytic cycle of cytochrome p450 reductase. *Structure* **21**, 1581-1589 (2013).
26. Tripathi, S., Li, H. & Poulos, T.L. Structural basis for effector control and redox partner recognition in cytochrome P450. *Science* **340**, 1227-1230 (2013).
27. Karlsson, A. et al. X-ray crystal structure of benzoate 1, 2-dioxygenase reductase from *Acinetobacter* sp. strain ADP1. *J. Mol. Biol.* **318**, 261-272 (2002).
28. Wang, M. et al. Three-dimensional structure of NADPH–cytochrome P450 reductase: prototype for FMN-and FAD-containing enzymes. *Proc. Natl. Acad. Sci.* **94**, 8411-8416 (1997).
29. Huang, W.-C., Ellis, J., Moody, Peter C.E., Raven, Emma L. & Roberts, Gordon C.K. Redox-linked domain movements in the catalytic cycle of cytochrome P450 reductase. *Structure* **21**, 1581-1589 (2013).
30. Rupp, H., Rao, K.K., Hall, D.O. & Cammack, R. Electron spin relaxation of iron-sulphur proteins studied by microwave power saturation. *Biochim. Biophys. Acta* **537**, 255-269 (1978).
31. Eby, D.M., Beharry, Z.M., Coulter, E.D., Kurtz, D.M., Jr. & Neidle, E.L. Characterization and evolution of anthranilate 1,2-dioxygenase from *Acinetobacter* sp. strain ADP1. *J. Bacteriol.* **183**, 109-118 (2001).
32. Guengerich, F.P., Martin, M.V., Sohl, C.D. & Cheng, Q. Measurement of cytochrome P450 and NADPH-cytochrome P450 reductase. *Nat. Protocols* **4**, 1245-1251 (2009).
33. Ravichandran, K., Boddupalli, S., Hasermann, C., Peterson, J. & Deisenhofer, J. Crystal structure of hemoprotein domain of P450BM-3, a prototype for microsomal P450's. *Science* **261**, 731-736 (1993).
34. Engh, R.A. & Huber, R. Accurate bond and angle parameters for X-ray protein-structure refinement. *Acta Crystallogr. A* **47**, 392-400 (1991).

35. Kohler, A.C., Mills, M.J., Adams, P.D., Simmons, B.A. & Sale, K.L. Structure of aryl *O*-demethylase offers molecular insight into a catalytic tyrosine-dependent mechanism. *Proc. Natl. Acad. Sci.* **114**, E3205-E3214 (2017).
36. Farrow, S.C. & Facchini, P.J. Dioxygenases catalyze O-demethylation and O,O-demethylenation with widespread roles in benzylisoquinoline alkaloid metabolism in opium poppy. *J. Biol. Chem.* **288**, 28997-29012 (2013).
37. Yoshikata, T. et al. Three-component *O*-demethylase system essential for catabolism of a lignin-derived biphenyl compound in *Sphingobium* sp. strain SYK-6. *Appl. Env. Microbiol.* **80**, 7142-7153 (2014).
