# Supplementary material for: A promiscuous cytochrome P450 aromatic O-demethylase for lignin bioconversion
Source: Nat Commun. 2018 Jun 27;9:2487. doi: 10.1038/s41467-018-04878-2 (PMC6021390; doi:10.1038/s41467-018-04878-2)
Supplement: Supplementary file 2 — Description of Additional Supplementary Files [file 41467_2018_4878_MOESM2_ESM.pdf]

Description of Additional Supplementary Files

File Name: Supplementary Movie 1.
Description: A snapshot of the molecular dynamics simulation of the apo form of GcoA starting from a closed state.

File Name: Supplementary Movie 2
Description: A snapshot of the molecular dynamics simulation of the guaiacol-bound form of GcoA starting from an open state.

File Name: Supplementary Data 1
Description: DFT Cartesian coordinates of optimized structures.
