# Supplementary material for: A promiscuous cytochrome P450 aromatic O-demethylase for lignin bioconversion
Source: Nat Commun. 2018 Jun 27;9:2487. doi: 10.1038/s41467-018-04878-2 (PMC6021390; doi:10.1038/s41467-018-04878-2)
Supplement: Supplementary file 3 — Supplementary Data 1 [file 41467_2018_4878_MOESM3_ESM.pdf]

Supplementary Information

A promiscuous cytochrome P450 aromatic *O*-demethylase for lignin bioconversion

Sam J. B. Mallinson, et al.

DFT Cartesian coordinates of optimized structures

```
5 - acetal
C   -0.03267    0.97449    1.42848
C   -0.01016   -0.19668    0.69676
C   -0.01016   -0.19668   -0.69676
C   -0.03267    0.97449   -1.42848
C   -0.05285    2.17773   -0.69729
C   -0.05285    2.17773    0.69729
H   -0.03552    0.96117    2.51307
H   -0.03552    0.96117   -2.51307
H   -0.06680    3.12110   -1.23505
H   -0.06680    3.12110    1.23505
O   -0.01016   -1.49283   -1.15873
O   -0.01016   -1.49283    1.15873
C    0.15790   -2.31578    0.00000
H    1.16919   -2.74736    0.00000
H   -0.60125   -3.10368    0.00000
```

```
1 - guaiacol
C   -0.02002   -1.39717   -0.00004
C    0.44007   -0.08345   -0.00005
C   -0.47387    0.99097   -0.00002
C   -1.84064    0.73399    0.00003
C   -2.30272   -0.58676    0.00005
C   -1.39959   -1.64667   -0.00002
H    0.67931   -2.22635   -0.00001
H   -2.52610    1.57582    0.00009
H   -3.37171   -0.77940    0.00005
H   -1.75515   -2.67283   -0.00005
O   -0.01576    2.27518    0.00001
H    0.95637    2.22665    0.00020
O    1.75764    0.31588   -0.00017
C    2.76033   -0.68664    0.00012
H    2.69204   -1.31907   -0.89462
H    3.71674   -0.16047   -0.00005
H    2.69202   -1.31859    0.89521
```

```
2 - radical intermediate
C   -0.07992   -1.42343   -0.11651
C    0.46726   -0.14379   -0.09464
C   -0.35439    0.99435   -0.00330
C   -1.73596    0.83147    0.07894
C   -2.28829   -0.45182    0.06809
C   -1.46736   -1.57597   -0.03069
H    0.57102   -2.28482   -0.22383
H   -2.35820    1.71762    0.15460
H   -3.36622   -0.56893    0.13292
H   -1.89899   -2.57208   -0.05075
O    0.18660    2.24396    0.01146
H    1.15237    2.14054   -0.04851
O    1.82002    0.13802   -0.18444
C    2.73145   -0.81613    0.16726
H    3.75171   -0.47893    0.03589
H    2.45854   -1.49741    0.96856
```

```
4 - zwitterion (singlet closed-shell)
C   -0.00195   -1.41603   -0.00036
C    0.49243   -0.09345    0.00002
C   -0.36820    1.10013   -0.00001
C   -1.79789    0.78142    0.00088
C   -2.25698   -0.50340    0.00040
C   -1.36047   -1.62110   -0.00044
H    0.67504   -2.26573   -0.00128
H   -2.47379    1.63127    0.00145
H   -3.32741   -0.69582    0.00036
H   -1.75839   -2.63104   -0.00133
O    0.07541    2.25602   -0.00093
```

O	1.80897	0.19756	0.00019
C	2.83906	-0.57317	0.00029
H	3.79108	-0.06098	0.00147
H	2.74229	-1.65284	0.00063

4 - biradical (singlet open-shell)

C	0.02153	-1.40128	0.00007
C	0.50411	-0.08431	0.00007
C	-0.38552	1.08715	-0.00001
C	-1.80849	0.76325	-0.00005
C	-2.25851	-0.52870	-0.00007
C	-1.34508	-1.62245	0.00001
H	0.70109	-2.24770	0.00016
H	-2.48864	1.60956	-0.00006
H	-3.32585	-0.73498	-0.00012
H	-1.72386	-2.64005	0.00007
O	0.04334	2.25392	-0.00001
O	1.81594	0.23795	0.00021
C	2.84493	-0.57779	-0.00033
H	3.80554	-0.08420	0.00017
H	2.71967	-1.65275	-0.00001

4 - biradical (singlet triplet)

C	0.07336	-1.36757	-0.09942
C	0.52463	-0.05860	-0.07705
C	-0.41369	1.06991	-0.00036
C	-1.81992	0.72805	0.06415
C	-2.24734	-0.58204	0.05130
C	-1.30606	-1.62851	-0.02962
H	0.77963	-2.18580	-0.19215
H	-2.51320	1.56133	0.12369
H	-3.30680	-0.81627	0.10311
H	-1.64716	-2.65979	-0.04275
O	-0.00839	2.25490	0.01030
O	1.83198	0.30182	-0.14508
C	2.80168	-0.62764	0.12765
H	3.79338	-0.26050	-0.10344
H	2.62943	-1.29435	0.96990

3 - hemiacetal

C	-0.87157	-1.47905	-0.17713
C	0.02840	-0.43854	-0.39186
C	-0.39295	0.89249	-0.22335
C	-1.70982	1.15661	0.15567
C	-2.60501	0.10580	0.37194
C	-2.18974	-1.21440	0.20517
H	-0.52348	-2.49677	-0.32827
H	-2.03448	2.18803	0.28414
H	-3.62664	0.32750	0.66696
H	-2.88332	-2.03407	0.36690
O	0.52619	1.88613	-0.45710
H	0.10205	2.74330	-0.29542
O	1.30976	-0.69626	-0.83673
C	2.30933	-0.82950	0.17150
H	2.09777	-1.68893	0.81996
H	3.22728	-1.00839	-0.40386
O	2.39859	0.27737	1.01444
H	2.35267	1.07113	0.45305

Fe-Porph doblet

Fe	0.06756	-0.00018	-0.11909
N	1.49338	-1.40863	-0.27288
N	-1.33983	-1.40176	-0.38362
N	1.49965	1.40218	-0.27281
N	-1.33368	1.40749	-0.38386
C	2.85944	-1.23356	-0.28130
C	-2.69977	-1.22185	-0.52603
C	1.30068	-2.77096	-0.21616
C	-1.15651	-2.76579	-0.31993
C	2.86486	1.22116	-0.28116

C	-2.69436	1.23351	-0.52613
C	1.31283	2.76529	-0.21613
C	-1.14438	2.77066	-0.31997
C	3.53468	-2.50672	-0.24523
C	-3.37768	-2.49264	-0.55985
C	2.56679	-3.46127	-0.19777
C	-2.42030	-3.45078	-0.42421
C	3.54567	2.49137	-0.24506
C	-3.36674	2.50724	-0.55983
C	2.58193	3.45012	-0.19766
C	-2.40516	3.46118	-0.42417
H	4.60974	-2.63382	-0.25389
H	-4.44698	-2.61750	-0.67445
H	2.68047	-4.53736	-0.16268
H	-2.54002	-4.52669	-0.40779
H	4.62128	2.61381	-0.25379
H	-4.43550	2.63676	-0.67425
H	2.70030	4.52571	-0.16265
H	-2.52017	4.53760	-0.40750
C	3.50575	-0.00763	-0.29595
C	-3.33646	0.00721	-0.60251
C	0.08410	3.40603	-0.21985
C	0.06922	-3.40642	-0.21980
H	4.59139	-0.01004	-0.29659
H	-4.41667	0.00961	-0.71189
H	0.08453	4.49100	-0.17893
H	0.06501	-4.49139	-0.17886
S	0.07730	0.00149	2.09439
C	-1.65983	0.00127	2.66557
H	-2.19271	-0.89225	2.33140
H	-1.62541	0.00313	3.76044
H	-2.19398	0.89290	2.32850

Fe-Porph-H2O - doblet

Fe	0.05972	0.00013	-0.10196
N	1.52154	-1.39355	-0.16026
N	-1.35210	-1.42758	-0.24699
N	1.48798	1.42930	-0.14419
N	-1.38396	1.39301	-0.26691
C	2.88049	-1.19649	-0.11935
C	-2.71273	-1.26488	-0.35222
C	1.33516	-2.75365	-0.11963
C	-1.13438	-2.78246	-0.20834
C	2.84999	1.26525	-0.10808
C	-2.74203	1.19720	-0.36684
C	1.26887	2.78338	-0.11069
C	-1.19962	2.75389	-0.22468
C	3.56891	-2.46469	-0.07150
C	-3.36949	-2.55028	-0.37862
C	2.61021	-3.43062	-0.06861
C	-2.39038	-3.49155	-0.28494
C	3.50874	2.55002	-0.06016
C	-3.42848	2.46610	-0.39609
C	2.52745	3.49217	-0.05877
C	-2.47205	3.43125	-0.30342
H	4.64489	-2.58133	-0.03836
H	-4.43967	-2.69494	-0.45811
H	2.73599	-4.50556	-0.03417
H	-2.49058	-4.56969	-0.27351
H	4.58169	2.69211	-0.02841
H	-4.50190	2.58524	-0.47434
H	2.62670	4.56997	-0.02729
H	-2.59898	4.50658	-0.29181
C	3.50881	0.04236	-0.10607
C	-3.36883	-0.04179	-0.41789
C	0.02654	3.40280	-0.14383
C	0.10743	-3.40215	-0.13942
H	4.59388	0.05546	-0.06899
H	-4.45176	-0.05500	-0.49653
H	0.01191	4.48831	-0.11591
H	0.11893	-4.48766	-0.11005
O	0.14625	-0.00294	-2.28515
S	0.09768	-0.00434	2.11918
C	-1.62246	0.00586	2.73814
H	-2.17234	-0.87980	2.40992
H	-1.56172	-0.00070	3.83151

```
H   -2.15871    0.90343    2.41984
H    0.93595   -0.21781   -2.80169
H   -0.58708    0.20225   -2.88235
```

Fe-Porph-H2O - quartet

```
Fe    -0.06136   -0.03810   -0.09428
N   -1.43774    1.55805   -0.27318
N    1.48146    1.33583   -0.31130
N   -1.61946   -1.37160   -0.14705
N    1.30663   -1.59121   -0.30277
C   -2.79862    1.43805   -0.14916
C    2.82243    1.03360   -0.34278
C   -1.13645    2.89441   -0.22442
C    1.36742    2.70541   -0.28024
C   -2.95307   -1.06955   -0.07798
C    2.66832   -1.47556   -0.35372
C   -1.49887   -2.73479   -0.12598
C    1.00228   -2.92315   -0.27051
C   -3.38792    2.75699   -0.06245
C    3.58612    2.26280   -0.34096
C   -2.36119    3.65690   -0.10876
C    2.69038    3.29230   -0.30112
C   -3.71722   -2.30099   -0.01512
C    3.25953   -2.79882   -0.36917
C   -2.82097   -3.32707   -0.04637
C    2.22932   -3.69309   -0.31873
H   -4.44709    2.96081    0.03506
H    4.66720    2.32152   -0.36882
H   -2.42067    4.73701   -0.05562
H    2.89783    4.35522   -0.29034
H   -4.79718   -2.35923    0.03880
H    4.32130   -3.00770   -0.41257
H   -3.02353   -4.39056   -0.02143
H    2.28704   -4.77452   -0.31138
C   -3.49236    0.22278   -0.08097
C    3.36351   -0.25874   -0.37336
C   -0.29449   -3.44546   -0.19001
C    0.16220    3.41845   -0.24656
H   -4.57403    0.29059   -0.00129
H    4.44791   -0.32247   -0.40747
H   -0.37668   -4.52886   -0.16938
H    0.24693    4.50141   -0.21462
O   -0.14776    0.06348   -2.41318
S   -0.09044   -0.12323    2.23555
C    1.55154    0.40310    2.83774
H    1.74297    1.45915    2.63440
H    1.54745    0.24288    3.92223
H    2.34608   -0.20861    2.40259
H   -0.86895    0.71811   -2.39979
H    0.66033    0.59715   -2.50868
```

Fe-Porph-H2O - sextet

```
Fe   0.05151    0.04549    0.21331
N    1.60970   -1.32867   -0.14500
N   -1.28472   -1.49791   -0.30954
N    1.44818    1.55043   -0.15651
N   -1.45565    1.38174   -0.31609
C    2.95729   -1.05399   -0.09240
C   -2.65144   -1.38131   -0.43323
C    1.48327   -2.70027   -0.12109
C   -1.00248   -2.84592   -0.27295
C    2.81471    1.43201   -0.13723
C   -2.79234    1.10586   -0.47118
C    1.16517    2.89209   -0.22314
C   -1.32133    2.74799   -0.36756
C    3.70198   -2.28921   -0.04367
C   -3.24641   -2.69519   -0.48084
C    2.79145   -3.30592   -0.05871
C   -2.22827   -3.59934   -0.37882
C    3.41744    2.74535   -0.18311
C   -3.52952    2.34091   -0.60738
C    2.39730    3.64813   -0.23518
C   -2.62018    3.35580   -0.54167
H    4.78167   -2.36030   -0.00484
H   -4.30612   -2.89218   -0.58419
```

H	2.98021	-4.37182	-0.03705
H	-2.29247	-4.68016	-0.38419
H	4.48275	2.93865	-0.18445
H	-4.60083	2.41124	-0.74782
H	2.46124	4.72758	-0.28871
H	-2.80034	4.42065	-0.61843
C	3.51437	0.22568	-0.09254
C	-3.34816	-0.17381	-0.50901
C	-0.11335	3.44394	-0.30343
C	0.27592	-3.39742	-0.17519
H	4.59825	0.28771	-0.06462
H	-4.42638	-0.23703	-0.62331
H	-0.17318	4.52705	-0.35804
H	0.33840	-4.48158	-0.15863
O	0.29051	-0.53890	-2.93771
S	-0.01315	0.04015	2.54115
C	-1.79790	0.02382	2.97311
H	-2.29411	-0.84723	2.53753
H	-1.88190	-0.02350	4.06207
H	-2.28817	0.93260	2.61475
H	1.02146	-0.80420	-2.35629
H	-0.48110	-0.91579	-2.48536

Int1-a doblet

Fe	1.21415	0.08091	0.05706
N	2.05286	0.57948	-1.70280
N	1.53395	-1.89434	-0.36816
N	0.91425	2.02398	0.48061
N	0.55520	-0.41863	1.88967
C	2.19467	1.84433	-2.21353
C	1.21510	-2.96200	0.42447
C	2.48090	-0.28057	-2.68543
C	2.02329	-2.41609	-1.53488
C	1.21771	3.09596	-0.32099
C	0.36651	-1.69070	2.37165
C	0.41555	2.55366	1.64558
C	0.08785	0.43104	2.86190
C	2.75749	1.78515	-3.54071
C	1.51984	-4.20188	-0.25821
C	2.93123	0.46729	-3.83416
C	2.01973	-3.86201	-1.47612
C	0.86709	4.33146	0.34009
C	-0.20916	-1.63972	3.69276
C	0.36988	3.99511	1.56166
C	-0.37941	-0.32275	3.99889
H	2.97281	2.64807	-4.15797
H	1.36163	-5.19004	0.15505
H	3.32041	0.02441	-4.74211
H	2.35906	-4.51278	-2.27201
H	1.00359	5.31801	-0.08449
H	-0.45221	-2.50751	4.29282
H	0.01449	4.64815	2.34882
H	-0.79222	0.11213	4.90037
C	1.81599	3.01805	-1.57277
C	0.67723	-2.87145	1.70312
C	0.03223	1.81716	2.75942
C	2.46608	-1.66811	-2.61742
H	1.99710	3.95133	-2.09727
H	0.46898	-3.80182	2.22319
H	-0.35041	2.36560	3.61496
H	2.83046	-2.21043	-3.48457
O	-0.39802	-0.12692	-0.68367
S	3.51286	0.03532	0.76531
C	3.66234	-0.88894	2.32390
H	3.32345	-1.91959	2.16882
H	4.71308	-0.90416	2.62591
H	3.04800	-0.43899	3.10806
H	-0.87915	0.71949	-0.57963
C	-2.80850	1.67834	0.27653
O	-3.96485	1.50975	-0.41679
H	-2.52356	2.71878	0.36946
H	-2.56527	0.94848	1.04242
C	-4.47245	0.22843	-0.60451
C	-3.69636	-0.92625	-0.65556
C	-5.86661	0.18568	-0.77997
C	-4.33253	-2.15351	-0.86844

```
H   -2.61453   -0.86781   -0.56459
C   -6.48626   -1.04455   -0.99289
O   -6.61310    1.32546   -0.73653
C   -5.71770   -2.21022   -1.03295
H   -3.73545   -3.05915   -0.91689
H   -7.56395   -1.06732   -1.12084
H   -5.99864    2.06447   -0.58514
H   -6.20766   -3.16536   -1.20065
```

```
Int1-b  doblet
Fe   1.02938   -0.04568    0.18236
N   -0.03190   -1.37481    1.28241
N   -0.07727    1.47287    0.93653
N    2.21247   -1.54462   -0.43990
N    2.12377    1.29535   -0.84472
C    0.19599   -2.72431    1.41607
C    0.04139    2.81248    0.65846
C   -1.14675   -1.10509    2.03163
C   -1.19031    1.34985    1.73862
C    2.15102   -2.86691   -0.08759
C    1.94233    2.64889   -0.91266
C    3.23393   -1.44083   -1.34613
C    3.14616    1.00647   -1.71195
C   -0.80414   -3.31851    2.26917
C   -0.99357    3.55751    1.33540
C   -1.64456   -2.31474    2.64246
C   -1.76082    2.65051    1.99694
C    3.16256   -3.62007   -0.78653
C    2.88177    3.23845   -1.83778
C    3.83209   -2.73338   -1.57553
C    3.62863    2.21609   -2.33758
H   -0.84693   -4.36756    2.53286
H   -1.11452    4.63190    1.28163
H   -2.51819   -2.36851    3.27932
H   -2.64106    2.82547    2.60198
H    3.32239   -4.68569   -0.68244
H    2.94303    4.29458   -2.06743
H    4.65751   -2.92092   -2.25047
H    4.43199    2.25874   -3.06204
C    1.22541   -3.41887    0.79555
C    0.98860    3.36597   -0.19282
C    3.66363   -0.26062   -1.94718
C   -1.70096    0.15796    2.22681
H    1.29571   -4.48524    0.98813
H    0.95870    4.44144   -0.33960
H    4.48447   -0.33730   -2.65415
H   -2.59585    0.21613    2.83848
O   -0.09792   -0.21776   -1.24042
S    2.24997    0.27903    2.25767
C    3.19245    1.83068    2.16223
H    2.50744    2.67543    2.02261
H    3.73300    1.97085    3.10260
H    3.89288    1.81446    1.32289
H   -0.46937   -1.11541   -1.22099
C   -2.94033   -2.58718   -2.41962
O   -2.66838   -1.43515   -1.74692
H   -2.04175   -3.12574   -2.69498
H   -3.82100   -2.60034   -3.05633
C   -3.71861   -0.62439   -1.34514
C   -4.98864   -1.13259   -1.07008
C   -3.43885    0.75558   -1.21145
C   -6.02178   -0.27523   -0.68846
H   -5.15364   -2.20295   -1.14452
C   -4.49544    1.59701   -0.82943
O   -2.24354    1.31385   -1.46597
C   -5.77019    1.09402   -0.57730
H   -7.00779   -0.67813   -0.47634
H   -4.27872    2.65736   -0.74228
H   -1.47199    0.66933   -1.44776
H   -6.56641    1.77388   -0.28554
```

```
Int1-b quartet
Fe   1.04216    0.03681   -0.11648
N    0.63250    1.85240   -0.89727
N    0.00710   -0.84532   -1.59637
```

N	2.23581	0.92082	1.24734
N	1.51738	-1.78837	0.60975
C	1.12769	3.07179	-0.49210
C	-0.18442	-2.19535	-1.79749
C	-0.22219	2.11985	-1.93355
C	-0.76253	-0.20979	-2.54904
C	2.52195	2.25221	1.37504
C	1.10183	-3.00387	0.15116
C	2.87636	0.29410	2.28146
C	2.23943	-2.03964	1.74850
C	0.55920	4.13157	-1.28890
C	-1.05675	-2.41163	-2.92571
C	-0.28796	3.54122	-2.17717
C	-1.42121	-1.18426	-3.38389
C	3.37532	2.47657	2.51509
C	1.57611	-4.06149	1.01359
C	3.58954	1.25678	3.08479
C	2.28108	-3.46046	2.01079
H	0.78839	5.18313	-1.17179
H	-1.36134	-3.38441	-3.28982
H	-0.89444	4.00801	-2.94281
H	-2.08240	-0.94161	-4.20586
H	3.74423	3.44382	2.83158
H	1.38061	-5.11576	0.86499
H	4.17406	1.01640	3.96373
H	2.78730	-3.91932	2.85056
C	2.02540	3.25930	0.54887
C	0.33018	-3.20169	-0.99432
C	2.87118	-1.07803	2.52481
C	-0.89179	1.16132	-2.69026
H	2.33757	4.27734	0.76177
H	0.07398	-4.22458	-1.25300
H	3.42234	-1.42622	3.39345
H	-1.54444	1.51966	-3.48030
O	-0.38043	0.07305	1.01023
S	2.80556	-0.02378	-1.82373
C	3.43418	-1.72164	-1.98256
H	2.62334	-2.38639	-2.30472
H	4.21995	-1.73648	-2.74319
H	3.82265	-2.09035	-1.02961
H	-0.61213	1.00279	1.18529
C	-2.63081	2.49258	1.10396
O	-2.96468	1.38611	0.37953
H	-2.12380	3.25182	0.52056
H	-3.23640	2.73963	1.97002
C	-4.00554	0.57540	0.81264
C	-5.26171	1.10059	1.11330
C	-3.76088	-0.81346	0.86218
C	-6.31203	0.25066	1.46254
H	-5.41292	2.17418	1.04265
C	-4.83446	-1.65118	1.20180
O	-2.56925	-1.37046	0.58152
C	-6.09180	-1.12855	1.49797
H	-7.29068	0.66269	1.69056
H	-4.64421	-2.71961	1.23995
H	-1.80151	-0.73204	0.67545
H	-6.90190	-1.80292	1.76317

Int1-a quartet

Fe	-1.22696	0.04207	0.07463
N	-2.02749	1.27723	-1.30803
N	-0.31708	1.60558	0.95949
N	-2.30533	-1.47670	-0.69433
N	-0.49991	-1.17158	1.52065
C	-2.95130	0.96441	-2.27930
C	0.48581	1.58247	2.07904
C	-1.74079	2.60658	-1.47774
C	-0.26098	2.89691	0.47942
C	-3.18601	-1.43507	-1.74120
C	0.34606	-0.84204	2.53926
C	-2.25003	-2.78584	-0.29674
C	-0.67649	-2.52941	1.59890
C	-3.24337	2.12558	-3.08483
C	1.02574	2.89690	2.33045
C	-2.48405	3.14278	-2.59373

C	0.57025	3.70754	1.33626
C	-3.70894	-2.75148	-2.01110
C	0.71561	-2.01851	3.29300
C	-3.12047	-3.59395	-1.11586
C	0.08129	-3.06953	2.70544
H	-3.93884	2.14248	-3.91425
H	1.68259	3.14520	3.15431
H	-2.43086	4.16915	-2.93377
H	0.77153	4.75983	1.18076
H	-4.42336	-2.98584	-2.78990
H	1.37683	-2.02207	4.15010
H	-3.25564	-4.66249	-1.00635
H	0.11173	-4.11584	2.98176
C	-3.50762	-0.29224	-2.47139
C	0.78921	0.45030	2.82126
C	-1.48793	-3.28066	0.75942
C	-0.89900	3.35735	-0.66137
H	-4.22784	-0.40184	-3.27659
H	1.45851	0.57470	3.66718
H	-1.54446	-4.34842	0.94978
H	-0.75953	4.40254	-0.92028
O	0.21142	-0.34844	-0.93961
S	-3.08196	0.84831	1.49499
C	-2.78486	0.35255	3.21745
H	-1.85608	0.81386	3.57497
H	-3.61227	0.70890	3.83747
H	-2.68439	-0.73242	3.30444
H	-0.07040	-0.28718	-1.86910
C	2.75065	-2.25130	-1.88691
O	4.05343	-1.89240	-1.68548
H	2.66695	-3.27249	-2.23647
H	1.96265	-1.75765	-1.32878
C	4.40277	-0.66735	-1.14965
C	3.52178	0.34702	-0.78314
C	5.79468	-0.50938	-0.99756
C	4.04250	1.53297	-0.24970
H	2.44868	0.22701	-0.90053
C	6.29792	0.67548	-0.46770
O	6.64687	-1.51000	-1.36411
C	5.41888	1.69624	-0.09327
H	3.35852	2.32534	0.04079
H	7.37305	0.77895	-0.35757
H	6.09482	-2.23294	-1.71032
H	5.81713	2.61951	0.31900

TS2-b quartet

Fe	-0.97320	0.12506	0.20978
N	-1.98826	1.69201	-0.55555
N	0.34569	1.40719	1.02127
N	-2.30313	-1.17828	-0.58726
N	0.04623	-1.44682	0.96966
C	-3.10665	1.61783	-1.34321
C	1.46550	1.08035	1.73708
C	-1.71142	3.01670	-0.41431
C	0.31200	2.78500	0.98044
C	-3.37917	-0.84750	-1.36501
C	1.21536	-1.38142	1.68579
C	-2.30827	-2.54608	-0.49503
C	-0.26952	-2.78334	0.88193
C	-3.54258	2.94517	-1.71665
C	2.15590	2.27632	2.16471
C	-2.67502	3.81715	-1.13479
C	1.43623	3.33221	1.70160
C	-4.08740	-2.03653	-1.77266
C	1.64564	-2.70706	2.06237
C	-3.42161	-3.09478	-1.22873
C	0.72064	-3.57670	1.56962
H	-4.40046	3.16097	-2.34070
H	3.07061	2.28404	2.74322
H	-2.66976	4.89856	-1.18142
H	1.63606	4.38949	1.81963
H	-4.97419	-2.04552	-2.39360
H	2.53727	-2.92486	2.63648
H	-3.64886	-4.14999	-1.31223
H	0.69795	-4.65601	1.65242

C	-3.75348	0.44899	-1.71815
C	1.88853	-0.21282	2.02394
C	-1.36698	-3.29959	0.20288
C	-0.64508	3.53666	0.32322
H	-4.63634	0.55197	-2.34231
H	2.80885	-0.31791	2.58992
H	-1.49190	-4.37843	0.20333
H	-0.54724	4.61716	0.36401
O	0.02098	0.06344	-1.41449
S	-2.12409	0.32998	2.22614
C	-3.02480	-1.21755	2.57513
H	-2.33767	-2.06774	2.63474
H	-3.52437	-1.10290	3.54174
H	-3.77140	-1.42317	1.80303
H	0.19876	-0.85789	-1.65841
C	2.47070	-2.73387	-1.90767
O	2.46412	-1.37371	-2.04678
H	1.65762	-3.20461	-2.44693
H	2.83013	-3.13889	-0.96513
C	3.50703	-0.64022	-1.52223
C	4.76758	-1.18408	-1.29711
C	3.23646	0.74423	-1.26631
C	5.80322	-0.38308	-0.80460
H	4.94533	-2.22600	-1.54337
C	4.31457	1.52178	-0.76721
O	2.08085	1.31343	-1.49079
C	5.56961	0.97309	-0.54340
H	6.78574	-0.81602	-0.64123
H	4.10848	2.56901	-0.57059
H	1.10743	0.65841	-1.48939
H	6.37318	1.59993	-0.16587

TS2-a quartet

Fe	1.15643	-0.03839	0.25487
N	-0.00323	0.34204	1.86534
N	1.31471	1.94612	-0.10661
N	0.92806	-2.02174	0.56283
N	2.39301	-0.42405	-1.28497
C	-0.63030	-0.57733	2.66397
C	1.98320	2.53811	-1.14375
C	-0.36587	1.58033	2.33489
C	0.75800	2.95920	0.62749
C	0.18098	-2.61956	1.55007
C	2.91087	0.49488	-2.16585
C	1.56044	-3.03963	-0.11120
C	2.80576	-1.65712	-1.72447
C	-1.39569	0.09802	3.68398
C	1.83774	3.97394	-1.06940
C	-1.22846	1.43513	3.48221
C	1.08168	4.23494	0.03180
C	0.31934	-4.05393	1.47120
C	3.69520	-0.17780	-3.17114
C	1.17697	-4.31365	0.44698
C	3.63565	-1.51056	-2.89433
H	-1.97958	-0.40159	4.44657
H	2.27025	4.67411	-1.77291
H	-1.64882	2.25985	4.04364
H	0.76210	5.19393	0.41946
H	-0.17379	-4.75516	2.13234
H	4.21428	0.31990	-3.98056
H	1.53177	-5.27234	0.09056
H	4.09254	-2.33229	-3.43112
C	-0.55623	-1.95776	2.52076
C	2.72317	1.86937	-2.11156
C	2.44136	-2.87859	-1.17038
C	-0.00987	2.79903	1.77343
H	-1.10138	-2.56513	3.23613
H	3.20285	2.46927	-2.87872
H	2.85749	-3.77565	-1.61781
H	-0.38110	3.69757	2.25621
O	-0.30436	-0.02147	-0.86293
S	2.91407	0.08399	1.78807
C	4.45631	0.52270	0.91397
H	4.34532	1.48940	0.41422
H	5.25712	0.59369	1.65571
H	4.71655	-0.23416	0.16973

H	-0.51286	-0.95667	-1.03870
C	-2.18494	0.48143	-2.60079
O	-3.36216	-0.18806	-2.50947
H	-1.61568	0.22869	-3.48379
H	-2.08372	1.42697	-2.08655
C	-4.14694	-0.02131	-1.37398
C	-3.64150	0.30570	-0.11917
C	-5.51876	-0.24812	-1.57756
C	-4.52861	0.42483	0.95548
H	-2.57051	0.43282	0.00948
C	-6.39185	-0.12775	-0.49768
O	-5.99930	-0.57612	-2.81034
C	-5.89485	0.21098	0.76361
H	-4.14003	0.67419	1.93823
H	-7.45069	-0.29827	-0.66556
H	-5.23909	-0.60209	-3.41707
H	-6.58332	0.30298	1.59914

Fe-Porph-OH - singlet

Fe	0.08287	0.09076	-0.18846
N	1.70630	-1.16496	-0.15832
N	-1.16415	-1.58553	-0.21592
N	1.27579	1.63492	-0.15317
N	-1.53293	1.25232	-0.23805
C	3.02215	-0.80276	-0.07939
C	-2.52212	-1.59281	-0.31164
C	1.68879	-2.53985	-0.18064
C	-0.76441	-2.89763	-0.26591
C	2.65429	1.63873	-0.08041
C	-2.85020	0.86222	-0.32107
C	0.89646	2.95880	-0.16665
C	-1.54453	2.62017	-0.25797
C	3.86710	-1.97142	-0.05705
C	-3.01428	-2.95238	-0.39527
C	3.03775	-3.05063	-0.12470
C	-1.92146	-3.76165	-0.36817
C	3.14525	2.99548	-0.05059
C	-3.70971	2.02325	-0.37762
C	2.05925	3.81138	-0.10610
C	-2.89980	3.11490	-0.33785
H	4.94805	-1.95408	0.00038
H	-4.05789	-3.22985	-0.47408
H	3.29978	-4.10110	-0.13230
H	-1.88159	-4.84240	-0.41961
H	4.19155	3.26809	0.00435
H	-4.78970	1.98650	-0.44486
H	2.02759	4.89349	-0.10496
H	-3.17457	4.16166	-0.36575
C	3.46687	0.51686	-0.03997
C	-3.31366	-0.44587	-0.34730
C	-0.40927	3.42155	-0.21884
C	0.55114	-3.33984	-0.24452
H	4.53833	0.68234	0.02207
H	-4.38822	-0.58613	-0.42191
H	-0.55545	4.49744	-0.23312
H	0.71006	-4.41377	-0.27881
O	-0.00391	-0.15987	-1.95976
S	0.05821	-0.33640	2.01356
C	-1.60046	-0.03101	2.70283
H	-2.33222	-0.72565	2.27828
H	-1.54120	-0.20596	3.78162
H	-1.92979	0.99247	2.50835
H	0.85058	-0.54367	-2.22721

Fe-Porph-OH - triplet

Fe	0.07642	0.03047	-0.28131
N	1.87108	-0.89234	-0.17717
N	-0.85447	-1.75637	-0.22549
N	1.00864	1.80919	-0.11437
N	-1.72705	0.94708	-0.28175
C	3.10820	-0.30724	-0.03377
C	-2.21077	-1.99238	-0.25562
C	2.10061	-2.24122	-0.25369
C	-0.25268	-2.99429	-0.29728
C	2.35067	2.04395	0.01720

C	-2.95835	0.36303	-0.33866
C	0.41247	3.04001	-0.17915
C	-1.94482	2.29900	-0.34821
C	4.14153	-1.31492	-0.03209
C	-2.46733	-3.41209	-0.30472
C	3.51664	-2.51527	-0.17847
C	-1.25597	-4.03077	-0.33880
C	2.61251	3.46215	0.04608
C	-3.99294	1.36832	-0.43658
C	1.40604	4.08229	-0.08318
C	-3.36132	2.57370	-0.44583
H	5.20045	-1.11337	0.06858
H	-3.45298	-3.85934	-0.32929
H	3.95645	-3.50374	-0.21812
H	-1.04297	-5.09089	-0.39235
H	3.59401	3.90833	0.14399
H	-5.05412	1.16139	-0.49323
H	1.19283	5.14341	-0.10980
H	-3.79635	3.56302	-0.51053
C	3.33367	1.05792	0.07614
C	-3.19162	-1.01174	-0.29901
C	-0.95600	3.27220	-0.29851
C	1.11402	-3.22073	-0.33430
H	4.36280	1.38670	0.18595
H	-4.22478	-1.34360	-0.34299
H	-1.27748	4.30861	-0.34524
H	1.44433	-4.25338	-0.39479
O	0.07141	0.09206	-2.06954
S	0.02131	-0.35882	2.17870
C	-1.65322	-0.03120	2.80275
H	-2.35886	-0.72373	2.32754
H	-1.67242	-0.20122	3.88299
H	-1.96843	0.98982	2.57238
H	1.00273	0.14310	-2.34827

Fe=O-Porph - doblet

Fe	0.09001	0.04213	-0.36230
N	1.85561	-0.91507	-0.14137
N	-0.85946	-1.74466	-0.26081
N	1.02135	1.81096	-0.13520
N	-1.69352	0.98033	-0.29018
C	3.09603	-0.33521	-0.04630
C	-2.21650	-1.96098	-0.33048
C	2.07516	-2.26630	-0.20461
C	-0.27579	-2.99282	-0.30557
C	2.37035	2.02263	-0.03347
C	-2.93239	0.40160	-0.37143
C	0.44628	3.05350	-0.17221
C	-1.90782	2.33688	-0.31952
C	4.12324	-1.34905	-0.03748
C	-2.49372	-3.37588	-0.38397
C	3.48944	-2.54821	-0.14220
C	-1.29180	-4.01358	-0.37375
C	2.65560	3.43696	0.00387
C	-3.95735	1.41528	-0.43884
C	1.45873	4.07841	-0.08606
C	-3.32040	2.61780	-0.40765
H	5.18468	-1.14989	0.03703
H	-3.48552	-3.80699	-0.43432
H	3.92107	-3.54060	-0.16883
H	-1.09269	-5.07702	-0.41026
H	3.64697	3.86392	0.08620
H	-5.01933	1.21486	-0.50274
H	1.26084	5.14271	-0.09160
H	-3.75032	3.61082	-0.44037
C	3.34041	1.02846	0.01904
C	-3.18338	-0.96714	-0.37529
C	-0.91718	3.30603	-0.26237
C	1.08664	-3.23934	-0.29289
H	4.37546	1.34644	0.09875
H	-4.22017	-1.28302	-0.44097
H	-1.23209	4.34481	-0.28732
H	1.40798	-4.27526	-0.33853
O	0.16134	0.05021	-1.98164
S	-0.02287	-0.39701	2.22638

C	-1.70575	-0.05646	2.81715
H	-2.40211	-0.74305	2.31979
H	-1.75557	-0.23660	3.89501
H	-2.01065	0.96742	2.58591

TS1-b doblet

Fe	-0.99210	0.02339	0.15415
N	-2.07667	1.44665	-0.79595
N	0.07754	1.49310	1.04858
N	-2.19485	-1.41588	-0.56544
N	0.01326	-1.37176	1.20389
C	-3.12298	1.22528	-1.65345
C	1.13766	1.31935	1.90637
C	-1.86370	2.79804	-0.80016
C	0.00280	2.84851	0.80828
C	-3.23295	-1.23852	-1.44064
C	1.08683	-1.15400	2.02752
C	-2.11754	-2.76597	-0.33870
C	-0.18772	-2.73273	1.19735
C	-3.58586	2.47540	-2.21234
C	1.71682	2.59704	2.24727
C	-2.80068	3.45241	-1.68467
C	1.01570	3.54196	1.56503
C	-3.82986	-2.50935	-1.77356
C	1.58427	-2.40563	2.54604
C	-3.13634	-3.45978	-1.08728
C	0.78995	-3.38638	2.03289
H	-4.40344	2.57192	-2.91532
H	2.56125	2.73206	2.91103
H	-2.83908	4.51958	-1.86192
H	1.16421	4.61408	1.55385
H	-4.66853	-2.63972	-2.44560
H	2.42757	-2.50352	3.21781
H	-3.28711	-4.53180	-1.07926
H	0.84705	-4.45507	2.19663
C	-3.66798	-0.01648	-1.94717
C	1.60838	0.09477	2.36011
C	-1.18593	-3.38636	0.48705
C	-0.89317	3.45873	-0.05350
H	-4.50516	-0.03956	-2.63843
H	2.46571	0.11041	3.02623
H	-1.23733	-4.46749	0.57515
H	-0.83121	4.53757	-0.15667
O	0.11130	-0.01642	-1.21133
S	-2.48093	0.57145	2.05690
C	-1.79802	-0.15766	3.57544
H	-0.81727	0.28556	3.78579
H	-2.46819	0.07312	4.40860
H	-1.67438	-1.23914	3.47627
H	0.72845	-1.05597	-1.39587
C	1.63095	-1.90637	-1.81548
O	2.56261	-1.16759	-2.52412
H	1.08896	-2.56295	-2.49865
H	2.03169	-2.39976	-0.92435
C	3.60007	-0.58372	-1.79922
C	4.79460	-1.28891	-1.65926
C	3.46635	0.72753	-1.29579
C	5.89464	-0.70274	-1.03337
H	4.85056	-2.29098	-2.07555
C	4.58766	1.30493	-0.67724
O	2.34984	1.47645	-1.40620
C	5.78374	0.60343	-0.54889
H	6.82487	-1.25463	-0.93610
H	4.48290	2.31762	-0.29956
H	1.52337	0.92666	-1.47618
H	6.63335	1.07961	-0.06618

TS1-b quartet

Fe	-0.97195	0.03262	0.14685
N	-1.99798	1.55149	-0.71894
N	0.15773	1.38786	1.11309
N	-2.27697	-1.30812	-0.60430
N	-0.02828	-1.46732	1.11458
C	-3.06677	1.42539	-1.56848
C	1.21388	1.12059	1.95728

C	-1.71976	2.88736	-0.65831
C	0.14514	2.76051	0.94681
C	-3.31125	-1.03543	-1.45813
C	1.06131	-1.34925	1.93465
C	-2.25559	-2.66993	-0.45769
C	-0.29447	-2.81506	1.03309
C	-3.47129	2.72353	-2.06056
C	1.84780	2.35046	2.36358
C	-2.63116	3.63247	-1.49736
C	1.18875	3.36223	1.73633
C	-3.97040	-2.25652	-1.85214
C	1.50723	-2.65053	2.37259
C	-3.31324	-3.27459	-1.22884
C	0.66223	-3.56163	1.81425
H	-4.29221	2.89577	-2.74496
H	2.69967	2.41164	3.02872
H	-2.61768	4.70781	-1.62060
H	1.38689	4.42535	1.78263
H	-4.81985	-2.31131	-2.52103
H	2.35182	-2.82604	3.02672
H	-3.51111	-4.33772	-1.28033
H	0.67035	-4.63955	1.91551
C	-3.68519	0.23048	-1.90455
C	1.63791	-0.14480	2.33543
C	-1.33602	-3.37920	0.30964
C	-0.71564	3.45885	0.11890
H	-4.53084	0.28319	-2.58378
H	2.49997	-0.20218	2.99301
H	-1.43555	-4.46027	0.33547
H	-0.59881	4.53705	0.07297
O	0.07538	-0.03521	-1.26246
S	-2.40988	0.57047	2.12753
C	-1.79558	-0.34808	3.56988
H	-0.77805	-0.01240	3.80508
H	-2.43841	-0.13701	4.42917
H	-1.76541	-1.42259	3.37175
H	0.69719	-1.06930	-1.43781
C	1.61608	-1.91508	-1.86234
O	2.54029	-1.14801	-2.55418
H	1.08974	-2.56848	-2.56046
H	2.02091	-2.41716	-0.97803
C	3.56483	-0.56235	-1.81463
C	4.76128	-1.26034	-1.65613
C	3.41556	0.74755	-1.31180
C	5.84804	-0.66685	-1.01392
H	4.83030	-2.26190	-2.07172
C	4.52338	1.33277	-0.67677
O	2.29330	1.48544	-1.43703
C	5.72191	0.63871	-0.53129
H	6.78055	-1.21241	-0.90341
H	4.40643	2.34422	-0.29950
H	1.47453	0.92457	-1.51989
H	6.56155	1.12017	-0.03648

TS1-a doblet

Fe	1.08699	0.09282	0.08145
N	0.96657	2.05110	-0.38135
N	1.60880	-0.33764	-1.83337
N	0.72307	0.54843	2.00592
N	1.38357	-1.84263	0.56973
C	0.75225	3.10427	0.47430
C	1.91707	-1.56724	-2.35797
C	1.03780	2.59283	-1.63940
C	1.57733	0.53321	-2.89571
C	0.55080	1.79249	2.55626
C	1.70667	-2.87374	-0.27020
C	0.61206	-0.34145	3.04308
C	1.15341	-2.40986	1.79722
C	0.68853	4.34275	-0.26368
C	2.11674	-1.46522	-3.78621
C	0.85372	4.02351	-1.57681
C	1.89738	-0.16524	-4.11998
C	0.31015	1.68566	3.97546
C	1.70421	-4.12646	0.44874
C	0.33890	0.35800	4.27662
C	1.35595	-3.83872	1.73300

H	0.53214	5.31828	0.17900
H	2.37561	-2.29331	-4.43380
H	0.86532	4.68410	-2.43443
H	1.94347	0.29621	-5.09823
H	0.14317	2.52481	4.63885
H	1.93309	-5.09004	0.01141
H	0.20484	-0.11801	5.23975
H	1.24154	-4.51695	2.56913
C	0.58224	2.99078	1.84860
C	1.98250	-2.74825	-1.62997
C	0.79105	-1.71783	2.94619
C	1.29424	1.88808	-2.81163
H	0.43221	3.90733	2.41140
H	2.22732	-3.65613	-2.17330
H	0.65967	-2.29907	3.85419
H	1.30884	2.45659	-3.73664
O	-0.56746	-0.25592	-0.34903
S	3.50745	0.61433	0.20542
C	4.46901	-0.92831	0.21556
H	4.29209	-1.47624	-0.71756
H	5.53266	-0.67932	0.27426
H	4.18310	-1.57037	1.05270
H	-1.37444	0.45060	0.12689
C	-2.40460	1.10954	0.71825
O	-3.52345	1.12159	-0.09548
H	-2.04316	2.12930	0.83948
H	-2.54650	0.55347	1.65013
C	-4.25217	-0.04487	-0.24940
C	-3.73618	-1.32954	-0.09918
C	-5.59686	0.16216	-0.60892
C	-4.58221	-2.42638	-0.29215
H	-2.68205	-1.46551	0.12052
C	-6.42911	-0.93884	-0.80006
O	-6.08772	1.42443	-0.76110
C	-5.92035	-2.22986	-0.63725
H	-4.18513	-3.43126	-0.18345
H	-7.46603	-0.76506	-1.07021
H	-5.35196	2.03715	-0.58617
H	-6.57503	-3.08361	-0.78858

TS1-a quartet

Fe	-1.10231	0.06416	-0.10300
N	-2.06495	-1.64227	0.40900
N	0.31875	-1.07318	-1.04236
N	-2.55254	1.16265	0.73663
N	-0.23346	1.72983	-0.79918
C	-3.20534	-1.72609	1.16459
C	1.42306	-0.60585	-1.70462
C	-1.67249	-2.93570	0.16247
C	0.38533	-2.44487	-1.10741
C	-3.62275	0.69339	1.44985
C	0.96204	1.81334	-1.47064
C	-2.65689	2.52726	0.74655
C	-0.63894	3.02188	-0.58039
C	-3.55145	-3.10852	1.39127
C	2.21313	-1.70929	-2.20180
C	-2.60099	-3.85881	0.76857
C	1.56630	-2.85038	-1.83224
C	-4.42167	1.79694	1.93693
C	1.30624	3.19569	-1.70060
C	-3.82369	2.93567	1.49738
C	0.31028	3.94544	-1.15334
H	-4.41216	-3.44361	1.95607
H	3.13647	-1.60522	-2.75715
H	-2.51989	-4.93719	0.71663
H	1.84478	-3.87781	-2.03105
H	-5.32187	1.69156	2.52910
H	2.20014	3.53034	-2.21144
H	-4.12767	3.96285	1.65409
H	0.21754	5.02359	-1.12053
C	-3.93095	-0.64512	1.65305
C	1.73303	0.73741	-1.89379
C	-1.77594	3.40044	0.12341
C	-0.53765	-3.31629	-0.54485
H	-4.81910	-0.86788	2.23666
H	2.65450	0.96491	-2.42085

H	-1.97600	4.46381	0.21352
H	-0.35928	-4.38061	-0.66519
O	-0.20979	0.13132	1.38612
S	-2.31960	-0.28327	-2.17447
C	-1.50701	0.63185	-3.52024
H	-0.48383	0.26227	-3.65113
H	-2.06205	0.45320	-4.44573
H	-1.46489	1.70212	-3.30339
H	0.61446	-0.66990	1.57459
C	1.57902	-1.55099	2.02212
O	2.73093	-1.46218	1.26361
H	1.13789	-2.53499	1.87313
H	1.71229	-1.26537	3.06983
C	3.57924	-0.38535	1.39324
C	3.36082	0.73113	2.19656
C	4.73911	-0.48859	0.59933
C	4.33082	1.73821	2.24257
H	2.43534	0.83313	2.75199
C	5.69997	0.51765	0.65772
O	4.92126	-1.56143	-0.22106
C	5.49693	1.62621	1.48458
H	4.16291	2.61095	2.86638
H	6.59276	0.41827	0.04810
H	4.10986	-2.09913	-0.17803
H	6.24997	2.40818	1.52419

Fe=O-Porph - quartet

Fe	0.08895	0.03952	-0.36576
N	1.86276	-0.90115	-0.14522
N	-0.84332	-1.75056	-0.26510
N	1.00580	1.81862	-0.12816
N	-1.70236	0.96435	-0.29294
C	3.09813	-0.31000	-0.04804
C	-2.19942	-1.97867	-0.33111
C	2.09490	-2.25008	-0.20789
C	-0.24962	-2.99497	-0.30398
C	2.35278	2.04153	-0.02887
C	-2.93637	0.37652	-0.37754
C	0.42013	3.05592	-0.16389
C	-1.92741	2.31953	-0.31803
C	4.13428	-1.31466	-0.03929
C	-2.46453	-3.39574	-0.37792
C	3.51148	-2.51948	-0.14488
C	-1.25745	-4.02362	-0.36644
C	2.62631	3.45830	0.00914
C	-3.96936	1.38196	-0.44505
C	1.42390	4.08957	-0.07820
C	-3.34204	2.58935	-0.40862
H	5.19381	-1.10596	0.03651
H	-3.45284	-3.83511	-0.42546
H	3.95197	-3.50797	-0.17119
H	-1.04971	-5.08554	-0.39877
H	3.61423	3.89349	0.08978
H	-5.02956	1.17322	-0.51164
H	1.21688	5.15213	-0.08305
H	-3.77974	3.57904	-0.43907
C	3.33110	1.05529	0.02080
C	-3.17533	-0.99432	-0.38054
C	-0.94542	3.29684	-0.25451
C	1.11433	-3.23131	-0.29390
H	4.36354	1.38158	0.10089
H	-4.20904	-1.32004	-0.44639
H	-1.26930	4.33292	-0.27674
H	1.44343	-4.26486	-0.33763
O	0.17228	0.08273	-1.98469
S	-0.02234	-0.40009	2.22592
C	-1.70860	-0.07328	2.81526
H	-2.39975	-0.76382	2.31616
H	-1.75761	-0.25730	3.89258
H	-2.02090	0.94912	2.58760

Fe-Porph - quartet

Fe	0.07131	-0.00002	-0.09375
N	1.50237	1.41111	-0.28562
N	1.49805	-1.41542	-0.28603

N	-1.32688	1.41541	-0.41326
N	-1.33122	-1.41125	-0.41313
C	1.31649	2.77591	-0.28416
C	1.30806	-2.77965	-0.28486
C	2.86554	1.22356	-0.25367
C	2.86180	-1.23200	-0.25426
C	-1.13636	2.78044	-0.40311
C	-1.14483	-2.77682	-0.40296
C	-2.68790	1.23229	-0.52823
C	-2.69168	-1.22398	-0.52798
C	2.58557	3.45572	-0.25350
C	2.57509	-3.46331	-0.25468
C	3.54713	2.49215	-0.23718
C	3.53957	-2.50265	-0.23830
C	-2.39868	3.46365	-0.50946
C	-2.40925	-3.45622	-0.50902
C	-3.36124	2.50327	-0.59023
C	-3.36890	-2.49292	-0.58978
H	2.70721	4.53137	-0.25060
H	2.69344	-4.53933	-0.25212
H	4.62284	2.61199	-0.21632
H	4.61491	-2.62572	-0.21772
H	-2.51589	4.53969	-0.52726
H	-2.52975	-4.53189	-0.52666
H	-4.43235	2.62786	-0.68653
H	-4.44039	-2.61429	-0.68595
C	0.09075	3.42073	-0.32876
C	0.08038	-3.42080	-0.32907
C	-3.33152	0.00513	-0.57990
C	3.50711	-0.00521	-0.23675
H	0.09249	4.50607	-0.32609
H	0.07890	-4.50614	-0.32645
H	-4.41308	0.00681	-0.67103
H	4.59206	-0.00689	-0.20822
S	0.04679	-0.00017	2.31706
C	-1.71971	0.00094	2.80806
H	-2.23434	-0.88977	2.43622
H	-1.76612	0.00169	3.90147
H	-2.23356	0.89160	2.43504

Fe-Porph - sextet

Fe	0.06648	-0.00043	0.11002
N	1.58852	-1.38895	-0.27992
N	-1.30770	-1.49086	-0.42580
N	1.48926	1.48965	-0.28889
N	-1.40697	1.38806	-0.42936
C	2.94000	-1.14563	-0.27111
C	-2.66636	-1.34223	-0.57153
C	1.43305	-2.75426	-0.30969
C	-1.05336	-2.84183	-0.44397
C	2.85412	1.34068	-0.27975
C	-2.75235	1.14487	-0.57423
C	1.23963	2.84043	-0.32544
C	-1.24714	2.75373	-0.45321
C	3.66118	-2.39725	-0.28940
C	-3.29000	-2.64094	-0.67427
C	2.72895	-3.39234	-0.31122
C	-2.29251	-3.56817	-0.59307
C	3.48717	2.63934	-0.30587
C	-3.46448	2.39652	-0.68060
C	2.48836	3.56716	-0.33209
C	-2.53378	3.39135	-0.60322
H	4.73992	-2.49063	-0.29212
H	-4.35188	-2.81020	-0.80194
H	2.89263	-4.46233	-0.33738
H	-2.37623	-4.64659	-0.64237
H	4.55687	2.80709	-0.31018
H	-4.53561	2.49119	-0.80813
H	2.57732	4.64577	-0.36409
H	-2.69241	4.46114	-0.65635
C	3.52710	0.11943	-0.26073
C	-3.33734	-0.12036	-0.62788
C	-0.02542	3.42363	-0.38504
C	0.21176	-3.42447	-0.36977
H	4.61251	0.15686	-0.25337
H	-4.41642	-0.15769	-0.74602

H	-0.06192	4.50876	-0.41610
H	0.25130	-4.50962	-0.39572
S	0.00927	-0.00705	2.43872
C	-1.77714	0.02126	2.86568
H	-2.29767	-0.82570	2.41147
H	-1.86556	-0.04597	3.95329
H	-2.23991	0.95121	2.52542

H2O

O	-2.65779	1.35823	0.00000
H	-1.69039	1.40574	0.00000
H	-2.93594	2.28600	0.00000
